# Microbiome Learning Repo (ML Repo): A public repository of microbiome regression and classification tasks

**DOI:** 10.1093/gigascience/giz042

**Published:** 2019-04-26

**Authors:** Pajau Vangay, Benjamin M Hillmann, Dan Knights

**Affiliations:** 1 Bioinformatics and Computational Biology, University of Minnesota, 200 Union Street SE, Minneapolis, MN 55455; 2 Department of Computer Science and Engineering, University of Minnesota, 200 Union Street SE, Minneapolis, MN 55455

**Keywords:** microbiome, machine learning, repository, database

## Abstract

The use of machine learning in high-dimensional biological applications, such as the human microbiome, has grown exponentially in recent years, but algorithm developers often lack the domain expertise required for interpretation and curation of the heterogeneous microbiome datasets. We present Microbiome Learning Repo (ML Repo, available at https://knights-lab.github.io/MLRepo/), a public, web-based repository of 33 curated classification and regression tasks from 15 published human microbiome datasets. We highlight the use of ML Repo in several use cases to demonstrate its wide application, and we expect it to be an important resource for algorithm developers.

## Findings

### Background

Machine learning is widely used as a method for classification and prediction, with a growing number of applications in human health [1]. The use of machine learning in biological fields [2, 3], and more specifically the microbiome research field [4–7], has grown exponentially owing to the robustness of these algorithms to high-dimensional data. However, challenges exist for large-scale meta-analysis because they often require manual curation of metadata and standardized processing of raw sequence data, resulting in variation in the results derived from chosen datasets across studies [8, 9]. In addition, microbiome research data can be challenging to access and analyze for expert machine learning algorithm developers, who often do not have the domain expertise required to interpret the data and metadata in complex microbiome studies. There exist general resources with curated classification tasks from a variety of domains. The University of California Irvine Machine Learning Repository [10] revolutionized machine learning methods development by giving developers access to many curated datasets; its widespread usage and impact can be seen from its thousands of resulting citations. Currently, we are unaware of any machine learning repository dedicated to microbiome classification tasks. We constructed a complementary database to address this deficiency, in order to promote the development of and use of improved machine learning methods for the microbiome community.

### Workflow

**Figure 1:**
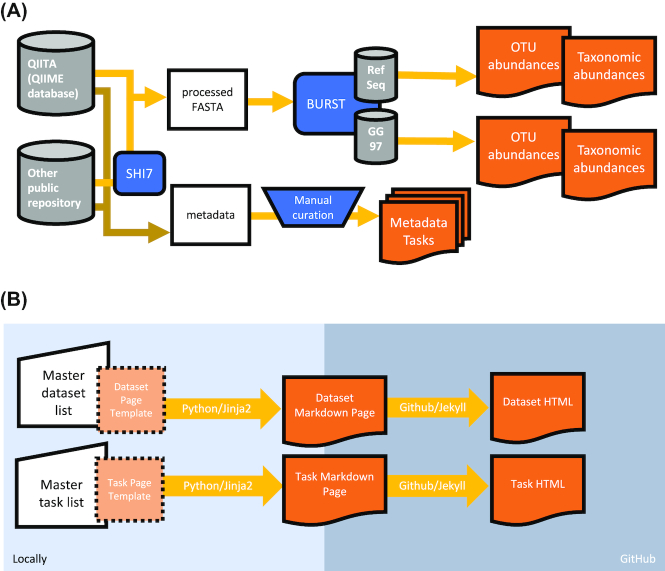
Data processing workflow and website generation. (A) Quality-filtered sequences were obtained from either the QIITA or from another public repository and trimmed and filtered using SHI7. Reference-based OTUs were picked using BURST with the NCBI RefSeq and Greengenes 97 (GG 97) databases. (B) Individual GitHub Markdown pages were generated from dataset and task lists with a custom Python script and Jinja2 template, then uploaded to GitHub to be hosted.

We present the Microbiome Learning Repo (ML Repo), a repository of 33 curated classification and regression tasks involving human microbiome data. Our 33 tasks are derived from 15 publicly available human microbiome datasets, which include 12 amplicon-based and 3 shotgun sequencing datasets (Table 1). These datasets vary across sequencing technology platforms, 16s hypervariable regions, and study design, in order to help developers ensure robustness of algorithms across data types. We streamlined the microbiome data using a single post-processing workflow (Fig. 1A). We downloaded trimmed and quality-filtered sequencing reads for 8 datasets from QIITA [11], and raw sequences for 7 datasets from public repositories. Raw sequences were trimmed and quality filtered using SHI7 [12] or QIIME [13]. We picked operational taxonomic units (OTUs) from all quality-filtered sequences using a closed-reference method with the BURST [14] aligner against both the National Center for Biotechnology Information (NCBI) RefSeq 16S ribosomal RNA project [15] and the Greengenes 97 database [16]. Samples with <1,000 sequencing reads were dropped for 10 datasets, while we applied a lower threshold of 100 sequencing reads per sample for 5 datasets that had lower expected bacterial load. Full details regarding the data preprocessing are provided for each data set in the *mlrepo-source* branch of the GitHub repository, under preprocessing/make.mappings.r. As a result, for each dataset we generated RefSeq-based OTU and taxa abundance counts, and Greengenes-based OTU and taxa abundance counts. These counts are presented in tables that are organized as follows: OTUs or taxa as rows, and samples as columns. OTUs are represented as either NCBI genome identifiers or Greengenes identifiers. Taxa are represented as “kingdom; phylum; class; order; family; genus; species; strain," with highest taxonomic specificity where possible. We excluded additional post-processing filtering and normalization steps so that these parameters can be included in future benchmarking use cases as needed. We also limit our data to OTU and taxa tables because other metrics such as α and β diversity can be subsequently generated as needed.

**Table 1:** Microbiome datasets with available classification tasks in ML Repo

Project name	V Region	Target size	No. samples	No. subjects	Area	Description	Sequencing technology	Study design
Cho 2012	V3	177	95	47	Antibiotics	Mouse fecal and cecal samples, control vs 4 kinds of antibiotics	454	Cross-sectional
Claesson 2012	V4	221	168	168	Age	Elderly and young adults	454	Cross-sectional
David 2014	V4	282	235	11	Diet	Plant-based vs animal-based diet, cross-over study	Illumina MiSeq	Longitudinal
Gevers 2014	V4	173	1,321	668	IBD	Biopsies from patients with IBD prior to treatment	Illumina MiSeq	Cross-sectional
HMP 2012	V35	527	6,407	242	Body habitat, sex	Up to 18 body sites across 242 healthy subjects at 1–2 time points	454	Cross-sectional
Kostic 2012	V35	569	190	95	Colorectal cancer	Adjacent healthy vs tumor colon biopsy tissues	454	Paired
Montassier 2016	V56	280	28	28	Bacteremia	Patients prior to chemotherapy who did or did not develop bacteremia	454	Cross-sectional
Morgan 2012	V35	569	231	231	IBD	Healthy controls, patients with Crohn's disease or ulcerative colitis	454	Cross-sectional
Turnbaugh 2009	V2	230	281	154	Obesity	Monozygotic or dizygotic twin pairs concordant for body mass index class, and their mothers	454	Cross-sectional
Wu 2011	V12	244	95	10	Diet	Controlled high-fat or low-fat feeding on 10 subjects over 10 days	454	Longitudinal
Yatsunenko 2012	V4	282	531	531	Geography, age, sex	Humans of varying ages from the USA, Malawi, and Venezuela	Illumina MiSeq	Cross-sectional
Ravel 2011	V12	240	396	396	Bacterial vaginosis	Vaginal samples from 4 ethnic groups; Nugent scores for bacterial vaginosis	454	Cross-sectional
Karlsson 2013	NA	NA	144	144	Diabetes	Patients with normal, impaired, or type 2 diabetes glucose tolerance categories	Illumina HiSeq	Cross-sectional
Qin 2012	NA	NA	134	134	Diabetes	Chinese healthy controls vs patients with type 2 diabetes	Illumina HiSeq	Cross-sectional
Qin 2014	NA	NA	130	130	Cirrhosis	Healthy controls vs patients with cirrhosis	Illumina HiSeq	Cross-sectional

ML Repo contains 33 classification and regression tasks from 15 publicly available human microbiome datasets shown here. IBD: inflammatory bowel disease; NA: not applicable.

Sample metadata from individual studies were manually curated to generate viable prediction tasks. When available, published study exclusion criteria, such as reported use of antibiotics, were applied accordingly and confounders were removed by dropping samples or stratification. Well-known confounders were accounted for when constructing prediction tasks for other human-associated conditions; e.g., predicting age using the Yatsunenko 2012 dataset is restricted to samples from the USA owing to the known variation in gut microbiomes across different geographical locations. Details of how samples were subset for each prediction task can be found in the *mlrepo-source* branch of the GitHub repository, under preprocessing/make.mappings.r. Studies that were cross-sectional by design but contained several samples per subject were filtered to contain 1 sample per subject. In study designs with paired diseased-healthy or pre- and post-intervention samples, samples were reduced to 2 samples per subject with subject identifiers provided as confounder variables. Hence, each prediction task is made available as an individual, compartmentalized metadata file that contains sample identifiers, responses to predict, and optionally, confounder variables that are inherent to the research study design such as paired healthy and diseased samples from the same subject (see Methods for more details). As a result, we generated 33 distinct tasks for predicting human-associated responses.

### Publicly available web-based interface

We expect 2 types of users: (i) machine learning algorithm developers with limited knowledge of microbiome study designs and (ii) microbiome researchers interested in obtaining additional datasets for meta-analysis. Generally, we expect that method developers will be most interested in sweeping through the full set of prediction tasks for benchmarking, and hence would prefer to download a single compressed file containing all tasks and data. On the other hand, we expect microbiome researchers to be more selective in downloading specific datasets and tasks depending on their research domain. Hence, researchers may prefer to browse specific details about tasks and datasets prior to downloading.

**Figure 2:**
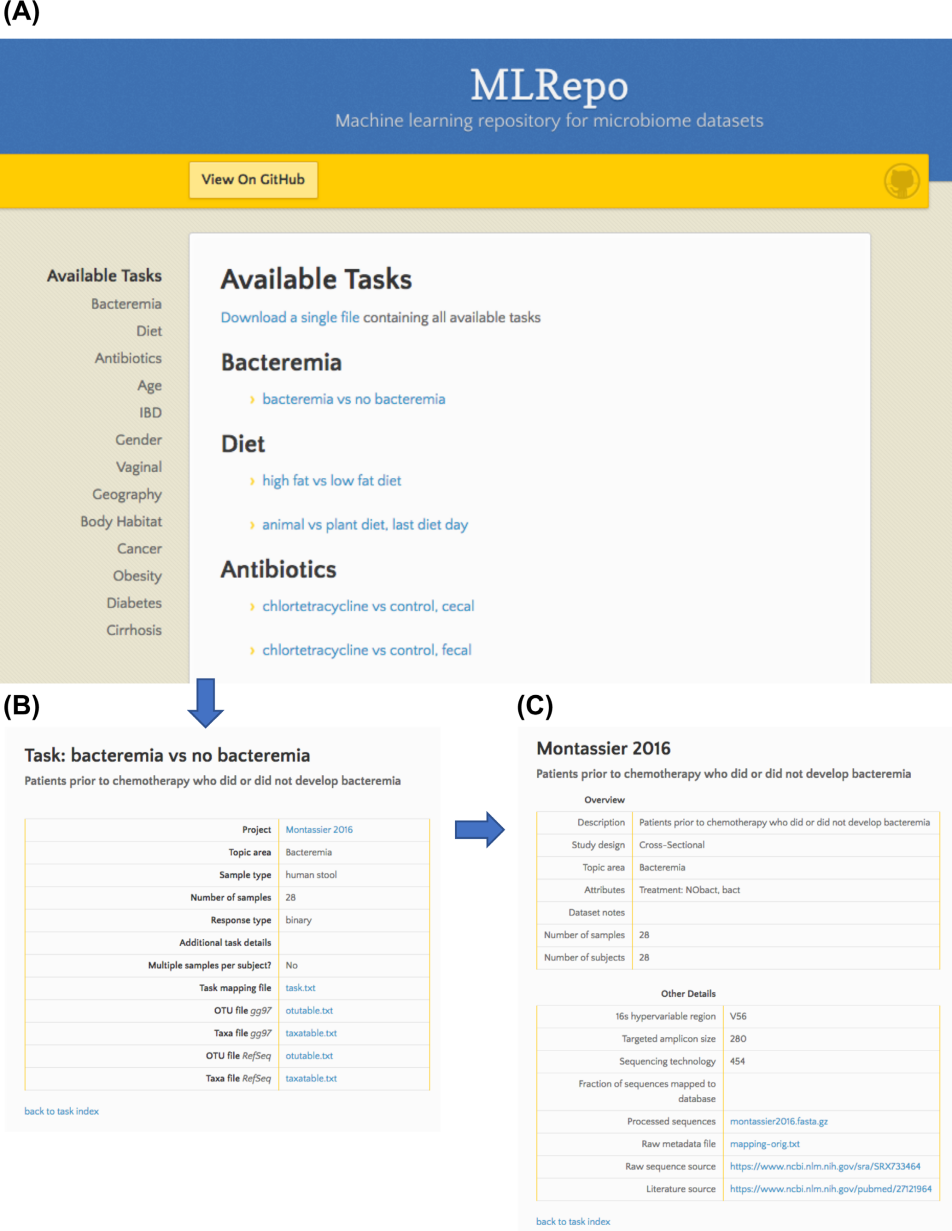
Screenshots of ML Repo web interface. (A) Available classification and regression tasks are listed by high-level phenotype categories for browsing. (B) Individual task webpages contain links to files for classifying a specific task, as well as relevant task-specific metadata. (C) Individual dataset webpages contain relevant metadata pertaining to the entire dataset, as well as links to raw metadata files and sequencing data.

On the basis of these expected use cases, we created a publicly available web-based interface for ML Repo hosted by GitHub Pages [17]. Tasks are organized by relevant response categories (Fig. 2A). Task pages contain descriptive details such as sample size and response type that are specific to the selected prediction task, as well as links for downloading OTU tables, taxa tables, and sample metadata (Fig. 2B). Dataset pages contain important details about the entire dataset, including links to the original research study, as well as original metadata files and quality-filtered sequences (Fig. 2C). We also provide a single compressed file containing the entire set of available tasks (OTU tables, taxa tables, and relevant metadata) for download from the main home page.

### Benefits of curated microbiome-based prediction tasks

We expect ML Repo to be beneficial for both the machine learning community as well as the microbiome research community. ML Repo will be a powerful complement to the University of California Irvine's Machine Learning Repository because it will allow for benchmarking curated classification tasks with high-dimensional data and hence enable the subsequent development of novel algorithms for these complex datasets. Our streamlined approach in generating OTU and taxa tables offers a rich set of 15 datasets that microbiome researchers can use directly for further comparison with their own studies, for teaching and learning purposes, or for large meta-analyses. We expect that our provided OTU and taxa tables will also be beneficial for researchers with limited access to high-performance computing resources or bioinformatics skills necessary for processing raw sequencing data. In addition, we also expect microbiome-specific methods development to benefit from our repository. The subset of samples found in each prediction task metadata file replace the work of rigorously deciphering metadata and understanding the subtle differences of individual research studies. New methods, such as OTU-picking algorithms, can be evaluated not only on metrics such as speed and accuracy but also based on overall impact to study findings.

### Comparison to similar databases

Although a number of microbiome repositories exist, many are intended as data archival repositories [18, 19] or function as resources for aggregating across studies [20]. Resources such as QIITA [11] offer an extensive collection of datasets, and mock-community–based Mockrobiota [21] is well-suited for benchmarking upstream methods, but neither offers support for the metadata interpretation necessary for predicting high-level phenotypes. Microbiome-based repositories that do provide manually curated metadata include curatedMetagenomicData [22] and MicrobiomeHD [23]. Although curatedMetagenomicData offers a collection of shotgun-metagenomics datasets with varying human sample types with gene, pathway, and taxonomic abundance tables, its data are accessible only via Bioconductor [24] and are stored as ExpressionSet objects, which integrates metadata and abundance data. Although curatedMetagenomicData is an impressive repository with many features, it is most suitable for advanced bioinformaticians because its interface may hinder use by beginner data analysts and in teaching environments. MicrobiomeHD offers easily accessible taxonomic abundance tables with curated metadata but is limited only to amplicon-based sequencing data, human stool samples, and case-control responses. And although both curatedMetagenomicData and MicrobiomeHD provide manually curated metadata, biological interpretation is still required because other sample metadata, e.g., antibiotic use, may have biological relevance in predicting responses. This poses a potential problem for machine learning developers with limited biological and microbiome domain expertise. ML Repo resolves this issue by explicitly defining classification and regressions tasks for predicting responses that have either been manually curated to remove confounders or been specifically annotated with biological confounders that must be controlled for. Metadata files in ML Repo are task-specific and, hence, are simplified to contain only (i) sample identifiers indicating samples that should be used for the prediction task, (ii) corresponding high-level phenotypes or responses, and optionally, (iii) a confounder that should be accounted for owing to its biological relevance. In addition, datasets in ML Repo include both amplicon-based and shotgun-metagenomics datasets covering a variety of human sample types, and are easily accessible via a web-based interface.

### Case studies

**Figure 3:**
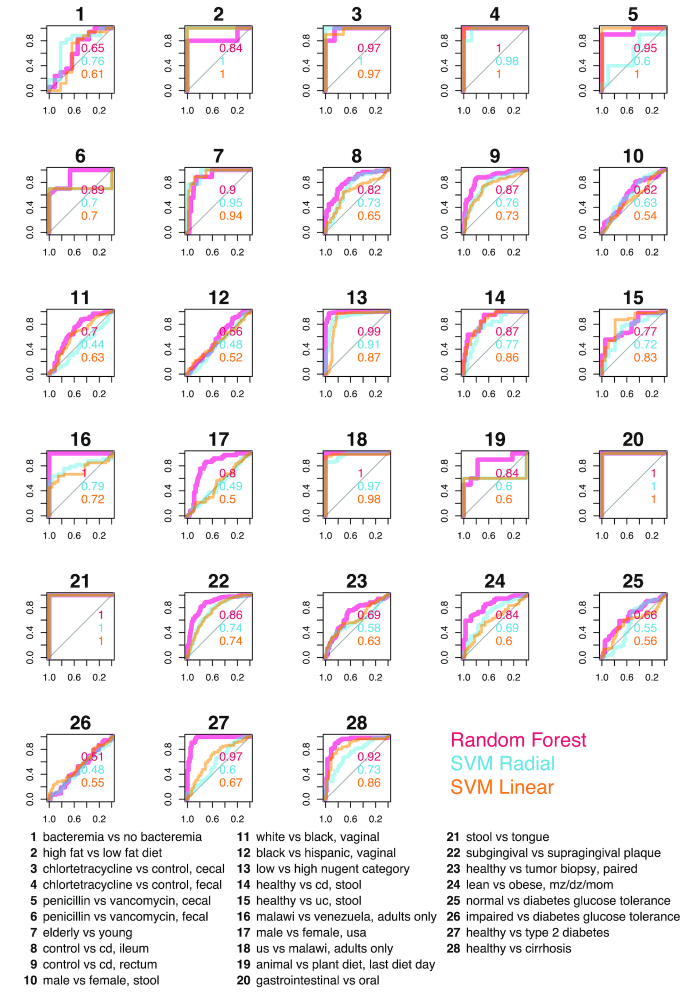
ROCs comparing random forest and SVM with different kernels. Sweeping across all binary classification tasks available in ML Repo (28), we compare ROCs of random forest, SVM with a radial kernel, and SVM with a linear kernel. AUCs are listed within plots and are colored respective to each model. cd: Crohn's disease; dz: dizygotic; mz: monozygotic; uc: ulcerative colitis.

**Figure 4:**
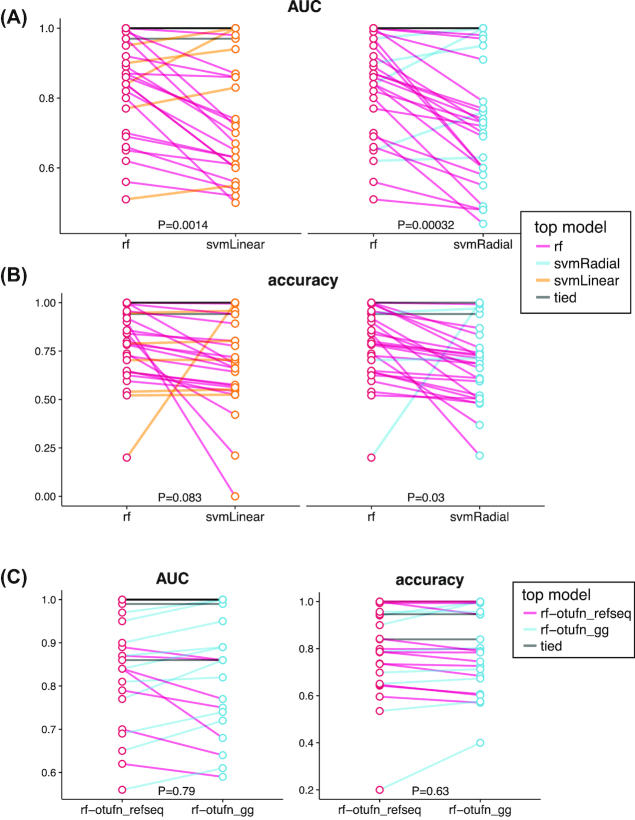
Summary statistics of framework and database comparisons. (A) AUCs of random forest (rf) to SVM-Linear (left) and random forest to SVM-Radial (right). Paired t-tests reveal that random forest results in significantly higher AUC than both SVM-Linear (*P* = 0.0014) and SVM-Radial (*P* = 0.00032). (B) Accuracies of random forest to SVM-Linear (left) and random forest to SVM-Radial (right). Paired t-tests reveal that random forest results in significantly better accuracy than SVM-Radial (*P* = 0.03), but not SVM-Linear (*P* = 0.083). (C) AUCs (left) and accuracies (right) of random forest classifications of 24 tasks using OTUs picked with NCBI RefSeq database or Greengenes (gg) database as predictors. Student t-test reveals that reference database choice has limited impact on classification AUC or accuracy. Lines are colored by the top model for each classification task.

We compare the performance of 3 machine learning models: a random forest [25], and a support vector machine(SVM) [26] with either a radial or linear kernel. Sweeping through available tasks with binary responses, we compare our models by examining receiver operating curves (ROCs) and areas under the curve (AUC), considered the standard method for machine learning model evaluation [27, 28] (Fig. 3). Through comparison of ROCs, we can see that random forest outperforms or ties the other 2 models in 21 of the 28 tasks. The choice of kernels for SVM seems to have limited impact on overall mean accuracy, yet a linear kernel was able to perfectly classify penicillin-treated and vancomycin-treated mouse cecal contents when the other models could not; further examination of the microbial features in these samples may be warranted to better elucidate the strengths of this kernel. We also performed pairwise comparisons of random forest against the other models across all tasks. When evaluated by AUC, random forest performed significantly better than both SVM with a linear kernel (*P* = 0.0014) and with a radial kernel (*P* = 0.00032) (Fig. 4A). We found that random forest accuracy improvements were moderate when compared with SVM-Linear (*P* = 0.083) and SVM-Radial (*P* = 0.03) (Fig. 4B), which may be explained by the fact that, unlike AUC, accuracy ignores class prediction probability estimates. Our results support the broad usage [4, 5, 8, 29] and acceptance of random forest as a robust classifier [6] with high-dimensional microbiome data.

**Figure 5:**
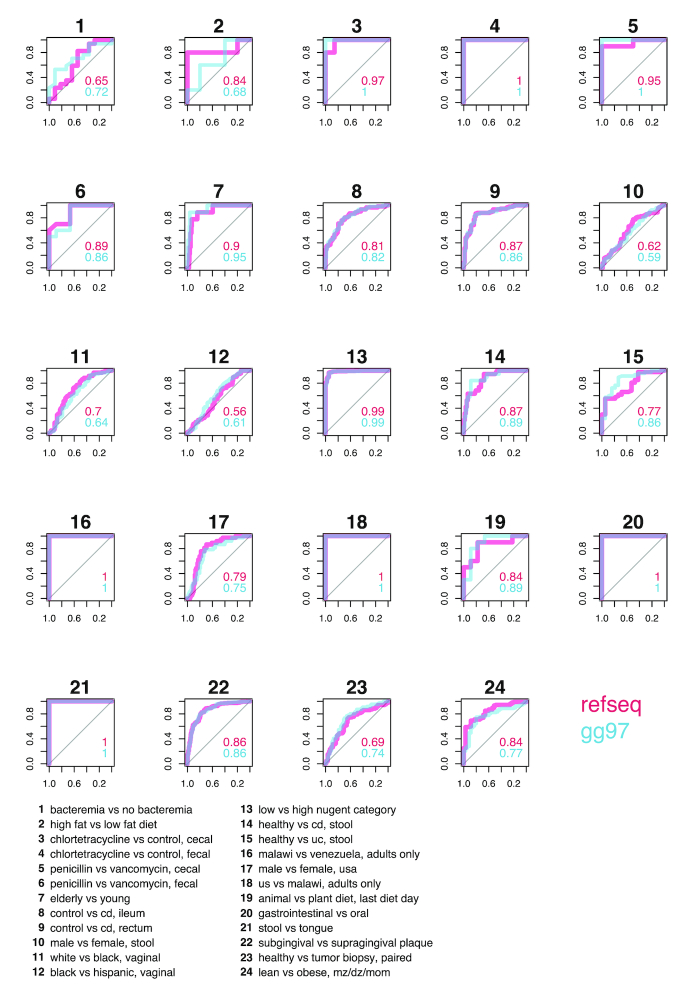
ROCs comparing NCBI RefSeq and Greengenes 97 (gg97) databases. Sweeping across 16s-based binary classification tasks available in ML Repo (24), we compare ROCs of random forest with genus-level taxonomic summaries as predictors from OTU-picking strategies with the NCBI RefSeq prokaryote reference database and the Greengenes 97 reference database. AUCs are listed within plots and are colored respective to each database. cd: Crohn's disease; dz: dizygotic; mz: monozygotic; uc: ulcerative colitis.

To assess the impact of reference database choice on classification accuracies, we also used the classification tasks to compare random forest using OTUs picked with the Greengenes 97 database or the NCBI RefSeq Targeted Loci Project 16s project. We found that there was limited impact of database choice on overall classification accuracies (Figs 4C and 5). This may be due to (i) large effect sizes that are driven mainly by several well-characterized bacterial taxa present in both databases (e.g., stool vs tongue samples), or (ii) small effect sizes such that classification is difficult regardless of the database (e.g., male vs female stool). Note that OTU-picking with the Greengenes database resulted in more OTU features in every dataset (Table 2); hence, these findings further highlight how the smaller, higher-quality NCBI RefSeq database can recover the same signal from the larger Greengenes database.

**Table 2:** Description of available prediction tasks

Dataset	Attributes	Description	Area	Regression?	Sample size	No. of Features				Control variable
						OTU, refseq	OTU, gg	Taxa, refseq	Taxa, gg	
Cho 2012	Abx: Control, Chlortetracycline	5 Groups of mice treated with 4 different antibiotics or no antibiotics	Antibiotics		47	293	1,144	299	141	N
	Abx: Control, Chlortetracycline	5 Groups of mice treated with 4 different antibiotics or no antibiotics	Antibiotics		45	293	1,144	299	141	N
	Abx: Penicillin, Vancomycin	5 Groups of mice treated with 4 different antibiotics or no antibiotics	Antibiotics		47	293	1,144	299	141	N
	Abx: Penicillin, Vancomycin	5 Groups of mice treated with 4 different antibiotics or no antibiotics	Antibiotics		45	293	1,144	299	141	N
Claesson 2012	AGE: Elderly, Young	Elderly or young adults	Age		167	569	3,763	662	279	N
David 2014	Diet: Plant, Animal	Individuals on the last day of an animal or plant diet intervention	Diet		18	1,747	6,293	1,535	695	Y
Gevers 2014	DIAGNOSIS: no, CD	Healthy controls and patients with CD	IBD		140	943	3,547	992	446	N
	DIAGNOSIS: no, CD	Healthy controls and patients with CD	IBD		160	943	3,547	992	446	N
	PCDAI	PCDAI scores of patients with CD at 6 months after sampling	IBD	X	68	943	3,547	992	446	N
	PCDAI	PCDAI scores of patients with CD at 6 months after sampling	IBD	X	51	943	3,547	992	446	N
HMP 2012	HMPBODYSUPERSITE: Oral, Gastrointestinal_tract, HOST_SUBJECT_ID	Gastrointestinal tract and oral cavity of healthy adults	Body habitat		2,070	3,121	9,383	3,090	1,218	Y
	SEX: male, female	Healthy male and female adults	Sex		180	3,121	9,383	3,090	1,218	N
	HMPBODYSUBSITE: Stool, Tongue_dorsum; HOST_SUBJECT_ID	Stool and tongue of healthy adults	Body habitat		404	3,121	9,383	3,090	1,218	Y
	HMPBODYSUBSITE: Subgingival_plaque, Supragingival_plaque; HOST_SUBJECT_ID	Subgingival and supragingival plaque of healthy adults	Body habitat		408	3,121	9,383	3,090	1,218	Y
Karlsson 2013	Classification: IGT, T2D	Impaired or type 2 diabetes glucose tolerance categories	Diabetes		101	12,845	NA	3,758	NA	N
	Classification: NGT, T2D	Normal or type 2 diabetes glucose tolerance categories	Diabetes		96	12,845	NA	3,758	NA	N
Kostic 2012	DIAGNOSIS: Healthy, Tumor; HOST_SUBJECT_ID	Colorectal carcinoma tumors and adjacent nonaffected tissues	Cancer		172	908	3,228	980	409	Y
Montassier 2016	Treatment: bact, NObact	Patients prior to chemotherapy who did or did not develop bacteremia	Bacteremia		28	541	1,852	640	228	N
Morgan 2012	ULCERATIVE_COLIT_OR_CROHNS_DIS: Crohn's disease, Healthy	Healthy controls or patients with CD or ulcerative colitis	IBD		128	829	3,677	877	367	N
	ULCERATIVE_COLIT_OR_CROHNS_DIS: Ulcerative Colitis, Healthy	Healthy controls or patients with CD or ulcerative colitis	IBD		128	829	3,677	877	367	N
Qin 2012	Diabetic: Y, N	Healthy controls or patients with type 2 diabetes	Diabetes		124	11,880	NA	2,526	NA	N
Qin 2014	Cirrhotic: Cirrhosis, Healthy	Healthy controls or patients with cirrhosis	Cirrhosis		130	8,483	NA	2,579	NA	N
Ravel 2011	Ethnic_Group: Black, Hispanic	Vaginal microbiomes of black and Hispanic women	Vaginal		199	586	1,093	660	305	N
	Nugent_score_category: low, high	Predict Nugent score category (low, high) from vaginal microbiome	Vaginal		342	586	1,093	660	305	N
	Nugent_score	Predict Nugent score from vaginal microbiome	Vaginal	X	388	586	1,093	660	305	N
	pH	Predict pH from vaginal microbiome	Vaginal	X	388	586	1,093	660	305	N
	Ethnic_Group: White, Black	Vaginal microbiomes of white and black women	Vaginal		200	586	1,093	660	305	N
Turnbaugh 2009	OBESITYCAT: Lean, Obese; ZYGOSITY: MZ, DZ, Mom	Lean or obese individuals (monozygotic or dyzygotic twins or their mothers)	Obesity		142	557	4,051	680	232	Y
Wu 2011	DIET: HighFat, LowFat	Individuals after completing a high-fat or low-fat diet intervention	Diet		10	292	1,769	361	136	N
Yatsunenko 2012	AGE	Infants (up to age 3 years) from the USA	Age	X	49	4,660	15,783	4,021	1,544	N
	COUNTRY: GAZ: Venezuela, GAZ: Malawi	Individuals living in Malawi or Venezuela	Geography		54	4,660	15,783	4,021	1,544	N
	SEX: male, female	Males and females from the USA	Sex		129	4,660	15,783	4,021	1,544	N
	COUNTRY: GAZ: United States of America, GAZ: Malawi	Individuals living in the USA or Malawi	Geography		150	4,660	15,783	4,021	1,544	N

Abx: antibiotics; CD: Crohn's disease; DZ: dizygotic; GG: Greengenes; IBD: inflammatory bowel disease; IGT: impaired glucose tolerance; MZ: monozygotic; NGT: normal glucose tolerance; PCDAI: Pediatric Crohn's Disease Activity Index; T2D: type 2 diabetes; GAZ: Gazeteer, an ontology of place names.

### Future work

We expect and hope that the broader microbiome research community will add new datasets and prediction tasks to ML Repo. We provide instructions [30] on our GitHub repository to guide users to create a fork from our repository, add the appropriate data and files, and update the master task and dataset lists. Researchers can then submit a pull request for our review, and requests that are properly formatted will be accepted and merged into the repository. We expect that data submissions will come from either the original researchers or those well acquainted with the datasets, and hence will expect that sample selection and subsetting will have undergone rigorous review for prediction tasks.

## Methods

### Pre-processing of sequencing reads

When available, preprocessed FASTA files were downloaded from QIITA (or previously, the QIIME database). For all other datasets, raw FASTQ files were downloaded from sources listed in Supplemental Table 1. Adaptors and barcodes were removed and sequences were quality filtered (at Phred score ≥ Q20) using SHI7 [12] or QIIME [13]. OTUs were picked from processed FASTA files using BURST [14] with Greengenes [16] 97 or the NCBI RefSeq Targeted Loci Project 16s project [15] (accessed on 4 July 2017). Samples with sequencing depth <1,000 sequences per sample were dropped for all studies, except for 5 datasets [31–35], where the minimum threshold was 100 sequences per sample.

### Selection of classification tasks

Classification tasks were selected on the basis of reported study results, biologically relevant high-level phenotypes, and sufficient sample sizes. Original metadata files and research methods were rigorously and manually curated in order to subset samples with minimal confounders. For confounders that were inherent to the study, we include an additional variable to control for in the task metadata files. The presence of control variables can be found by examining “control_vars” in the Tasks table.

### Website generation

Website templating was developed using Jinja2 [36] and custom Python scripts. Individual webpages were generated by iterating through items in the Tasks and Datasets tables, and dynamically populating templates to generate individual Markdown [37] pages. The resulting Markdown pages are hosted as GitHub Pages.

### Case study benchmarking

Case study results were generated with custom R [38] scripts, which can be found in the /*example* folder in the ML Repo Github repository. To compare machine learning models, we iterated through tasks with binary responses. OTU counts were converted to relative abundances, filtered at a minimum of 10% prevalence across samples, and collapsed at a complete-linkage correlation of 95% (which is done by calculating the Pearson correlation between each pair of OTUs using all complete pairs of observations, hierarchically clustering the results, and cutting the resulting dendrogram at a height of 0.05). We then constructed a 5-fold cross-validation for tasks containing >100 samples, or a leave-one-out cross-validation for tasks with fewer samples. For n-fold cross validation, samples were assigned to folds such that classes were equally balanced within each fold (e.g., if our task contained 40% healthy and 60% diseased samples, our folds would also be selected to represent this distribution). For tasks that contained control variables, we selected folds such that samples with the same control variable value were contained within the same fold. For example, for a task dataset containing matching stool and oral samples from subjects, the Subject Identifier would be listed as the control variable and we should assign samples to folds such that all samples from a specific subject were contained within a fold. This step is crucial to avoid biasing or overfitting the training model; test folds should contain not only new samples but also samples that are independent from those in the training set. Models were constructed using the “caret” package [39]. Control parameters were set using the function trainControl with parameter method = “none” and default parameters. Default settings for all models are as follows: SVM radial basis σ is set to 0.1, all SVMs [40]C is set to 1, and randomForest number of trees is set to 500 and number of variables to split is sqrt(*p*), where *p* is the number of features. This entire process was bootstrapped 100 times, and the mean class probabilities were used to calculate the resulting AUCs and ROCs. To compare classification accuracies using different reference databases, we used a similar procedure but held the model constant and predicted using different base OTU tables. This framework enables comparison of a myriad of machine learning models available in the “caret” package and can be easily expanded to compare different OTU-picking algorithms, or normalization and filtering techniques.

## Availability of supporting data and materials

All test datasets are available in the Microbiome Learning Repo site [17]. Snapshots of our code and other supporting data are available in the *GigaScience* database, GigaDB [41].

## Availability of supporting source code and requirements

Project name: Microbiome Learning Repo

Project home page: https://knights-lab.github.io/MLRepo/

Operating system: platform independent

Programming language: Python, R

License: MIT License

Restrictions: None

RRID:SCR_017079

## Supporting information

**Table:** Glossary

Term	Definition
OTU	Operational taxonomic unit, group of closely related organisms based on DNA sequence similarity
16S	16S ribosomal RNA gene, component of the prokaryotic ribosome, used to reconstruct phylogenies
FASTA	Text-based format for representing nucleotide sequences with single-letter codes
FASTQ	Text-based format for representing nucleotide sequences and corresponding quality scores, with single-letter codes for nucleotides and quality
Taxa	Groups of ≥1 populations of organisms. Usually summarized at phylum, class, order, family, genus, or species levels
Metadata	Descriptive data pertaining to samples within a study
Shotgun	Shotgun metagenomics sequencing breaks up all available DNA into random small segments and uses chain termination to sequence reads. Reads can be aligned directly to a reference database, or overlapping reads can be assembled into contiguous sequences

GIGA-D-18-00316_Original_Submission.pdf

GIGA-D-18-00316_Revision_1.pdf

Response_to_Reviewer_Comments_Original_Submission.pdf

Reviewer_1_Report_Original_Submission -- Pierre Mahe 9/20/2018 Reviewed

Reviewer_2_Report_Original_Submission -- Edoardo Pasolli 9/21/2018 Reviewed

Reviewer_2_Report_Revision_1 -- Edoardo Pasolli 3/5/2019 Reviewed

Reviewer_3_Report_Original_Submission -- Robert Rentzsch, Ph.D. 9/21/2018 Reviewed

Reviewer_3_Report_Revision_1 -- Robert Rentzsch, Ph.D. 2/28/2019 Reviewed

## Abbreviations

AUC: area under the curve; IBD: inflammatory bowel disease; ML Repo: Microbiome Learning Repo; NCBI: National Center for Biotechnology Information; OTU: operational taxonomic unit; ROC: receiver operating curve; SVM: support vector machine.

## Competing interests

D.K. serves as CEO and holds equity in CoreBiome, a company involved in the commercialization of microbiome analysis. The University of Minnesota also has financial interests in CoreBiome under the terms of a license agreement with CoreBiome. These interests have been reviewed and managed by the University of Minnesota in accordance with its Conflict-of-Interest policies.

## Funding

This work is supported by funds from National Institutes of Health grant R01AI121383.

## Authors’ contributions

Conceptualization: P.V. and D.K.; data curation: P.V.; formal analyses: P.V.; methodology: P.V., B.M.H., D.K.; software: P.V.; writing—original draft: P.V.; writing—review and editing: B.M.H. and D.K.
