# Supplementary material for: Microbiome Learning Repo (ML Repo): A public repository of microbiome regression and classification tasks
Source: Gigascience. 2019 Apr 26;8(5):giz042. doi: 10.1093/gigascience/giz042 (PMC6493971; doi:10.1093/gigascience/giz042)
Supplement: GIGA-D-18-00316_Original_Submission.pdf [file giz042_giga-d-18-00316_original_submission.pdf]

GigaScience

Microbiome Learning Repo (ML Repo): A public repository of microbiome regression and classification tasks

--Manuscript Draft--

**Manuscript Number:**	GIGA-D-18-00316	
**Full Title:**	Microbiome Learning Repo (ML Repo): A public repository of microbiome regression and classification tasks	
**Article Type:**	Technical Note	
**Funding Information:**	National Institute of Allergy and Infectious Diseases (R01AI121383)	Not applicable
**Abstract:**	The use of machine learning in high-dimensional biological applications, such as the human microbiome, has grown exponentially in recent years. Unfortunately, challenges still exists for machine learning algorithm developers who often lack domain expertise required for interpretation and curation of the heterogeneous microbiome datasets. We present Microbiome Learning Repo (ML Repo), a public, web-based repository of 33 curated classification and regression tasks from 15 published human microbiome datasets. We highlight the use of ML Repo in several use cases to demonstrate its wide application, and expect it to be an important resource for algorithm developers.	
**Corresponding Author:**	Dan Knights<br><br>UNITED STATES	
**Corresponding Author Secondary Information:**		
**Corresponding Author's Institution:**		
**Corresponding Author's Secondary Institution:**		
**First Author:**	Pajau Vangay, Ph.D.	
**First Author Secondary Information:**		
**Order of Authors:**	Pajau Vangay, Ph.D.	
	Dan Knights, Ph.D.	
	Benjamin M. Hillmann	
**Order of Authors Secondary Information:**		
**Additional Information:**		
**Question**	**Response**	
Are you submitting this manuscript to a special series or article collection?	No	
**Experimental design and statistics**<br><br>Full details of the experimental design and statistical methods used should be given in the Methods section, as detailed in our Minimum Standards Reporting Checklist. Information essential to interpreting the data presented should be made available in the figure legends.	Yes	

Powered by Editorial Manager® and ProduXion Manager® from Aries Systems Corporation

Have you included all the information requested in your manuscript?	
**Resources**<br><br>A description of all resources used, including antibodies, cell lines, animals and software tools, with enough information to allow them to be uniquely identified, should be included in the Methods section. Authors are strongly encouraged to cite Research Resource Identifiers (RRIDs) for antibodies, model organisms and tools, where possible.<br><br>Have you included the information requested as detailed in our Minimum Standards Reporting Checklist?	Yes
**Availability of data and materials**<br><br>All datasets and code on which the conclusions of the paper rely must be either included in your submission or deposited in publicly available repositories (where available and ethically appropriate), referencing such data using a unique identifier in the references and in the “Availability of Data and Materials” section of your manuscript.<br><br>Have you have met the above requirement as detailed in our Minimum Standards Reporting Checklist?	Yes

Click here to download Manuscript MLRepo Manuscript.pdf

Microbiome Learning Repo (ML Repo): A public repository of microbiome regression and classification tasks

Pajau Vangay[1], Benjamin M. Hillmann[2], Dan Knights[12]*

[1]Bioinformatics and Computational Biology, University of Minnesota, Minneapolis, MN, 55455, vanga015@umn.edu

[2]Department of Computer Science and Engineering, University of Minnesota, Minneapolis, MN, 55455, hillm096@umn.edu

*Corresponding author: dknights@umn.edu

Abstract

The use of machine learning in high-dimensional biological applications, such as the human microbiome, has grown exponentially in recent years. Unfortunately, challenges still exists for machine learning algorithm developers who often lack domain expertise required for interpretation and curation of the heterogeneous microbiome datasets. We present Microbiome Learning Repo (ML Repo), a public, web-based repository of 33 curated classification and regression tasks from 15 published human microbiome datasets. We highlight the use of ML Repo in several use cases to demonstrate its wide application, and expect it to be an important resource for algorithm developers.

Keywords

Microbiome, machine learning, repository, database

Findings

Background

Machine learning is widely used as a method for classification and prediction, with a growing number of applications in human health [1]. The use of machine learning in biological fields [2,3], and more specifically the microbiome research field [4–7], has grown exponentially due to the robustness of these algorithms to high dimensional data. However, challenges exist for large-scale meta-analysis as they often require manual curation of metadata and standardized processing of raw sequence data, resulting in variation in the results derived from chosen datasets across studies [8,9]. In addition, microbiome research data can be challenging to access and analyze for expert machine learning algorithm developers, who often do not have the domain expertise required to parse the data and metadata in complex microbiome studies. There exist general resources with curated classification tasks from variety of domains. The University of California Irvine (UCI) Machine Learning Repository [10] revolutionized machine learning methods development by giving developers access to many curated datasets; its widespread usage and impact can be seen from its thousands of resulting citations. Currently, we are unaware of any machine learning repository specifically for microbiome classification tasks. We constructed a complementary database to address this deficiency, in order to promote the development of and usage of improved machine learning methods for the microbiome community.

Workflow

We present the Microbiome Learning Repo (ML Repo), a repository of 33 curated classification and regression tasks using human microbiome data. Our 33 tasks are curated from 15 publicly available human microbiome datasets, which include 12 amplicon-based and 3 shotgun sequencing datasets [Table 1]. These datasets vary across sequencing technology platforms,

16s hypervariable regions, and study design, in order to help developer ensure robustness of algorithms across data types. We streamlined the microbiome data using a single post-processing workflow [Fig 1A]. We downloaded trimmed and quality filtered sequencing reads for n=8 datasets from QIITA [11], and raw sequences for n=7 datasets from public repositories. We preprocessed raw sequences using SHI7 [12] or QIIME [13] according to individual technologies and characteristics of each study. Full details regarding the data preprocessing are provided for each data set in the repository. We picked Operational Taxonomic Units (OTUs) from all quality filtered sequences using a closed-reference method with the BURST [14] aligner against both the NCBI RefSeq 16S ribosomal RNA project [15] and the Greengenes 97 database [16]. Samples with depths lower than 1000 sequences per sample were dropped for n=10 datasets, while we applied a lower threshold of 100 sequences per sample for n=5 datasets which had lower expected bacterial load. As a result, for each dataset we generated RefSeq-based OTU and taxa abundance counts, and Greengenes-based OTU and taxa abundance counts. We excluded additional post-processing filtering and normalization steps so that these parameters can be included in future benchmarking use cases as needed. We also limit our data to OTU and taxa tables as other metrics such as alpha and beta diversity can be subsequently generated as needed.

Sample metadata from individual studies were manually curated to generate viable prediction tasks. When available, published study exclusion criteria was applied accordingly and confounders were removed by dropping samples or stratification. Studies that were cross-sectional by design but contained several samples per subject were filtered to contain one sample per subject. Well-known confounders, such as geography, were accounted for when constructing prediction tasks for other human-associated conditions. Longitudinal studies were reduced to single time points of interest to minimize the effect of high intra-individual similarities. Hence, each prediction task is made available as an individual, compartmentalized metadata file

that contains sample identifiers, responses to predict, and optionally, confounder variables to control for. As a result, we generated 33 distinct tasks for predicting human-associated responses.

Publicly available web-based interface

We expect two types of users: (1) machine-learning algorithm developers with limited knowledge of microbiome study designs and (2) microbiome researchers interested in obtaining additional datasets for meta-analysis. Generally, we expect that methods developers will be most interested in sweeping through the full set of prediction tasks for benchmarking, and hence would prefer to download a single compressed file containing all tasks and data. On the other hand, we expect that microbiome researchers will be more selective in downloading specific datasets and tasks depending on their research domain. Hence, researchers may prefer to browse specific details about tasks and datasets prior to downloading.

Based on these expected use cases, we created a publicly available web-interface for MLRepo hosted by GitHub Pages and available at: https://knights-lab.github.io/MLRepo. Tasks are organized by relevant response categories [Fig 2A]. Task pages contain descriptive details such as Sample Size and Response Type that are specific to the selected prediction task, as well as links for downloading OTU tables, taxa tables, and sample metadata [Fig 2B]. Dataset pages contain important details about the entire dataset, including links to the original research study, as well as original metadata files and quality filtered sequences [Fig 2C]. We also provide a single compressed file containing the entire set of available tasks (OTU tables, taxa tables, and relevant metadata) for download from the main home page.

Benefits of curated microbiome-based prediction tasks

We expect MLRepo to be beneficial for both the machine-learning community as well as the microbiome research community. MLRepo will be a powerful complement to UCI's machine learning repository, as it will allow for benchmarking curated classification tasks with high-dimensional data, and hence enable the subsequent development of novel algorithms for these complex datasets. Our streamlined approach in generating OTU and taxa tables offers a rich set of 15 datasets that microbiome researchers can use directly for further comparison with their own studies, for teaching and learning purposes, or for large meta-analyses. We expect that our provided OTU and taxa tables will also be beneficial for researchers with limited access to high-performance computing resources or bioinformatics skills necessary for processing raw sequencing data. In addition, we expect microbiome-specific methods development will also benefit from our repository prediction tasks. The subsetted samples found in each prediction task metadata file replaces the work of rigorously deciphering metadata and nuances from individual research studies. Hence, new methods, such as OTU-picking algorithms, can be evaluated not only on metrics such as speed and accuracy, but also based on overall impact to study findings.

Comparison to similar databases

Although a number of microbiome repositories exist, many are intended as data archival repositories [17,18] or function as resources for aggregating across studies [19]. Resources such as QIITA [11] offer an extensive collection of datasets, and mock-community-based Mockrobiota [20] is well-suited for benchmarking upstream methods, but neither offer support for the metadata interpretation necessary for predicting high-level phenotypes. MLRepo differs from all of these resources in that we provide well-defined tasks for predicting responses from manually curated metadata and standardized data from published microbiome research studies.

Case studies

We compare the performance of three machine learning models: a random forest [21], and a support vector machine [22] (SVM) with either a radial or linear kernel. Sweeping through available tasks with binary responses, we compare our models by examining receiver operating curves (ROCs) and areas under the curve (AUC) [Fig 3]. Through comparison of ROCs, we can see that random forest outperforms or ties the other two models in 21 out of the 28 tasks. The choice of kernels for SVM appears to have limited impact on overall mean accuracy, yet a linear kernel can perfectly classify penicillin-treated and vancomycin-treated mouse cecal contents when the other models could not; further examination of the microbial features in these samples may be warranted to better understand the strengths of this kernel. We also performed pairwise comparisons of random forest against the other models across all tasks. When evaluated by AUC, considered the standard method for machine learning model evaluation [23,24], random forest performs significantly better than both SVM with a linear kernel (P=0.0014) and with a radial kernel (P=0.00032) [Fig 4A]. We found that random forest accuracy improvements were moderate when compared with SVM-Linear (P=0.083) and SVM-Radial (P=0.03) [Fig 4B]. Our results support the broad usage [4,5,8,25] and acceptance of random forest as a robust classifier [6] with high-dimensional microbiome data.

To assess the impact of reference database choice on classification accuracies, we also used the classification tasks to compare random forest using OTUs picked with the Greengenes 97 database or the NCBI RefSeq Targeted Loci Project 16s project. We find that there is limited impact of database choice to overall classification accuracies [Fig 4C, Fig 5]. This may be due to (1) large effect sizes that are driven mainly by several well-characterized bacterial taxa present in both databases (e.g. stool versus tongue samples), or (2) small effect sizes such that classification is difficult regardless of the database (e.g. male versus female stool).

Future work

We expect and hope that the broader microbiome research community will add new datasets and prediction tasks to MLRepo. We provide instructions on our GitHub repository to guide users to create a fork from our repository, add the appropriate data and files, and update the master task and dataset lists. Researchers can then submit a pull request for our review, and if properly formatted, will be accepted and merged into the repository. We expect that data submissions will come from either the original researchers or those well-acquainted with the datasets, and hence will expect that sample selection and subsetting will have undergone rigorous review for prediction tasks.

Methods

Pre-processing of sequencing reads

When available, preprocessed FASTA files were downloaded from QIITA (or previously, the QIIME database). For all other datasets, raw FASTQ files were downloaded from sources listed in Supplemental Table 1. Sequences were trimmed and quality filtered using SHI7 [12] or QIIME [13]. OTUs were picked from processed FASTA files using BURST [26] with Greengenes [16] 97 or the NCBI RefSeq Targeted Loci Project 16s project [15] (accessed on 17-07-04). Samples with sequencing depth lower than 1000 sequences per sample were dropped for all studies, except for five datasets [27–31], where the minimum threshold was 100 sequences per sample.

Selection of classification tasks

Classification tasks were selected based on reported study results, biologically relevant high-level phenotypes, and sufficient sample sizes. Original metadata files and research methods were rigorously and manually curated in order to subset samples with minimal confounders. For confounders that were inherent to the study, we include an additional variable to control for in

the task metadata files. Presence of control variables can be found by examining "control_vars" in the Tasks table.

Website generation

Website templating was developed using Jinja2 [32] and custom Python scripts. Individual webpages were generated by iterating through items in the Tasks and Datasets tables, and dynamically populating templates in order to generate individual Markdown [33] pages. The resulting Markdown pages are hosted as GitHub Pages.

Case Study Benchmarking

Case study results were generated with custom R [34] scripts, which can be found in the */example* folder in the MLRepo Github repository. To compare machine learning models, we iterated through tasks with binary responses. OTU counts were converted to relative abundances, filtered at a minimum of 10% prevalence across samples, and collapsed at a complete-linkage correlation of 95%. We then constructed a 5-fold cross-validation for tasks containing more than 100 samples, or a leave-one-out cross-validation for tasks with smaller sample sizes. For n-fold cross validation, samples were assigned to folds such that classes were equally balanced within each fold (e.g. if our task contained 40% healthy and 60% diseased samples, our folds would also be selected to represent this distribution). For tasks that contained control variables, we selected folds such that samples with the same control variable value were contained within the same fold. For example, for a task dataset containing matching stool and oral samples from subjects, the Subject Identifier would be listed as the control variable and we should assign samples to folds such that all samples from a specific subject were contained within a fold. This step is crucial to avoid biasing or overfitting the training model; test folds should contain not only new samples, but also samples that are independent from those in the training set. Models were constructed using the 'caret' package [35]. This

process was bootstrapped 100 times, and the mean class probabilities were used to calculate the resulting AUCs and ROCs. To compare classification accuracies using different reference databases, we used a similar procedure but held the model constant and predicted using different base OTU tables. This framework enables comparison of a myriad of machine learning models available in the 'caret' package, and can be easily expanded to compare different OTU-picking algorithms, or normalization and filtering techniques.

Availability of supporting source code and requirements

Project name: Microbiome Learning Repo
Project home page: https://knights-lab.github.io/MLRepo/
Operating system: Platform independent
Programming language: Python, R
License: MIT License

Declarations

Authors' contributions

Conceptualization: P.V. and D.K; Data curation: P.V.; Formal analyses: P.V.; Methodology: P.V., B.H., D.K.; Software: P.V.; Writing - original draft: P.V.; Writing - review and editing: B.H. and D.K.

Competing Interests

D.K. serves as CEO and holds equity in CoreBiome, a company involved in the commercialization of microbiome analysis. The University of Minnesota also has financial interests in CoreBiome under the terms of a license agreement with CoreBiome. These interests

have been reviewed and managed by the University of Minnesota in accordance with its Conflict-of-Interest policies.

References

1. Jordan MI, Mitchell TM. Machine learning: Trends, perspectives, and prospects. Science. 2015;349:255–60.

2. Shipp MA, Ross KN, Tamayo P, Weng AP, Kutok JL, Aguiar RCT, et al. Diffuse large B-cell lymphoma outcome prediction by gene-expression profiling and supervised machine learning. Nat Med. 2002;8:68–74.

3. Furey TS, Cristianini N, Duffy N, Bednarski DW, Schummer M, Haussler D. Support vector machine classification and validation of cancer tissue samples using microarray expression data. Bioinformatics. 2000;16:906–14.

4. Aagaard K, Riehle K, Ma J, Segata N, Mistretta T-A, Coarfa C, et al. A metagenomic approach to characterization of the vaginal microbiome signature in pregnancy. PLoS One. 2012;7:e36466.

5. Yatsunenko T, Rey FE, Manary MJ, Trehan I, Dominguez-Bello MG, Contreras M, et al. Human gut microbiome viewed across age and geography. Nature. 2012;486:222–7.

6. Knights D, Costello EK, Knight R. Supervised classification of human microbiota. FEMS Microbiol Rev. 2011;35:343–59.

7. Smith MI, Yatsunenko T, Manary MJ, Trehan I, Mkakosya R, Cheng J, et al. Gut microbiomes of Malawian twin pairs discordant for kwashiorkor. Science. 2013;339:548–54.

8. Pasolli E, Truong DT, Malik F, Waldron L, Segata N. Machine Learning Meta-analysis of

Large Metagenomic Datasets: Tools and Biological Insights. PLoS Comput Biol. Public Library of Science; 2016;12:e1004977.

9. Sze MA, Schloss PD. Looking for a Signal in the Noise: Revisiting Obesity and the Microbiome. MBio [Internet]. 2016;7. Available from: http://dx.doi.org/10.1128/mBio.01018-16

10. Asuncion A, Newman D. UCI machine learning repository [Internet]. 2007. Available from: https://ergodicity.net/2013/07/

11. Qiita Development Team. QIITA [Internet]. Available from: http://qiita.microbio.me/

12. Al-Ghalith GA, Hillmann B, Ang K, Shields-Cutler R, Knights D. SHI7 Is a Self-Learning Pipeline for Multipurpose Short-Read DNA Quality Control. mSystems [Internet]. 2018;3. Available from: http://dx.doi.org/10.1128/mSystems.00202-17

13. Caporaso JG, Kuczynski J, Stombaugh J, Bittinger K, Bushman FD, Costello EK, et al. QIIME allows analysis of high-throughput community sequencing data. Nat Methods. 2010;7:335–6.

14. Al-Ghalith G, Knights D. BURST enables optimal exhaustive DNA alignment for big data [Internet]. Zenodo; 2017. Available from: http://dx.doi.org/10.5281/ZENODO.806850

15. O'Leary NA, Wright MW, Brister JR, Ciufo S, Haddad D, McVeigh R, et al. Reference sequence (RefSeq) database at NCBI: current status, taxonomic expansion, and functional annotation. Nucleic Acids Res. 2016;44:D733–45.

16. McDonald D, Price MN, Goodrich J, Nawrocki EP, DeSantis TZ, Probst A, et al. An improved Greengenes taxonomy with explicit ranks for ecological and evolutionary analyses of bacteria and archaea. ISME J. 2012;6:610–8.

17. Hunter S, Corbett M, Denise H, Fraser M, Gonzalez-Beltran A, Hunter C, et al. EBI

metagenomics—a new resource for the analysis and archiving of metagenomic data. Nucleic Acids Res. Oxford University Press; 2014;42:D600–6.

18. Leinonen R, Sugawara H, Shumway M, International Nucleotide Sequence Database Collaboration. The sequence read archive. Nucleic Acids Res. 2011;39:D19–21.

19. Forster SC, Browne HP, Kumar N, Hunt M, Denise H, Mitchell A, et al. HPMCD: the database of human microbial communities from metagenomic datasets and microbial reference genomes. Nucleic Acids Res. 2016;44:D604–9.

20. Bokulich NA, Rideout JR, Mercurio WG, Shiffer A, Wolfe B, Maurice CF, et al. mockrobiota: a Public Resource for Microbiome Bioinformatics Benchmarking. mSystems [Internet]. 2016;1. Available from: http://dx.doi.org/10.1128/mSystems.00062-16

21. Breiman L. Random Forests. Mach Learn. 2001;45:5–32.

22. Cortes C, Vapnik V. Support-vector networks. Mach Learn. 1995;20:273–97.

23. Huang J, Ling CX. Using AUC and accuracy in evaluating learning algorithms. IEEE Trans Knowl Data Eng. 2005;17:299–310.

24. Ling CX, Huang J, Zhang H, Others. AUC: a statistically consistent and more discriminating measure than accuracy. IJCAI. 2003. p. 519–24.

25. Karlsson FH, Tremaroli V, Nookaew I, Bergström G, Behre CJ, Fagerberg B, et al. Gut metagenome in European women with normal, impaired and diabetic glucose control. Nature. 2013;498:99–103.

26. Al-Ghalith G, Knights D. BURST enables optimal exhaustive DNA alignment for big data [Internet]. Zenodo; 2017. Available from: http://dx.doi.org/10.5281/ZENODO.806850

27. Gevers D, Kugathasan S, Denson LA, Vázquez-Baeza Y, Van Treuren W, Ren B, et al. The treatment-naive microbiome in new-onset Crohn's disease. Cell Host Microbe. 2014;15:382–92.

28. Human Microbiome Project Consortium. A framework for human microbiome research. Nature. 2012;486:215–21.

29. Kostic AD, Gevers D, Pedamallu CS, Michaud M, Duke F, Earl AM, et al. Genomic analysis identifies association of Fusobacterium with colorectal carcinoma. Genome Res. 2012;22:292–8.

30. David LA, Maurice CF, Carmody RN, Gootenberg DB, Button JE, Wolfe BE, et al. Diet rapidly and reproducibly alters the human gut microbiome. Nature. 2014;505:559–63.

31. Turnbaugh PJ, Hamady M, Yatsunenko T, Cantarel BL, Duncan A, Ley RE, et al. A core gut microbiome in obese and lean twins. Nature. 2009;457:480–4.

32. Ronacher A. Jinja2 [Internet]. 2017. Available from: http://jinja.pocoo.org/

33. Gruber J, Swartz A, Others. Markdown. 2004.

34. Team RC, Others. R: A language and environment for statistical computing. Citeseer; 2013; Available from: http://citeseerx.ist.psu.edu/viewdoc/download?doi=10.1.1.470.5851&rep=rep1&type=pdf

35. Kuhn M, Others. Caret package. J Stat Softw. 2008;28:1–26.

Tables

Table 1. Microbiome datasets with available classification tasks in ML Repo.

Project Name	V Region	Target size	Num samples	Num subjects	Area	Description	Sequencing Technology	Study Design
Cho 2012	V3	177	95	47	Antibiotics	Mouse fecal and cecal samples, Control vs. 4 kinds of antibiotics	454	Cross-Sectional
Claesson 2012	V4	221	168	168	Age	Elderly and young adults	454	Cross-Sectional
David 2014	V4	282	235	11	Diet	Plant-based vs. Animal-based diet, Cross-over study	Illumina MiSeq	Longitudinal
Gevers 2014	V4	173	1321	668	IBD	Biopsies from IBD patients prior to treatment	Illumina MiSeq	Cross-Sectional
HMP 2012	V35	527	6407	242	Body Habitat, Gender	Up to 18 body sites across 242 healthy subjects at 1-2 time points	454	Cross-Sectional
Kostic 2012	V35	569	190	95	Colorectal Cancer	Adjacent Healthy vs. Tumor Colon Biopsy Tissues	454	Paired
Montassier 2016	V56	280	28	28	Bacteremia	Patients prior to chemotherapy who did or did not develop bacteremia	454	Cross-Sectional
Morgan 2012	V35	569	231	231	IBD	Healthy, Crohn's Disease, or Ulcerative Colitis patients	454	Cross-Sectional
Turnbaugh 2009	V2	230	281	154	Obesity	Monozygotic or dizygotic twin pairs concordant for BMI class, and their mothers	454	Cross-Sectional
Wu 2011	V12	244	95	10	Diet	Controlled HighFat or LowFat feeding on 10 subjects over 10 days	454	Longitudinal
Yatsunenko 2012	V4	282	531	531	Geography, Age, Gender	Humans of varying ages from the USA, Malawi, and Venezuela	Illumina MiSeq	Cross-Sectional
Ravel 2011	V12	240	396	396	Bacterial Vaginosis	Vaginal samples from four ethnic groups nugent scores for bacterial vaginosis	454	Cross-Sectional
Karlsson 2013	NA	NA	144	144	Diabetes	Patients with normal, impaired, or type 2 diabetes glucose tolerance categories	Illumina HiSeq	Cross-Sectional
Qin 2012	NA	NA	134	134	Diabetes	Healthy vs type 2 diabetes Chinese patients	Illumina HiSeq	Cross-Sectional
Qin 2014	NA	NA	130	130	Cirrhosis	Cirrhosis versus healthy	Illumina HiSeq	Cross-Sectional

ML Repo contains 33 classification and regression tasks from 15 publicly available human microbiome datasets shown here.

Figure Legends

Figure 1. Data processing workflow and website generation.

(A) Quality-filtered sequences were obtained from either the QIITA or from another public repository and trimmed and filtered using SHI7. Reference-based OTUs were picked using BURST with the NCBI RefSeq and Greengenes 97 databases.

(B) Individual GitHub Markdown pages were generated from dataset and task lists with a custom Python script and Jinja2 template, then uploaded to GitHub to be hosted.

Figure 2. Screenshots of ML Repo web interface.

(A) Available classification and regression tasks are listed by high level phenotype categories for browsing.

(B) Individual task webpages contain links to files for classifying a specific task, as well as relevant task-specific metadata.

(C) Individual dataset webpages contain relevant metadata pertaining to the entire dataset, as well as links to raw metadata files and sequencing data.

Figure 3. ROCs comparing random forest and SVM with different kernels.

Sweeping across all binary classification tasks available in MLRepo (n=28), we compare ROCs of random forest, SVM with a radial kernel, and SVM with a linear kernel. AUCs are listed within plots and are colored respective to each model.

Figure 4. Summary statistics of framework and database comparisons.

(A) AUCs random forest (rf) to SVM-Linear (left) and random forest to SVM-Radial (right). Paired t-tests reveal that random forest results in significantly higher AUC than both SVM-Linear (P=0.0014) and SVM-Radial (P=0.00032).

(B) Accuracies of random forest to SVM-Linear (left) and random forest to SVM-Radial (right). Paired t-tests reveal that random forest results in significantly better accuracy than SVM-Radial (P=0.03), but not SVM-Linear (P=0.083).

(C) AUCs (left) and accuracies (right) of random forest classifications of n=24 tasks using OTUs picked with NCBI RefSeq database or Greengenes database as predictors. Student's t-test reveals that reference database choice has limited impact on classification AUC or accuracy. Lines are colored by the top model for each classification task.

Figure 5. ROCs comparing NCBI RefSeq and Greengenes 97 databases. Sweeping across 16s-based binary classification tasks available in MLRepo (n=24), we compare ROCs of random forest with genus-level taxonomic summaries as predictors from OTU-picking strategies with the NCBI RefSeq prokaryote reference database and the Greengenes 97 reference database. AUCs are listed within plots and are colored respective to each database.

Figure 1 Click here to download Figure MLRepo Figure 1.pdf

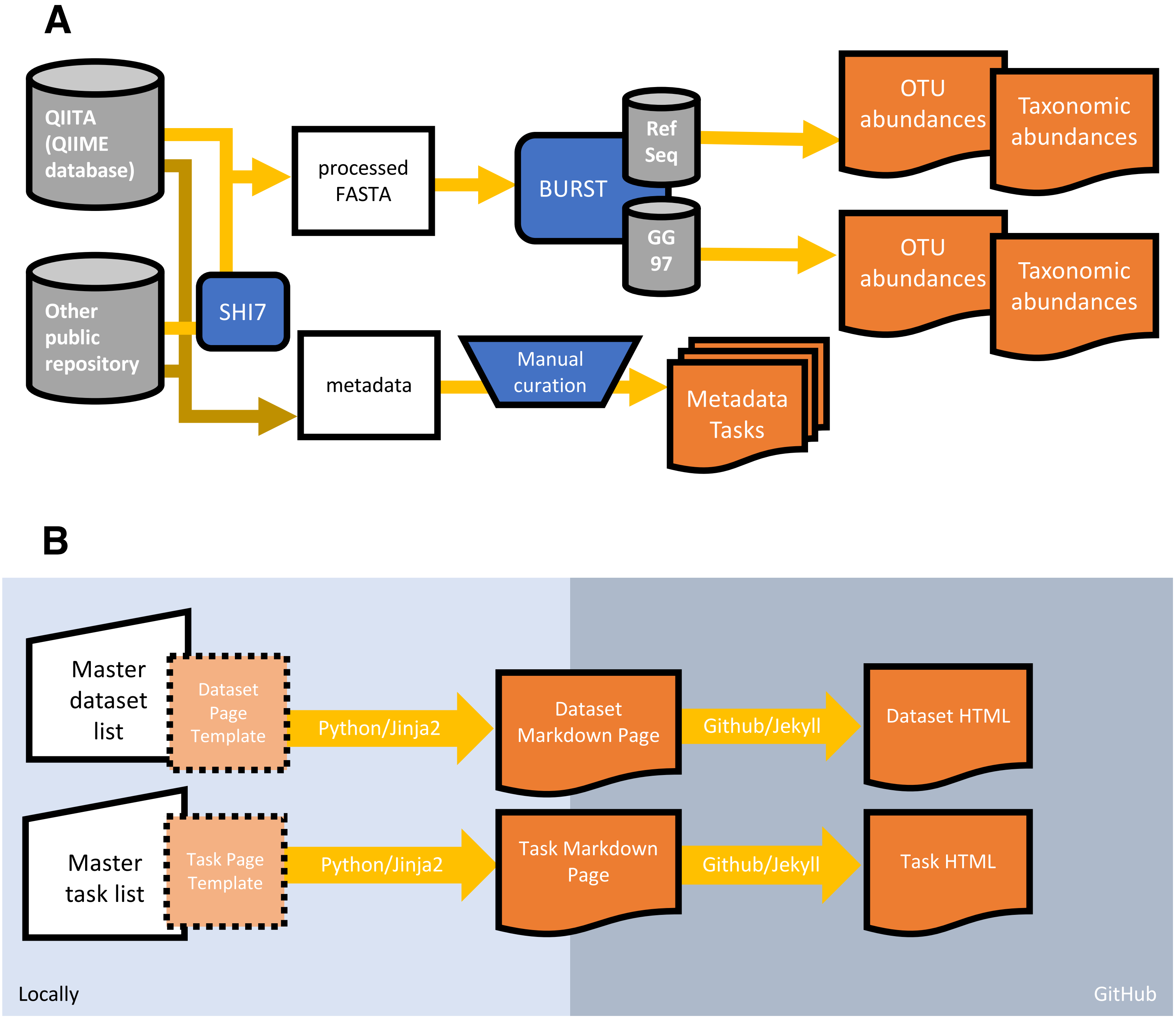

Figure 2 Click here to download Figure MLRepo Figure 2.pdf

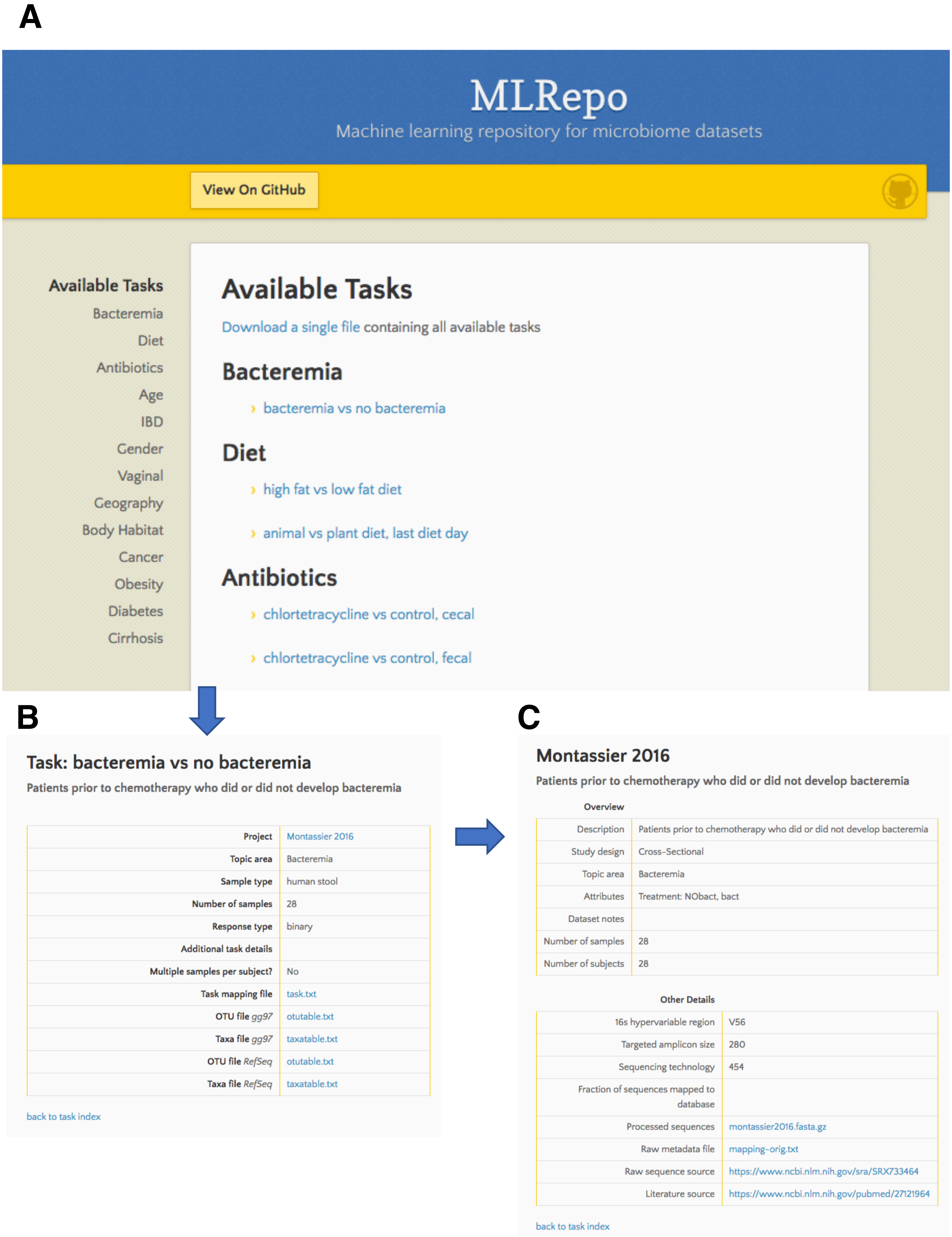

Click here to download Figure MLRepo Figure 3.pdf

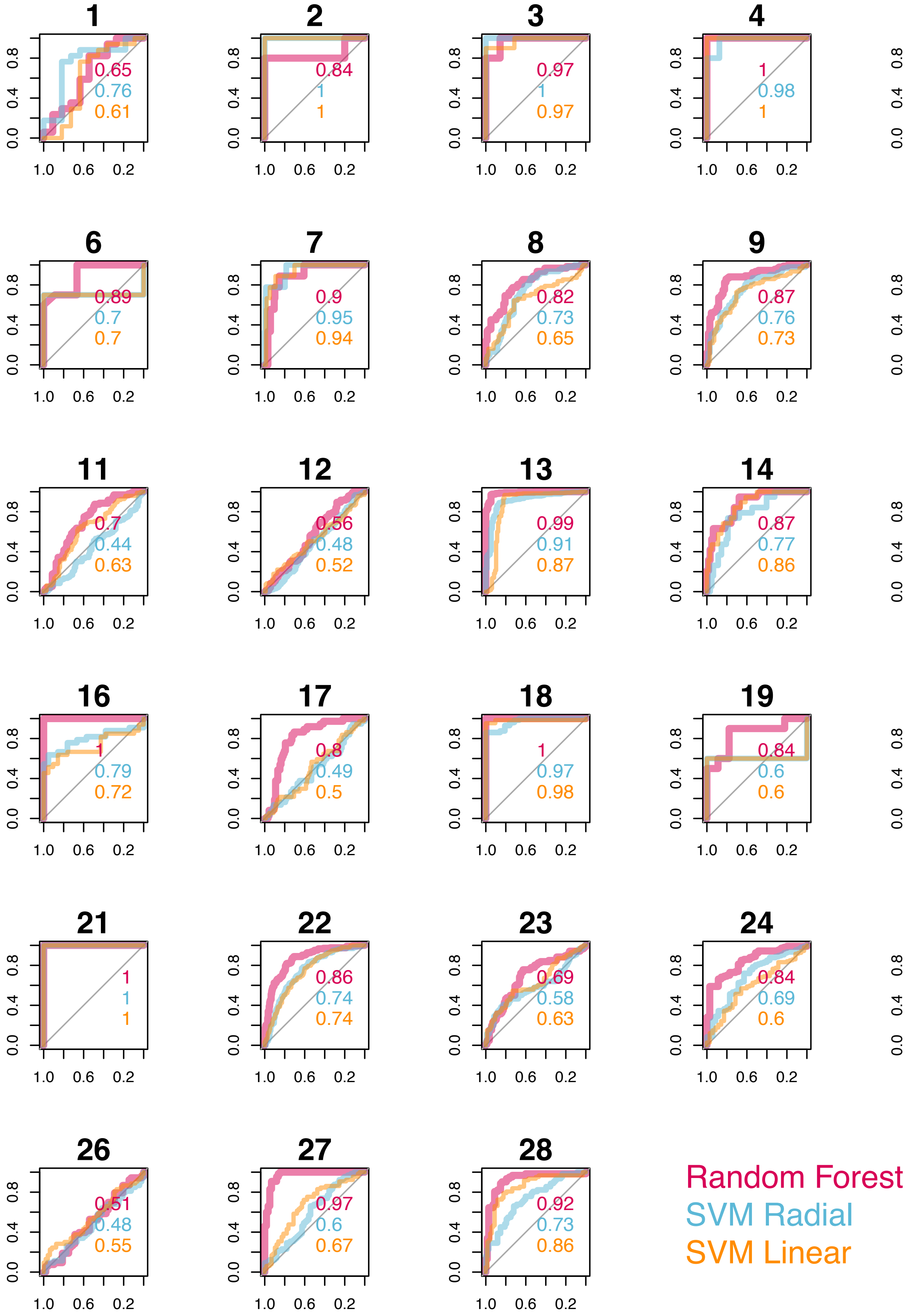

1 bacteremia vs no bacteremia
2 high fat vs low fat diet
3 chlortetracycline vs control, cecal
4 chlortetracycline vs control, fecal
5 penicillin vs vancomycin, cecal
6 penicillin vs vancomycin, fecal
7 elderly vs young
8 control vs cd, ileum
9 control vs cd, rectum
10 male vs female, stool
11 white vs black, vaginal
12 black vs hispanic, vaginal
13 low vs high nugent category
14 healthy vs cd, stool
15 healthy vs uc, stool
16 malawi vs venezuela, adults only
17 male vs female, usa
18 us vs malawi, adults only
19 animal vs plant diet, last diet day
20 gastrointestinal vs oral
21 stool vs tongue
22 subgingival vs supragingival plaque
23 healthy vs tumor biopsy, paired
24 lean vs obese, mz/dz/mom
25 normal vs diabetes glucose tolerance
26 impaired vs diabetes glucose tolerance
27 healthy vs type 2 diabetes
28 healthy vs cirrhosis

Figure 4

Click here to download Figure MLRepo Figure 4.pdf

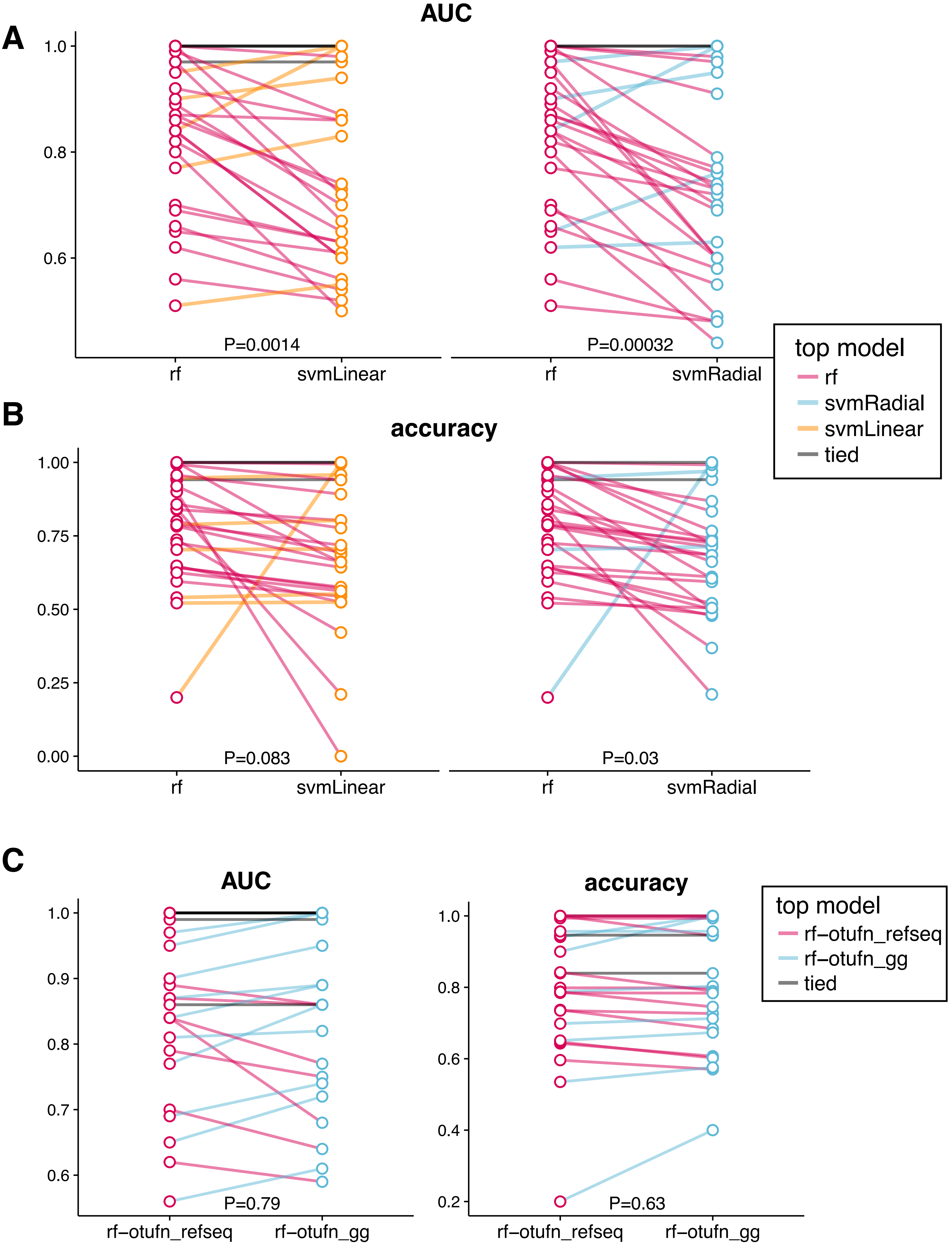

Figure 5

Click here to download Figure MLRepo Figure 5.pdf

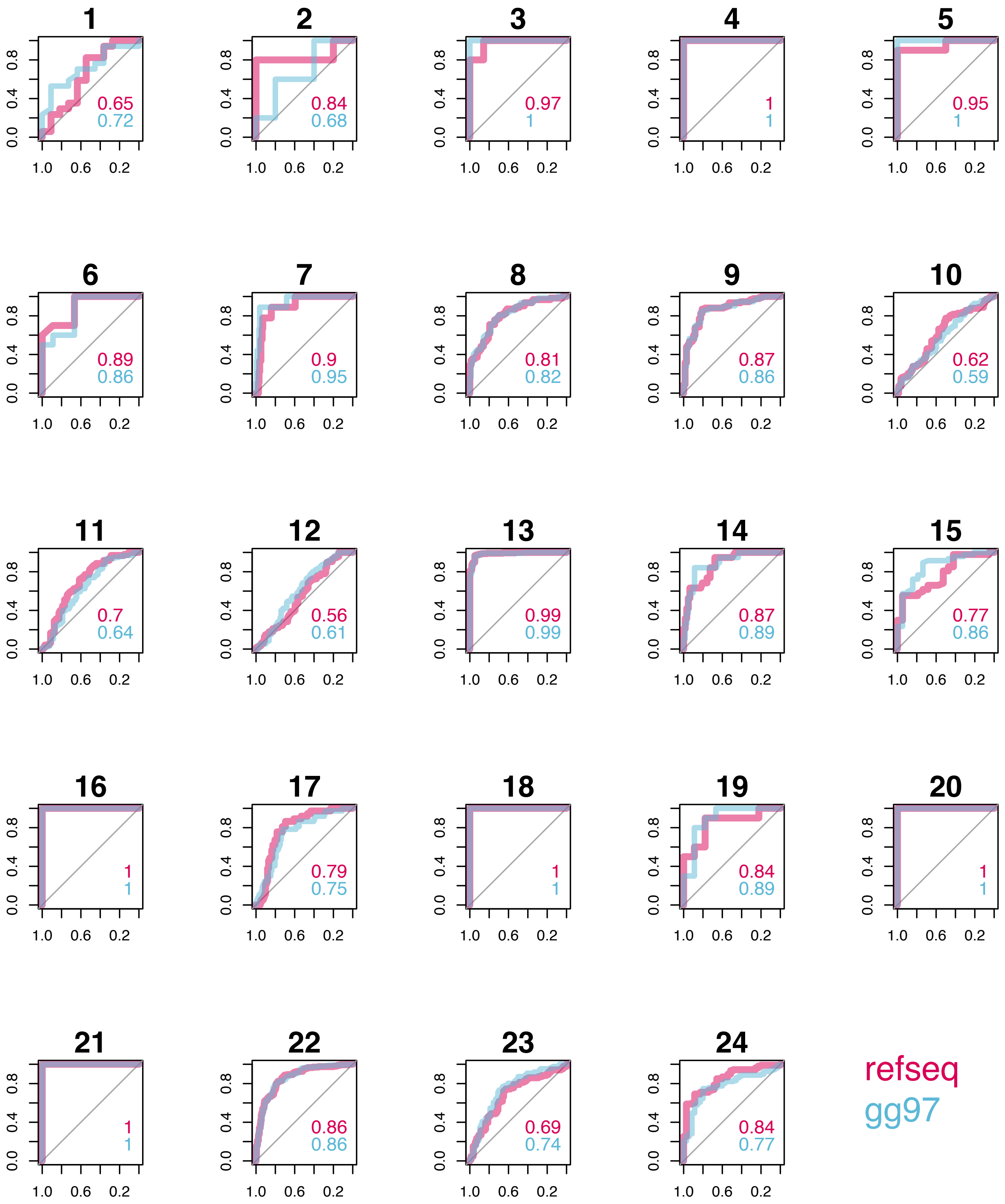

1 bacteremia vs no bacteremia
2 high fat vs low fat diet
3 chlortetracycline vs control, cecal
4 chlortetracycline vs control, fecal
5 penicillin vs vancomycin, cecal
6 penicillin vs vancomycin, fecal
7 elderly vs young
8 control vs cd, ileum
9 control vs cd, rectum
10 male vs female, stool
11 white vs black, vaginal
12 black vs hispanic, vaginal
13 low vs high nugent category
14 healthy vs cd, stool
15 healthy vs uc, stool
16 malawi vs venezuela, adults only
17 male vs female, usa
18 us vs malawi, adults only
19 animal vs plant diet, last diet day
20 gastrointestinal vs oral
21 stool vs tongue
22 subgingival vs supragingival plaque
23 healthy vs tumor biopsy, paired
24 lean vs obese, mz/dz/mom
