# Supplementary material for: Microbiome Learning Repo (ML Repo): A public repository of microbiome regression and classification tasks
Source: Gigascience. 2019 Apr 26;8(5):giz042. doi: 10.1093/gigascience/giz042 (PMC6493971; doi:10.1093/gigascience/giz042)
Supplement: GIGA-D-18-00316_Revision_1.pdf [file giz042_giga-d-18-00316_revision_1.pdf]

GigaScience

Microbiome Learning Repo (ML Repo): A public repository of microbiome regression and classification tasks

--Manuscript Draft--

**Manuscript Number:**	GIGA-D-18-00316R1
**Full Title:**	Microbiome Learning Repo (ML Repo): A public repository of microbiome regression and classification tasks
**Article Type:**	Technical Note
**Funding Information:**	National Institute of Allergy and Infectious Diseases (R01AI121383) — Not applicable
**Abstract:**	The use of machine learning in high-dimensional biological applications, such as the human microbiome, has grown exponentially in recent years, but algorithm developers often lack domain expertise required for interpretation and curation of the heterogeneous microbiome datasets. We present Microbiome Learning Repo (ML Repo, available at https://knights-lab.github.io/MLRepo/), a public, web-based repository of 33 curated classification and regression tasks from 15 published human microbiome datasets. We highlight the use of ML Repo in several use cases to demonstrate its wide application, and expect it to be an important resource for algorithm developers.
**Corresponding Author:**	Dan Knights<br><br>UNITED STATES
**Corresponding Author Secondary Information:**	
**Corresponding Author's Institution:**	
**Corresponding Author's Secondary Institution:**	
**First Author:**	Pajau Vangay, Ph.D.
**First Author Secondary Information:**	
**Order of Authors:**	Pajau Vangay, Ph.D.
	Benjamin M. Hillmann
	Dan Knights, Ph.D.
**Order of Authors Secondary Information:**	
**Response to Reviewers:**	Response to Reviewers<br><br>Reviewer #1:<br>This paper describes MLRepo, a database of standardized microbiome datasets to develop and evaluate machine learning algorithms. The strength of this resource is to provide machine learning researchers a panel of diverse datasets (e.g., regression and classification tasks of various levels of complexity and with a various number of samples), already curated and formatted, to evaluate in an objective way novel algorithms dedicated to the analysis of microbiome samples. It can also be useful for teaching purposes (e.g., for practical lab sessions or to set up "data challenges") and will obviously be valuable to the community of microbiome researchers to set-up meta-analyses.<br><br>Overall I am very enthusiastic about this repository. As a machine-learning method developer interested in microbiome / metagenomics applications, I am indeed well aware that building curated databases and setting up baselines to evaluate novel algorithms is very time consuming, and that results reported in published papers are

Powered by Editorial Manager® and ProduXion Manager® from Aries Systems Corporation

sometimes hard to reproduce. I am therefore convinced that this repository will simplify the whole process and facilitate evaluating algorithms in an objective way. I am therefore very favorable in having this work published in Giga Science.

We thank Reviewer 1 for the enthusiasm, and are pleased that the reviewer thinks the manuscript will be important to the field.

My main comment comes as a suggestion. While the paper is relatively easy to follow for someone already aware of microbiome / metagenomics studies, some additional information may be useful to users of the database not familiar (at all) with metagenomics data. In particular (i) a glossary of technical terms specific to (meta)genomics data and analysis (e.g., OTU, 16s, fasta/fastq), and

We thank Reviewer 1 for this suggestion. We have added the following glossary to our manuscript:
OTUOperational Taxonomic Unit, group of closely related organisms based on DNA sequence similarity.
16S16S ribosomal RNA gene, component of the prokaryotic ribosome, used to reconstruct phylogenies.
FASTAText-based format for representing nucleotide sequences with single-letter codes.
FASTQText-based format for representing nucleotide sequences and corresponding quality scores, with single-letter codes for nucleotides and quality.
TaxaGroups of one or more populations of organisms. Usually summarized at phylum, class, order, family, genus, or species levels.
MetadataDescriptive data pertaining to samples within a study
ShotgunShotgun metagenomics sequencing breaks up all available DNA into random small segments and uses chain termination to sequence reads. Reads can be aligned directly to a reference database, or overlapping reads can be assembled into contiguous sequences.
(ii) some additional details regarding the format of the data provided, especially the taxonomic information provided in the "taxatable" files. In the same spirit, it could be interesting to highlight an important specificity of microbiome data, namely that the input variables (taxa/OTUS) are ordered in a hierarchy. Dedicated machine learning methods exist (or could be developed) to take into account this type of data. Altogether, this could further motivate machine learning researchers interested in the analysis of structured data to use this repository.

We thank the reviewer for this suggestion and agree that the count table formats deserves more detail. We have added the following lines to the manuscript:

These counts are presented in tables that are organized as follows: OTUs or taxa as rows, and samples as columns. OTUs are represented as either NCBI genome identifiers or Greengenes identifiers. Taxa are represented as “kingdom; phylum; class; order; family; genus; species; strain”, with highest taxonomic specificity where possible.

Besides this general comment, I have a few questions that may deserve some clarifications in the main text :
* It is mentioned in page 3 that "full details regarding the data processing are provided for each dataset in the repository", but I am not sure to find them.

We apologize for the lack of detail here. We have updated this line to include details for where to find these preprocessing steps:

Full details regarding the data preprocessing are provided for each data set in the mlrepo-source branch of the GitHub repository, under preprocessing/make.mappings.r.

* I am not sure to understand what is meant by "samples with depths lower than 1000 sequences per samples were dropped". Do these 1000 sequences correspond to reads ? or to contigs ?
We apologize for the lack of clarity here, and would like to clarify that “1000 sequences” corresponds to sequencing reads. We dropped samples that contain less than a total of 1000 sequencing reads for 10 datasets, and less than a total of 100 sequencing reads for 5 datasets. The different thresholds were applied based on the

expected bacterial load of the sample types (e.g. colon biopsies are expected to have lower biomass than stool). We have updated the text as follows:

Samples with depths lower than 1000 sequencing reads per sample were dropped for n=10 datasets, while we applied a lower threshold of 100 sequencing reads per sample for n=5 datasets which had lower expected bacterial load.

* This may be obvious but I am not sure to understand how are defined OTUs from shotgun sequencing data. Are they based on the 16s gene only or is the entire genome used somehow?

We apologize for the lack of clarity. Shotgun sequencing data uses all of the available sequencing reads within a sample to identify the genomes that are present. We did not construct contigs, but instead mapped the sequencing reads directly to the reference database, which is composed of full genomes from the NCBI RefSeq prokaryote database. We have added the following text to the glossary:

Shotgun metagenomics sequencing breaks up all available DNA into random small segments and uses chain termination to sequence reads. Reads can be aligned directly to a reference database, or overlapping reads can be assembled into contigs.

* It is mentioned in page 3 that (i) "confounders were removed by dropping samples or stratification and (ii) "well-known confounders […] were accounted for when constructing prediction tasks". Could the authors be more specific about these (important) steps ?

We thank the reviewer for this excellent question. We have updated the text to better explain how we subset samples to address confounders. We have also provided the location of the R script that shows how we processed each original metadata file.

Well-known confounders were accounted for when constructing prediction tasks for other human-associated conditions; for example, predicting age using the Yatsunenko 2012 dataset is restricted to samples from the U.S. due to the known variation in gut microbiomes across different geographical locations. Details of how samples were subsetted for each prediction task can be found in the mlrepo-source branch of the GitHub repository, under preprocessing/make.mappings.r.

* In the same spirit, it is mentioned just after (top of page 4) that "confounders variables to control for" are reported in the tasks' metadata. This is indeed very valuable for the analysis and important to take into account. This is well explained in the Methods section, but I think it could be stressed in the main text (maybe simply by explicitly referring to the Methods section at this point).
We thank the reviewer for this suggestion and agree that more detail should be provided. We have updated the text as follows:

Hence, each prediction task is made available as an individual, compartmentalized metadata file that contains sample identifiers, responses to predict, and optionally, confounder variables that are inherent to the research study design such as paired healthy and diseased samples from the same subject (see Methods for more details).

* In the case study, it could be interesting to comment why RFs tend to do better than SVMs according to AUC but not accuracy.
We thank the reviewer for this suggestion. We have added an explanation to the text as follows:

We found that random forest accuracy improvements were moderate when compared with SVM-Linear (P=0.083) and SVM-Radial (P=0.03) [Fig 4B], which may be explained by the fact that, unlike AUC, accuracy ignores class prediction probability estimates.

* Since I assume that the number of OTUs will vary according to the nature of the samples (e.g., fewer in vaginal samples than stool samples), it could be interesting to mention in Table 1 the number of features involved in the various tasks. It could also be interesting to mention in this table whether the task involves classification or

Powered by Editorial Manager® and ProduXion Manager® from Aries Systems Corporation

regression.

We thank the reviewer for this excellent suggestion and have added an additional table (Table 2) that describes the prediction tasks, which includes the number of samples, number of features, and the response type, as seen below. A more detailed version of this table with additional columns of metadata is also available on GitHub under web/data/tasks.txt. Note that the number of features are provided on a per-dataset basis, and not on a per-task or per-attribute basis. Microbiome abundance tables inherently contain a superset of OTUs/Taxa found across all samples within a study, and we chose to leave these tables largely intact so that the end-user can have maximum flexibility in generating new prediction tasks from the original mapping file.

* Page 6 , lines 150-153. Could we simply say that this suggests that the OTU definitions made from GreenGenes and NCBI are consistent or is it more subtle? A comment on the respective merits (if any) of the two approaches (e.g., on the number of OTUs involved or in their level of taxonomic resolution) would be useful.

We thank the reviewer for this observation, and have added text to address the noted differences between these two references databases.

Note that OTU-picking against the Greengenes database resulted in more OTU features in every dataset [Table 2], hence, these findings may also highlight how the smaller, higher-quality NCBI RefSeq database can recover the same signal from the larger Greengenes database.

* I assume that the operation consisting in "collapsing OTUs at a complete-linkage correlation of 95%" mentioned in the Methods section (page 8, line 194) has something to do with "cutting" a dendrogram built from the OTU correlation matrix. Could the authors be a bit more specific ?

We apologize for the lack of clarity and have added additional text to this sentence to detail the steps taken to collapse correlated OTUs:

...collapsed at a complete-linkage correlation of 95% (which is done by calculating the Pearson’s correlation between each pair of OTUs using all complete pairs of observations, hierarchically clustering the results, and cutting the resulting dendrogram at a height of 0.05).

* For the sake of completeness, I think it would be worth detailing a bit more in the "case study benchmarking" section how were optimized the hyper-parameters of the machine learning algorithms considered (namely the regularization parameter for the SVMs, the bandwidth of the kernel for the radial-SVM and the number of trees for the RF). For instance : which grids of parameter values were considered, whether there was some kind of "nested" cross-validation to optimize the parameters before predicting the data for the held-out data, and on which criterion (e.g., accuracy or AUC) was/were chosen the optimal parameter(s).

We thank the reviewer for pointing this out. We did not perform hyper-parameter optimization, nor grid-searching. We have updated the manuscript text to better describe the model parameter settings as follows:

Control parameters were set using the function trainControl with parameter method = ‘none’ and default parameters. Default settings for all models are as follows: SVM radial basis sigma is set to .1, all SVMs C is set to 1, and randomForest number of trees is set to 500 and number of variables to split is sqrt(p), where p is the number of features.

Minor comments and typos :
* Page 1, line 16 : exist
Thank you for noting this. We have made this change:

Unfortunately, challenges still exist for machine learning algorithm developers who

Powered by Editorial Manager® and ProduXion Manager® from Aries Systems Corporation

often lack domain expertise required for interpretation and curation of the heterogeneous microbiome datasets.

* Page 2 , line 38 : the term "parse" is vague.
We have changed “parse” to “interpret”, as follows:

In addition, microbiome research data can be challenging to access and analyze for expert machine learning algorithm developers, who often do not have the domain expertise required to interpret the data and metadata in complex microbiome studies.

* Page 2, line 43 : "specifically for" may be replaced by "specific to" or "dedicated to"
We have changed “specifically for” to “dedicated to”, as follows:

Currently, we are unaware of any machine learning repository dedicated to microbiome classification tasks.

* Page 2, line 50 : "using" --> "involving" ; "curated" -->"derived"
We have made the suggested changes, and the updated text is now:

We present the Microbiome Learning Repo (ML Repo), a repository of 33 curated classification and regression tasks involving human microbiome data. Our 33 tasks are derived from 15 publicly available human microbiome datasets, which include 12 amplicon-based and 3 shotgun sequencing datasets [Table 1].

* Page 3, line 53 : "developer" --> "developers"
We have made the suggested change:

These datasets vary across sequencing technology platforms, 16s hypervariable regions, and study design, in order to help developers ensure robustness of algorithms across data types.

* Page 4, line 86 : "methods develope rs" --> "method developers" (for consistency with "machine learning algorithm developers" used several times before)
We have made the suggested change:

Generally, we expect that method developers will be most interested in sweeping through the full set of prediction tasks for benchmarking, and hence would prefer to download a single compressed file containing all tasks and data.

* Page 4, line 96 : "Sample Size and Response Type" --> "sample size and response type"
We have made the suggested change:

Task pages contain descriptive details such as sample size and response type that are specific to the selected prediction task, as well as links for downloading OTU tables, taxa tables, and sample metadata [Fig 2B].

* Page 5, line 115 : the term "nuances" is vague
We apologize for the lack of clarity here, and have updated the text as follows:

The subsetted samples found in each prediction task metadata file replaces the work of rigorously deciphering metadata and understanding the subtle differences of individual research studies.

Reviewer #2:
The paper by Vangay et al. presents "ML Repo", a public repository of microbiome datasets for conducting regression and classification analysis based on machine learning approaches. The repository is a web-based service and includes currently 33 curated classification and regression tasks from 15 published human microbiome datasets. The authors presents several use cases to demonstrate its wide application. The topic involved in the paper is suitable for publication in the GigaScience journal. The manuscript is well written and well structured. In general, the paper is a nice

contribution for the microbiome community.

We thank the reviewer for these comments and are pleased that the reviewer finds that our manuscript will be a contribution to the community.

However, I have some comments before a possible publication:
1. The novelty of the proposed repository needs to be better described. In particular, they authors missed to cite two recent and in some way similar repositories:
i. The "MicrobiomeHD" database, mainly for 16S studies: "Duvallet, Claire, Sean M. Gibbons, Thomas Gurry, Rafael A. Irizarry, and Eric J. Alm. "Meta-analysis of gut microbiome studies identifies disease-specific and shared responses." Nature communications 8, no. 1 (2017): 1784."
ii. The "curatedMetagenomicData" database, mainly for shotgun studies: "Pasolli, Edoardo, Lucas Schiffer, Paolo Manghi, Audrey Renson, Valerie Obenchain, Duy Tin Truong, Francesco Beghini et al. "Accessible, curated metagenomic data through ExperimentHub." Nature methods 14, no. 11 (2017): 1023."

I think these two contributions should be added in the section "Comparison to similar databases" and novelties of the proposed repository with respect to them properly discussed.

We thank the reviewer for excellent suggestion of adding these two papers, and apologize for not including them previously. We have added the following text in the section “Comparison to similar databases”:

Microbiome-based repositories that do provide manually curated metadata include curatedMetagenomicData and MicrobiomeHD. Although curatedMetagenomicData offers a collection of shotgun-metagenomics datasets with varying human sample types with gene, pathway, and taxonomic abundance tables, its data are accessible only via Bioconductor and are stored as ExpressionSet objects, which integrates metadata and abundance data. Although curatedMetagenomicData is an impressive repository with many features, it is most suitable for advanced bioinformaticians as its interface may hinder use by beginner data analysts and in teaching environments. MicrobiomeHD offers easily accessible taxonomic abundance tables with curated metadata, but is limited only to amplicon-based sequencing data, human stool samples, and case-control responses. And although both curatedMetagenomicData and MicrobiomeHD provide manually curated metadata, biological interpretation is still required as other sample metadata, for example antibiotic usage, may have biological relevance in predicting responses. This poses a potential problem for machine learning developers with limited biological and microbiome domain expertise. MLRepo resolves this issue by explicitly defining classification and regressions tasks for predicting responses that have been manually curated to either remove confounders or have been specifically annotated with biological confounders that must be controlled for. Metadata files in MLRepo are task-specific, and hence, are simplified to contain only: (1) sample identifiers indicating samples that should be used for the prediction task, (2) corresponding high-level phenotypes or responses, and optionally, (3) a confounder that should be accounted for due to its biological relevance. In addition, datasets in MLRepo include both amplicon-based and shotgun-metagenomics datasets covering a variety of human sample types, and are easily accessible via a web-interface.

2. The repository aims at providing metadata for both classification and regression tasks, as explicitly written also in the title. However, from my understanding use cases were reported on classification tasks only. Could you add some example on regression tasks?

We thank the reviewer for this suggestion. For space limitation reasons we have not added a whole section demonstrating a parameter sweep on the regression tasks. However, our work demonstrates that sweeping across parameters can inform future machine learning development efforts in regression, and we have emphasized the inclusion of regression tasks throughtout the mansucript.

3. Do you expect to add new datasets in the future? Can users contribute to them?

Please describe better this aspect in the paper and potentially on the website.

We do expect to add new datasets in the future, and also allow users to contribute to our repository. We apologize for not making the instructions more explicit. We have updated this section of our manuscript to point to the instructions for adding new datasets. We have provided the contents of https://github.com/knights-lab/MLRepo/blob/master/add-datasets-readme.md below (note that when viewed on GitHub, words referring to tools, databases, or GitHub tasks are hyperlinked to respective websites with instructions):
Steps for submitting a new dataset and/or task
1.If you have either the raw FASTQ or processed FASTA file, please deposit it into a public repository. We list large files via publicly accessible URLs and do not support uploading of any large files. If you need assistance, please contact us.
2.If starting with FASTQ, we recommend processing with SHI7 and OTU-picking with BURST, with NCBI RefSeq Prokaryote files and Green genes 97
3.Fork our repository.
4.Add new tasks and datasets directly into tasks and datasets. Make sure to fill out all sections.
5.We expect you to apply rigorous standards in filtering, subsetting, and selecting samples for your classification and regression tasks.
6.When ready, submit a pull request for our review.

We provide instructions on our GitHub repository (https://github.com/knights-lab/MLRepo/blob/master/add-datasets-readme.md) to guide users to create a fork from our repository, add the appropriate data and files, and update the master task and dataset lists.

4. Line 75: "Well-known confounders, such as geography, were accounted for when constructing prediction tasks for other human-associated conditions". I did not understand how this was really implemented in your analysis.

We thank the reviewer for pointing this out. We have updated the text below to better explain how we subset samples to address confounders. We have also provided the location of the R script that shows how we processed each original metadata file.

Well-known confounders were accounted for when constructing prediction tasks for other human-associated conditions; for example, predicting age using the Yatsunenko 2012 dataset is restricted to samples from the U.S. due to the known variation in gut microbiomes across different geographical locations. Details of how samples were subsetted for each prediction task can be found in the mlrepo-source branch of the GitHub repository, under preprocessing/make.mappings.r.

Reviewer #3:
In this contribution the authors present a repository of machine learning tasks, or 'challenges', concerning the prediction of a range of (human) host phenotypes from the composition of (one of) its microbiome(s). Other, non-phenotypical responses are concerned with host geographic location, body habitat, diet or antibiotic treatment. In general, this effort is highly appreciated, since the collection of suitable benchmark datasets and their standardisation is - at least - 50% of the work when developing machine learning (and other, baseline) methods.

The manuscript as a whole is in good shape and I specifically like the figures. My only real concern - and that's where the minor corrections come in - would be the general understandability of the manuscript to a non-microbiome audience, specifically to the envisaged (as one user type) CS-type user. The comparison to the UCI machine learning repository illustrates this concern best: what if a machine learning expert lacking _any_ biological background (i.e. not a microbiome-bioinformatics nor a bioinformatics but 'merely' an informatics person) was interested in your datasets and would read the paper as an introduction to using the data? Even with a bioinformatics background, while one will generally have heard about most of the basic concepts, it couldn't hurt to be reminded with some short additional explanations in the right places. I try to list those places in the following:

Powered by Editorial Manager® and ProduXion Manager® from Aries Systems Corporation

	p3,l56: 'We preprocessed raw sequences using...' - what do these tools do i.e. what does 'preprocessing' refer to exactly in this case?<br><br>We apologize for the lack of detail here and have updated the manuscript text to the following:<br><br>Raw sequences were trimmed and quality filtered using SHI7 [12] or QIIME [13].<br><br>p3,l59: 'We picked Operational Taxonomic Units (OTUs)...' - maybe briefly explain what this is, mainly the difference to the taxa counts<br><br>We thank the reviewer and agree that a definition is warranted. We have created a Glossary where we define OTU as follows:<br><br>OTUOperational Taxonomic Unit, group of closely related organisms based on DNA sequence similarity.<br><br>p3,l72: 'When available, published study exclusion criteria was [were!] applied accordingly...' - an example of such exclusion criteria would explain what you mean in an instant, just like you already do for the confounders<br><br>We thank the reviewer for the suggestion, and have updated the text as follows:<br><br>When available, published study exclusion criteria, such as reported use of antibiotics, were applied accordingly and confounders were removed by dropping samples or stratification.<br><br>p3,l77: '...to minimize the effect of high intra-individual similarities.' - it's not immediately obvious what you mean here, maybe rephrase? how does longitudinal data come in at all for your type of tasks? from reading it once i had the impression you'd have to select only one time point as the dataset for any given task anyway? i may well be missing a point here, so just make sure you spell it out as simple as you can.<br><br>We apologize for the lack of clarity and have replaced this text. We hope that the following text does a better job of explaining how we reduced the number of samples per subject.<br><br>Studies that were cross-sectional by design but contained several samples per subject were filtered to contain one sample per subject. In study designs with paired diseased-healthy or pre- and post-intervention samples, samples were reduced to two samples per subject with subject identifiers provided as confounder variables.<br><br>p8,l193: '...collapsed at a complete-linkage correlation of 95%.' - i totally didn't get this, it's definitely st you can save lots of (non-microbiome) people the time to think about by adding a short explanation<br><br>We apologize for the lack of clarification here and have updated the text as follows:<br><br>...collapsed at a complete-linkage correlation of 95% (which is done by calculating the Pearson’s correlation between each pair of OTUs using all complete pairs of observations, hierarchically clustering the results, and cutting the resulting dendrogram at a height of 0.05).<br><br>This may or may not be exhaustive but I hope you do get the general point. I think the necessary additions are rather small, st rephrasing may be sufficient, st just one more sentence. I suppose the 'luxury' solution would be having those amendmends done to the manuscript _and_ providing a glossary page on your website, just for the 5-10 relevant terms - but whether or not that additional effort makes sense for you and your target audience is st only you can decide. I would definitely hope that, with the suggest additional explanations, the manuscript and therefore resource may be helpful for a slightly wider audience than without them.<br><br>We greatly appreciate the suggestions that the reviewer has made, and have also added the glossary below to assist with some of these concerns.

OTUOperational Taxonomic Unit, group of closely related organisms based on DNA sequence similarity.
16S16S ribosomal RNA gene, component of the prokaryotic ribosome, used to reconstruct phylogenies.
FASTAText-based format for representing nucleotide sequences with single-letter codes.
FASTQText-based format for representing nucleotide sequences and corresponding quality scores, with single-letter codes for nucleotides and quality.
TaxaGroups of one or more populations of organisms. Usually summarized at phylum, class, order, family, genus, or species levels.
MetadataDescriptive data pertaining to samples within a study
ShotgunShotgun metagenomics sequencing breaks up all available DNA into random small segments and uses chain termination to sequence reads. Reads can be aligned directly to a reference database, or overlapping reads can be assembled into contiguous sequences.

Let me finish with some further, random points, in order of appearance:

general 1: Should already the abstract contain a link to your website? It's often done like that.

We have added the website URL to the abstract:
We present Microbiome Learning Repo (ML Repo, available at https://knights-lab.github.io/MLRepo/), a public, web-based repository of 33 curated classification and regression tasks from 15 published human microbiome datasets.

general 2: I was wondering whether or not the term 'task' could generally be replaced by 'challenge' to make it more clear?

We have decided not to change the term ‘task’ because this term is commonly used in the machine-learning community, especially when referring to prediction tasks, and we want to make the manuscript as accessible as possible to the machine learning community.

p3,l53: developer -> developers

This text has been updated as suggested.

p3,l56: I don't think the 'n=number' style of writing is necessary or in any way beneficial to the reader, so just put the number. This is true for all its occurrences throughout the manuscript.

We have removed ‘n=’ throughout the manuscript.

p3,l62: I may get it wrong but isn't the 'per sample' redundant here? Or do the two 'sample'-s in the first part of the sentence refer to two different things? And what does 'depths' mean? If it's just the number of sequences then 'Samples with less than...' would do the job and make reading easier. This issue recurs at least once below. So fix both in case.

We have updated the text as follows:

Samples with less than 1000 sequencing reads were dropped for 10 datasets, while we applied a lower threshold of 100 sequencing reads per sample for 5 datasets which had lower expected bacterial load.

p4,l96: You use uppercase very sparingly, can't see why to use it in 'Sample Size and Response Type' here.

This text has been updated as suggested.

p5,l105: Use it here, in 'Machine Learning Repository' instead, esp. given you do use it when mentioning this resource first.

Powered by Editorial Manager® and ProduXion Manager® from Aries Systems Corporation

	This text has been updated as suggested.<br><br>p5,l114: Can't you just scrap the 'prediction tasks.' here?<br><br>Yes, 'prediction tasks' have been removed from this text.<br><br>p5,l115: replaces -> replace<br><br>This text has been updated as suggested.<br><br>p5,l116: I don't quite understand how the 'Hence...' logically connects this sentence with the preceding one. Like, what have both to do with each other?<br><br>We agree with this observation. We have removed 'Hence' from this text.<br><br>p5,l125: Is it, logically speaking, not already 'for assigning' i.e. when you come up with high-level binary classes that later make your responses for prediction?<br><br>We have added extensive detail to this section describing what features discriminate ML Repo from, and make it more amenable to use by machine learning experts than, other efforts to collate microbiome data.<br><br>p6,l140: Here you comment on AUC being generally accepted etc, however, you already use it (without such a comment) earlier in the same para. Maybe revise slightly.<br><br>We thank the reviewer for pointing this out. We have moved this comment to the first mention of AUC, as follows:<br>Sweeping through available tasks with binary responses, we compare our models by examining receiver operating curves (ROCs) and areas under the curve (AUC), considered the standard method for machine learning model evaluation [23,24] [Fig 3].<br><br>p7,l160: This needs rephrasing! Right now you 'accept and merge' the actual researchers into your repo - they may not like this :)<br><br>We apologize for this incorrect phrasing. We have updated the text as follows:<br>Researchers can then submit a pull request for our review, and requests that are properly formatted will be accepted and merged into the repository<br><br>p8,l195: 'with smaller sample sizes' -> 'with fewer samples'?<br><br>This text has been updated as suggested.<br><br>One last important bit I just noticed: the mapping-orig.txt type links do not work for me as of 21/09/18 and must be fixed, e.g. https://knights-lab.github.io/MLRepo/docs/datasets/claesson/mapping-orig.txt.<br><br>We thank the reviewer for catching this important broken link. We have fixed our code and updated all of the original mapping file links. They should all be working now.
**Additional Information:**	
**Question**	**Response**
Are you submitting this manuscript to a special series or article collection?	No
**Experimental design and statistics**<br><br>Full details of the experimental design and statistical methods used should be given in the Methods section, as detailed in our Minimum Standards Reporting Checklist.	Yes

Powered by Editorial Manager® and ProduXion Manager® from Aries Systems Corporation

Information essential to interpreting the data presented should be made available in the figure legends.<br><br>Have you included all the information requested in your manuscript?	
**Resources**<br><br>A description of all resources used, including antibodies, cell lines, animals and software tools, with enough information to allow them to be uniquely identified, should be included in the Methods section. Authors are strongly encouraged to cite Research Resource Identifiers (RRIDs) for antibodies, model organisms and tools, where possible.<br><br>Have you included the information requested as detailed in our Minimum Standards Reporting Checklist?	Yes
**Availability of data and materials**<br><br>All datasets and code on which the conclusions of the paper rely must be either included in your submission or deposited in publicly available repositories (where available and ethically appropriate), referencing such data using a unique identifier in the references and in the “Availability of Data and Materials” section of your manuscript.<br><br>Have you have met the above requirement as detailed in our Minimum Standards Reporting Checklist?	Yes

Powered by Editorial Manager® and ProduXion Manager® from Aries Systems Corporation

Manuscript with DOI
Click here to access/download;Manuscript;MLRepo Manuscript - revised - final - 032319 w DOI.docx
Click here to view linked References

Microbiome Learning Repo (ML Repo): A public repository of microbiome regression and classification tasks

Pajau Vangay[1], Benjamin M. Hillmann[2], Dan Knights[12]*

[1]Bioinformatics and Computational Biology, University of Minnesota, Minneapolis, MN, 55455, vanga015@umn.edu, https://orcid.org/0000-0002-9231-0692

[2]Department of Computer Science and Engineering, University of Minnesota, Minneapolis, MN, 55455, hillm096@umn.edu, https://orcid.org/0000-0003-4276-1329

*Corresponding author: dknights@umn.edu, https://orcid.org/0000-0002-8205-251

Abstract

The use of machine learning in high-dimensional biological applications, such as the human microbiome, has grown exponentially in recent years, but algorithm developers often lack domain expertise required for interpretation and curation of the heterogeneous microbiome datasets. We present Microbiome Learning Repo (ML Repo, available at https://knights-lab.github.io/MLRepo/), a public, web-based repository of 33 curated classification and regression tasks from 15 published human microbiome datasets. We highlight the use of ML

Repo in several use cases to demonstrate its wide application, and expect it to be an important resource for algorithm developers.

Keywords

Microbiome, machine learning, repository, database

Findings

Background

Machine learning is widely used as a method for classification and prediction, with a growing number of applications in human health [1]. The use of machine learning in biological fields [2,3], and more specifically the microbiome research field [4–7], has grown exponentially due to the robustness of these algorithms to high dimensional data. However, challenges exist for large-scale meta-analysis as they often require manual curation of metadata and standardized processing of raw sequence data, resulting in variation in the results derived from chosen datasets across studies [8,9]. In addition, microbiome research data can be challenging to access and analyze for expert machine learning algorithm developers, who often do not have the domain expertise required to interpret the data and metadata in complex microbiome studies. There exist general resources with curated classification tasks from variety of domains. The University of California Irvine (UCI) Machine Learning Repository [10] revolutionized machine learning methods development by giving developers access to many curated datasets; its widespread usage and impact can be seen from its thousands of resulting citations. Currently, we are

unaware of any machine learning repository dedicated to microbiome classification tasks. We constructed a complementary database to address this deficiency, in order to promote the development of and usage of improved machine learning methods for the microbiome community.

Workflow

We present the Microbiome Learning Repo (ML Repo), a repository of 33 curated classification and regression tasks involving human microbiome data. Our 33 tasks are derived from 15 publicly available human microbiome datasets, which include 12 amplicon-based and 3 shotgun sequencing datasets [Table 1]. These datasets vary across sequencing technology platforms, 16s hypervariable regions, and study design, in order to help developers ensure robustness of algorithms across data types. We streamlined the microbiome data using a single post-processing workflow [Fig 1A]. We downloaded trimmed and quality filtered sequencing reads for 8 datasets from QIITA [11], and raw sequences for 7 datasets from public repositories. Raw sequences were trimmed and quality filtered using SHI7 [12] or QIIME [13]. We picked Operational Taxonomic Units (OTUs) from all quality filtered sequences using a closed-reference method with the BURST [14] aligner against both the NCBI RefSeq 16S ribosomal RNA project [15] and the Greengenes 97 database [16]. Samples with less than 1000 sequencing reads were dropped for 10 datasets, while we applied a lower threshold of 100 sequencing reads per sample for 5 datasets which had lower expected bacterial load. Full details regarding the data preprocessing are provided for each data set in the *mlrepo-source* branch of the GitHub repository, under preprocessing/make.mappings.r. As a result, for each dataset we generated RefSeq-based OTU and taxa abundance counts, and Greengenes-based OTU and taxa abundance counts. These counts are presented in tables that are organized as follows: OTUs or taxa as rows,

and samples as columns. OTUs are represented as either NCBI genome identifiers or Greengenes identifiers. Taxa are represented as "*kingdom; phylum; class; order; family; genus; species; strain"*, with highest taxonomic specificity where possible. We excluded additional post-processing filtering and normalization steps so that these parameters can be included in future benchmarking use cases as needed. We also limit our data to OTU and taxa tables as other metrics such as alpha and beta diversity can be subsequently generated as needed.

Sample metadata from individual studies were manually curated to generate viable prediction tasks. When available, published study exclusion criteria, such as reported use of antibiotics, were applied accordingly and confounders were removed by dropping samples or stratification. Well-known confounders were accounted for when constructing prediction tasks for other human-associated conditions; for example, predicting age using the *Yatsunenko 2012* dataset is restricted to samples from the U.S. due to the known variation in gut microbiomes across different geographical locations. Details of how samples were subset for each prediction task can be found in the *mlrepo-source* branch of the GitHub repository, under preprocessing/make.mappings.r. Studies that were cross-sectional by design but contained several samples per subject were filtered to contain one sample per subject. In study designs with paired diseased-healthy or pre- and post-intervention samples, samples were reduced to two samples per subject with subject identifiers provided as confounder variables. Hence, each prediction task is made available as an individual, compartmentalized metadata file that contains sample identifiers, responses to predict, and optionally, confounder variables that are inherent to the research study design such as paired healthy and diseased samples from the same subject (see

Methods for more details). As a result, we generated 33 distinct tasks for predicting human-associated responses.

Publicly available web-based interface

We expect two types of users: (1) machine-learning algorithm developers with limited knowledge of microbiome study designs and (2) microbiome researchers interested in obtaining additional datasets for meta-analysis. Generally, we expect that method developers will be most interested in sweeping through the full set of prediction tasks for benchmarking, and hence would prefer to download a single compressed file containing all tasks and data. On the other hand, we expect that microbiome researchers will be more selective in downloading specific datasets and tasks depending on their research domain. Hence, researchers may prefer to browse specific details about tasks and datasets prior to downloading.

Based on these expected use cases, we created a publicly available web-interface for ML Repo hosted by GitHub Pages [17]. Tasks are organized by relevant response categories [Fig 2A]. Task pages contain descriptive details such as sample size and response type that are specific to the selected prediction task, as well as links for downloading OTU tables, taxa tables, and sample metadata [Fig 2B]. Dataset pages contain important details about the entire dataset, including links to the original research study, as well as original metadata files and quality filtered sequences [Fig 2C]. We also provide a single compressed file containing the entire set of available tasks (OTU tables, taxa tables, and relevant metadata) for download from the main home page.

Benefits of curated microbiome-based prediction tasks

We expect ML Repo to be beneficial for both the machine-learning community as well as the microbiome research community. ML Repo will be a powerful complement to UCI's Machine Learning Repository, as it will allow for benchmarking curated classification tasks with high-dimensional data, and hence enable the subsequent development of novel algorithms for these complex datasets. Our streamlined approach in generating OTU and taxa tables offers a rich set of 15 datasets that microbiome researchers can use directly for further comparison with their own studies, for teaching and learning purposes, or for large meta-analyses. We expect that our provided OTU and taxa tables will also be beneficial for researchers with limited access to high-performance computing resources or bioinformatics skills necessary for processing raw sequencing data. In addition, we expect microbiome-specific methods development will also benefit from our repository. The subset of samples found in each prediction task metadata file replace the work of rigorously deciphering metadata and understanding the subtle differences of individual research studies. New methods, such as OTU-picking algorithms, can be evaluated not only on metrics such as speed and accuracy, but also based on overall impact to study findings.

Comparison to similar databases

Although a number of microbiome repositories exist, many are intended as data archival repositories [18,19] or function as resources for aggregating across studies [20]. Resources such as QIITA [11] offer an extensive collection of datasets, and mock-community-based Mockrobiota [21] is well-suited for benchmarking upstream methods, but neither offer support for the metadata interpretation necessary for predicting high-level phenotypes. Microbiome-based repositories that do provide manually curated metadata include curatedMetagenomicData [22] and MicrobiomeHD [23]. Although curatedMetagenomicData offers a collection of

shotgun-metagenomics datasets with varying human sample types with gene, pathway, and taxonomic abundance tables, its data are accessible only via Bioconductor [24] and are stored as ExpressionSet objects, which integrates metadata and abundance data. Although curatedMetagenomicData is an impressive repository with many features, it is most suitable for advanced bioinformaticians as its interface may hinder use by beginner data analysts and in teaching environments. MicrobiomeHD offers easily accessible taxonomic abundance tables with curated metadata, but is limited only to amplicon-based sequencing data, human stool samples, and case-control responses. And although both curatedMetagenomicData and MicrobiomeHD provide manually curated metadata, biological interpretation is still required as other sample metadata, for example antibiotic usage, may have biological relevance in predicting responses. This poses a potential problem for machine learning developers with limited biological and microbiome domain expertise. ML Repo resolves this issue by explicitly defining classification and regressions tasks for predicting responses that have been manually curated to either remove confounders or have been specifically annotated with biological confounders that must be controlled for. Metadata files in ML Repo are task-specific, and hence, are simplified to contain only: (1) sample identifiers indicating samples that should be used for the prediction task, (2) corresponding high-level phenotypes or responses, and optionally, (3) a confounder that should be accounted for due to its biological relevance. In addition, datasets in ML Repo include both amplicon-based and shotgun-metagenomics datasets covering a variety of human sample types, and are easily accessible via a web-interface.

Case studies

We compare the performance of three machine learning models: a random forest [25], and a support vector machine [26] (SVM) with either a radial or linear kernel. Sweeping through

available tasks with binary responses, we compare our models by examining receiver operating curves (ROCs) and areas under the curve (AUC), considered the standard method for machine learning model evaluation [27,28] [Fig 3]. Through comparison of ROCs, we can see that random forest outperforms or ties the other two models in 21 out of the 28 tasks. The choice of kernels for SVM appears to have limited impact on overall mean accuracy, yet a linear kernel can perfectly classify penicillin-treated and vancomycin-treated mouse cecal contents when the other models could not; further examination of the microbial features in these samples may be warranted to better understand the strengths of this kernel. We also performed pairwise comparisons of random forest against the other models across all tasks. When evaluated by AUC, random forest performs significantly better than both SVM with a linear kernel (P=0.0014) and with a radial kernel (P=0.00032) [Fig 4A]. We found that random forest accuracy improvements were moderate when compared with SVM-Linear (P=0.083) and SVM-Radial (P=0.03) [Fig 4B], which may be explained by the fact that, unlike AUC, accuracy ignores class prediction probability estimates. Our results support the broad usage [4,5,8,29] and acceptance of random forest as a robust classifier [6] with high-dimensional microbiome data.

To assess the impact of reference database choice on classification accuracies, we also used the classification tasks to compare random forest using OTUs picked with the Greengenes 97 database or the NCBI RefSeq Targeted Loci Project 16s project. We find that there is limited impact of database choice to overall classification accuracies [Fig 4C, Fig 5]. This may be due to (1) large effect sizes that are driven mainly by several well-characterized bacterial taxa present in both databases (e.g. stool versus tongue samples), or (2) small effect sizes such that classification is difficult regardless of the database (e.g. male versus female stool). Note that OTU-picking

with the Greengenes database resulted in more OTU features in every dataset [Table 2], hence, these findings further highlight how the smaller, higher-quality NCBI RefSeq database can recover the same signal from the larger Greengenes database.

Future work

We expect and hope that the broader microbiome research community will add new datasets and prediction tasks to ML Repo. We provide instructions [30] on our GitHub repository to guide users to create a fork from our repository, add the appropriate data and files, and update the master task and dataset lists. Researchers can then submit a pull request for our review, and requests that are properly formatted will be accepted and merged into the repository. We expect that data submissions will come from either the original researchers or those well-acquainted with the datasets, and hence will expect that sample selection and subsetting will have undergone rigorous review for prediction tasks.

Methods

Pre-processing of sequencing reads

When available, preprocessed FASTA files were downloaded from QIITA (or previously, the QIIME database). For all other datasets, raw FASTQ files were downloaded from sources listed in Supplemental Table 1. Adaptors and barcodes were removed and sequences were quality filtered (at Phred score ≥ Q20) using SHI7 [12] or QIIME [13]. OTUs were picked from processed FASTA files using BURST [31] with Greengenes [16] 97 or the NCBI RefSeq Targeted Loci Project 16s project [15] (accessed on 17-07-04). Samples with sequencing depth

lower than 1000 sequences per sample were dropped for all studies, except for five datasets [32–36], where the minimum threshold was 100 sequences per sample.

Selection of classification tasks

Classification tasks were selected based on reported study results, biologically relevant high-level phenotypes, and sufficient sample sizes. Original metadata files and research methods were rigorously and manually curated in order to subset samples with minimal confounders. For confounders that were inherent to the study, we include an additional variable to control for in the task metadata files. Presence of control variables can be found by examining "control_vars" in the Tasks table.

Website generation

Website templating was developed using Jinja2 [37] and custom Python scripts. Individual webpages were generated by iterating through items in the Tasks and Datasets tables, and dynamically populating templates in order to generate individual Markdown [38] pages. The resulting Markdown pages are hosted as GitHub Pages.

Case Study Benchmarking

Case study results were generated with custom R [39] scripts, which can be found in the */example* folder in the ML Repo Github repository. To compare machine learning models, we iterated through tasks with binary responses. OTU counts were converted to relative abundances, filtered at a minimum of 10% prevalence across samples, and collapsed at a complete-linkage correlation of 95% (which is done by calculating the Pearson's correlation between each pair of OTUs using all complete pairs of observations, hierarchically clustering the results, and cutting

the resulting dendrogram at a height of 0.05). We then constructed a 5-fold cross-validation for tasks containing more than 100 samples, or a leave-one-out cross-validation for tasks with fewer sample. For n-fold cross validation, samples were assigned to folds such that classes were equally balanced within each fold (e.g. if our task contained 40% healthy and 60% diseased samples, our folds would also be selected to represent this distribution). For tasks that contained control variables, we selected folds such that samples with the same control variable value were contained within the same fold. For example, for a task dataset containing matching stool and oral samples from subjects, the Subject Identifier would be listed as the control variable and we should assign samples to folds such that all samples from a specific subject were contained within a fold. This step is crucial to avoid biasing or overfitting the training model; test folds should contain not only new samples, but also samples that are independent from those in the training set. Models were constructed using the ‘caret’ package [40]. Control parameters were set using the function trainControl with parameter method = ‘none’ and default parameters. Default settings for all models are as follows: SVM radial basis sigma is set to .1, all SVMs C is set to 1, and randomForest number of trees is set to 500 and number of variables to split is sqrt(p), where p is the number of features. This entire process was bootstrapped 100 times, and the mean class probabilities were used to calculate the resulting AUCs and ROCs. To compare classification accuracies using different reference databases, we used a similar procedure but held the model constant and predicted using different base OTU tables. This framework enables comparison of a myriad of machine learning models available in the ‘caret’ package, and can be easily expanded to compare different OTU-picking algorithms, or normalization and filtering techniques.

Availability of supporting data

All test datasets are available in the Microbiome Learning Repo site [17], snapshots of our code and other supporting data are available in the *GigaScience* database, GigaDB [41].

Availability of supporting source code and requirements

Project name: Microbiome Learning Repo

Project home page: https://knights-lab.github.io/MLRepo/

Operating system: Platform independent

Programming language: Python, R

License: MIT License

Restrictions: None

RRID: SCR_017079

Glossary

OTU	Operational Taxonomic Unit, group of closely related organisms based on DNA sequence similarity.
16S	16S ribosomal RNA gene, component of the prokaryotic ribosome, used to reconstruct phylogenies.
FASTA	Text-based format for representing nucleotide sequences with single-letter codes.
FASTQ	Text-based format for representing nucleotide sequences and corresponding quality scores, with single-letter codes for nucleotides and quality.
Taxa	Groups of one or more populations of organisms. Usually summarized at phylum, class, order, family, genus, or species levels.

Metadata	Descriptive data pertaining to samples within a study
Shotgun	Shotgun metagenomics sequencing breaks up all available DNA into random small segments and uses chain termination to sequence reads. Reads can be aligned directly to a reference database, or overlapping reads can be assembled into contiguous sequences.

Declarations

Authors' contributions

Conceptualization: P.V. and D.K; Data curation: P.V.; Formal analyses: P.V.; Methodology: P.V., B.H., D.K.; Software: P.V.; Writing - original draft: P.V.; Writing - review and editing: B.H. and D.K.

Funding

This work is supported by funds from the National Institutes of Health Grant R01AI121383.

Competing Interests

D.K. serves as CEO and holds equity in CoreBiome, a company involved in the commercialization of microbiome analysis. The University of Minnesota also has financial interests in CoreBiome under the terms of a license agreement with CoreBiome. These interests have been reviewed and managed by the University of Minnesota in accordance with its Conflict-of-Interest policies.

References

1. Jordan MI, Mitchell TM. Machine learning: Trends, perspectives, and prospects. Science. 2015;349:255–60.

2. Shipp MA, Ross KN, Tamayo P, Weng AP, Kutok JL, Aguiar RCT, et al. Diffuse large B-cell lymphoma outcome prediction by gene-expression profiling and supervised machine learning. Nat Med. 2002;8:68–74.

3. Furey TS, Cristianini N, Duffy N, Bednarski DW, Schummer M, Haussler D. Support vector machine classification and validation of cancer tissue samples using microarray expression data. Bioinformatics. 2000;16:906–14.

4. Aagaard K, Riehle K, Ma J, Segata N, Mistretta T-A, Coarfa C, et al. A metagenomic approach to characterization of the vaginal microbiome signature in pregnancy. PLoS One. 2012;7:e36466.

5. Yatsunenko T, Rey FE, Manary MJ, Trehan I, Dominguez-Bello MG, Contreras M, et al. Human gut microbiome viewed across age and geography. Nature. 2012;486:222–7.

6. Knights D, Costello EK, Knight R. Supervised classification of human microbiota. FEMS Microbiol Rev. 2011;35:343–59.

7. Smith MI, Yatsunenko T, Manary MJ, Trehan I, Mkakosya R, Cheng J, et al. Gut microbiomes of Malawian twin pairs discordant for kwashiorkor. Science. 2013;339:548–54.

8. Pasolli E, Truong DT, Malik F, Waldron L, Segata N. Machine Learning Meta-analysis of Large Metagenomic Datasets: Tools and Biological Insights. PLoS Comput Biol. Public Library of Science; 2016;12:e1004977.

9. Sze MA, Schloss PD. Looking for a Signal in the Noise: Revisiting Obesity and the Microbiome. MBio [Internet]. 2016;7. Available from: http://dx.doi.org/10.1128/mBio.01018-16

10. Asuncion A, Newman D. UCI machine learning repository [Internet]. 2007. Available from: https://ergodicity.net/2013/07/

11. Qiita Development Team. QIITA [Internet]. Available from: http://qiita.microbio.me/

12. Al-Ghalith GA, Hillmann B, Ang K, Shields-Cutler R, Knights D. SHI7 Is a Self-Learning Pipeline for Multipurpose Short-Read DNA Quality Control. mSystems [Internet]. 2018;3. Available from: http://dx.doi.org/10.1128/mSystems.00202-17

13. Caporaso JG, Kuczynski J, Stombaugh J, Bittinger K, Bushman FD, Costello EK, et al. QIIME allows analysis of high-throughput community sequencing data. Nat Methods. 2010;7:335–6.

14. Al-Ghalith G, Knights D. BURST enables optimal exhaustive DNA alignment for big data [Internet]. Zenodo; 2017. Available from: http://dx.doi.org/10.5281/ZENODO.806850

15. O'Leary NA, Wright MW, Brister JR, Ciufo S, Haddad D, McVeigh R, et al. Reference sequence (RefSeq) database at NCBI: current status, taxonomic expansion, and functional annotation. Nucleic Acids Res. 2016;44:D733–45.

16. McDonald D, Price MN, Goodrich J, Nawrocki EP, DeSantis TZ, Probst A, et al. An improved Greengenes taxonomy with explicit ranks for ecological and evolutionary analyses of bacteria and archaea. ISME J. 2012;6:610–8.

17. Vangay P, Hillmann B, Knights D. MLRepo Github Page [Internet]. MLRepo. Available from: https://knights-lab.github.io/MLRepo

18. Hunter S, Corbett M, Denise H, Fraser M, Gonzalez-Beltran A, Hunter C, et al. EBI metagenomics—a new resource for the analysis and archiving of metagenomic data. Nucleic Acids Res. Oxford University Press; 2014;42:D600–6.

19. Leinonen R, Sugawara H, Shumway M, International Nucleotide Sequence Database Collaboration. The sequence read archive. Nucleic Acids Res. 2011;39:D19–21.

20. Forster SC, Browne HP, Kumar N, Hunt M, Denise H, Mitchell A, et al. HPMCD: the database of human microbial communities from metagenomic datasets and microbial reference genomes. Nucleic Acids Res. 2016;44:D604–9.

21. Bokulich NA, Rideout JR, Mercurio WG, Shiffer A, Wolfe B, Maurice CF, et al. mockrobiota: a Public Resource for Microbiome Bioinformatics Benchmarking. mSystems [Internet]. 2016;1. Available from: http://dx.doi.org/10.1128/mSystems.00062-16

22. Pasolli E, Schiffer L, Manghi P, Renson A, Obenchain V, Truong DT, et al. Accessible, curated metagenomic data through ExperimentHub. Nat Methods. 2017;14:1023–4.

23. Duvallet C, Gibbons SM, Gurry T, Irizarry RA, Alm EJ. Meta-analysis of gut microbiome studies identifies disease-specific and shared responses. Nat Commun. 2017;8:1784.

24. Gentleman RC, Carey VJ, Bates DM, Bolstad B, Dettling M, Dudoit S, et al. Bioconductor: open software development for computational biology and bioinformatics. Genome Biol. 2004;5:R80.

25. Breiman L. Random Forests. Mach Learn. 2001;45:5–32.

26. Cortes C, Vapnik V. Support-vector networks. Mach Learn. 1995;20:273–97.

27. Huang J, Ling CX. Using AUC and accuracy in evaluating learning algorithms. IEEE Trans Knowl Data Eng. 2005;17:299–310.

28. Ling CX, Huang J, Zhang H, Others. AUC: a statistically consistent and more discriminating measure than accuracy. IJCAI. 2003. p. 519–24.

29. Karlsson FH, Tremaroli V, Nookaew I, Bergström G, Behre CJ, Fagerberg B, et al. Gut metagenome in European women with normal, impaired and diabetic glucose control. Nature. 2013;498:99–103.

30. Vangay P, Hillmann B, Knights D. Instructions for adding new datasets [Internet]. MLRepo Github. Available from: https://github.com/knights-lab/MLRepo/blob/master/add-datasets-readme.md

31. Al-Ghalith G, Knights D. BURST enables optimal exhaustive DNA alignment for big data [Internet]. Zenodo; 2017. Available from: http://dx.doi.org/10.5281/ZENODO.806850

32. Gevers D, Kugathasan S, Denson LA, Vázquez-Baeza Y, Van Treuren W, Ren B, et al. The treatment-naive microbiome in new-onset Crohn's disease. Cell Host Microbe. 2014;15:382–92.

33. Human Microbiome Project Consortium. A framework for human microbiome research. Nature. 2012;486:215–21.

34. Kostic AD, Gevers D, Pedamallu CS, Michaud M, Duke F, Earl AM, et al. Genomic analysis identifies association of Fusobacterium with colorectal carcinoma. Genome Res. 2012;22:292–8.

35. David LA, Maurice CF, Carmody RN, Gootenberg DB, Button JE, Wolfe BE, et al. Diet rapidly and reproducibly alters the human gut microbiome. Nature. 2014;505:559–63.

36. Turnbaugh PJ, Hamady M, Yatsunenko T, Cantarel BL, Duncan A, Ley RE, et al. A core gut microbiome in obese and lean twins. Nature. 2009;457:480–4.

37. Ronacher A. Jinja2 [Internet]. 2017. Available from: http://jinja.pocoo.org/

38. Gruber J, Swartz A, Others. Markdown. 2004.

39. Team RC, Others. R: A language and environment for statistical computing. Citeseer; 2013; Available from: http://citeseerx.ist.psu.edu/viewdoc/download?doi=10.1.1.470.5851&rep=rep1&type=pdf

40. Kuhn M, Others. Caret package. J Stat Softw. 2008;28:1–26.

41. Vangay P; Hillmann BM; Knights D: Supporting data for "Microbiome Learning Repo (ML Repo): A public repository of microbiome regression and classification tasks" *GigaScience* Database. 2019. http://dx.doi.org/10.5524/100581.

Tables

Table 1. Microbiome datasets with available classification tasks in ML Repo.

Project Name	V Region	Target size	Num samples	Num subjects	Area	Description	Sequencing Technology	Study Design
Cho 2012	V3	177	95	47	Antibiotics	Mouse fecal and cecal samples, Control vs. 4 kinds of antibiotics	454	Cross-Sectional
Claesson 2012	V4	221	168	168	Age	Elderly and young adults	454	Cross-Sectional
David 2014	V4	282	235	11	Diet	Plant-based vs. Animal-based diet, Cross-over study	Illumina MiSeq	Longitudinal
Gevers 2014	V4	173	1321	668	IBD	Biopsies from IBD patients prior to treatment	Illumina MiSeq	Cross-Sectional
HMP 2012	V35	527	6407	242	Body Habitat, Gender	Up to 18 body sites across 242 healthy subjects at 1-2 time points	454	Cross-Sectional
Kostic 2012	V35	569	190	95	Colorectal Cancer	Adjacent Healthy vs. Tumor Colon Biopsy Tissues	454	Paired
Montassier 2016	V56	280	28	28	Bacteremia	Patients prior to chemotherapy who did or did not develop bacteremia	454	Cross-Sectional
Morgan 2012	V35	569	231	231	IBD	Healthy, Crohn's Disease, or Ulcerative Colitis patients	454	Cross-Sectional
Turnbaugh 2009	V2	230	281	154	Obesity	Monozygotic or dizygotic twin pairs concordant for BMI class, and their mothers	454	Cross-Sectional
Wu 2011	V12	244	95	10	Diet	Controlled HighFat or LowFat feeding on 10 subjects over 10 days	454	Longitudinal
Yatsunenko 2012	V4	282	531	531	Geography, Age, Gender	Humans of varying ages from the USA, Malawi, and Venezuela	Illumina MiSeq	Cross-Sectional
Ravel 2011	V12	240	396	396	Bacterial Vaginosis	Vaginal samples from four ethnic groups nugent scores for bacterial vaginosis	454	Cross-Sectional
Karlsson 2013	NA	NA	144	144	Diabetes	Patients with normal, impaired, or type 2 diabetes glucose tolerance categories	Illumina HiSeq	Cross-Sectional
Qin 2012	NA	NA	134	134	Diabetes	Healthy vs type 2 diabetes Chinese patients	Illumina HiSeq	Cross-Sectional
Qin 2014	NA	NA	130	130	Cirrhosis	Cirrhosis versus healthy	Illumina HiSeq	Cross-Sectional

ML Repo contains 33 classification and regression tasks from 15 publicly available human microbiome datasets shown here.

Table 2. Description of available prediction tasks

Dataset	attributes	description	area	Regression?	Sample Size	Number of Features: OTU, refseq	Number of Features: OTU, gg	Number of Features: Taxa, refseq	Number of Features: Taxa, gg	Control Vars
Cho 2012	Abx: Control, Chlortetracycline	Five groups of mice treated with four different antibiotics or no antibiotics	Antibiotics		47	293	1144	299	141	N
	Abx: Control, Chlortetracycline	Five groups of mice treated with four different antibiotics or no antibiotics	Antibiotics		45	293	1144	299	141	N
	Abx: Penicillin, Vancomycin	Five groups of mice treated with four different antibiotics or no antibiotics	Antibiotics		47	293	1144	299	141	N
	Abx: Penicillin, Vancomycin	Five groups of mice treated with four different antibiotics or no antibiotics	Antibiotics		45	293	1144	299	141	N
Claesson 2012	AGE: Elderly, Young	Elderly or young adults	Age		167	569	3763	662	279	N
David 2014	Diet: Plant, Animal	Individuals on the last day of an animal or plant diet intervention	Diet		18	1747	6293	1535	695	Y
Gevers 2014	DIAGNOSIS: no, CD	Healthy controls and Crohn's Disease patients	IBD		140	943	3547	992	446	N
	DIAGNOSIS: no, CD	Healthy controls and Crohn's Disease patients	IBD		160	943	3547	992	446	N
	PCDAI	PCDAI scores of CD patients at 6 months post sampling	IBD	X	68	943	3547	992	446	N
	PCDAI	PCDAI scores of CD patients at 6 months post sampling	IBD	X	51	943	3547	992	446	N
HMP 2012	HMPBODYSUPERSITE: Oral, Gastrointestinal_tract, HOST_SUBJECT_ID	Gastrointestinal tract and oral cavity of healthy adults	Body Habitat		2070	3121	9383	3090	1218	Y
	SEX: male, female	Healthy male and female adults	Gender		180	3121	9383	3090	1218	N
	HMPBODYSUBSITE: Stool, Tongue_dorsum; HOST_SUBJECT_ID	Stool and tongue of healthy adults	Body Habitat		404	3121	9383	3090	1218	Y
	HMPBODYSUBSITE: Subgingival_plaque, Supragingival_plaque; HOST_SUBJECT_ID	Subgingival and supragingival plague of healthy adults	Body Habitat		408	3121	9383	3090	1218	Y
Karlsson 2013	Classification: IGT, T2D	Impaired or type 2 diabetes glucose tolerance categories	Diabetes		101	12845	NA	3758	NA	N
	Classification: NGT, T2D	Normal or type 2 diabetes glucose tolerance categories	Diabetes		96	12845	NA	3758	NA	N
Kostic 2012	DIAGNOSIS: Healthy, Tumor; HOST_SUBJECT_ID	Colorectal carcinoma tumors and adjacent nonaffected tissues	Cancer		172	908	3228	980	409	Y
Montassier 2016	Treatment: bact, NObact	Patients prior to chemotherapy who did or did not develop bacteremia	Bacteremia		28	541	1852	640	228	N

Morgan 2012	ULCERATIVE_COLIT_OR_CROHNS_DIS: Crohn's disease, Healthy	Healthy, Crohn's Disease, or Ulcerative Colitis patients	IBD		128	829	3677	877	367	N
	ULCERATIVE_COLIT_OR_CROHNS_DIS: Ulcerative Colitis, Healthy	Healthy, Crohn's Disease, or Ulcerative Colitis patients	IBD		128	829	3677	877	367	N
Qin 2012	Diabetic: Y, N	Healthy or type 2 diabetes patients	Diabetes		124	11880	NA	2526	NA	N
Qin 2014	Cirrhotic: Cirrhosis, Healthy	Healthy or cirrhosis patients	Cirrhosis		130	8483	NA	2579	NA	N
Ravel 2011	Ethnic_Group: Black, Hispanic	Vaginal microbiomes of black and hispanic women	Vaginal		199	586	1093	660	305	N
	Nugent_score_category: low, high	Predict nugent score category (low, high) from vaginal microbiome	Vaginal		342	586	1093	660	305	N
	Nugent_score	Predict nugent score from vaginal microbiome	Vaginal	X	388	586	1093	660	305	N
	pH	Predict pH from vaginal microbiome	Vaginal	X	388	586	1093	660	305	N
	Ethnic_Group: White, Black	Vaginal microbiomes of white and black women	Vaginal		200	586	1093	660	305	N
Turnbaugh 2009	OBESITYCAT: Lean, Obese; ZYGOSITY: MZ, DZ, Mom	Lean or Obese individuals (monozygotic or dyzygotic twins or their mothers)	Obesity		142	557	4051	680	232	Y
Wu 2011	DIET: HighFat, LowFat	Individuals after completing a high fat or low fat diet intervention	Diet		10	292	1769	361	136	N
Yatsunenko 2012	AGE	Infants (up to Age 3) from the US	Age	X	49	4660	15783	4021	1544	N
	COUNTRY: GAZ:Venezuela, GAZ:Malawi	Individuals living in Malawi or Venezuela	Geography		54	4660	15783	4021	1544	N
	SEX: male, female	Males and females from the US	Gender		129	4660	15783	4021	1544	N
	COUNTRY: GAZ:United States of America, GAZ:Malawi	Individuals living in the US or Malawi	Geography		150	4660	15783	4021	1544	N

Figure Legends

Figure 1. Data processing workflow and website generation.

(A) Quality-filtered sequences were obtained from either the QIITA or from another public repository and trimmed and filtered using SHI7. Reference-based OTUs were picked using BURST with the NCBI RefSeq and Greengenes 97 databases.

(B) Individual GitHub Markdown pages were generated from dataset and task lists with a custom Python script and Jinja2 template, then uploaded to GitHub to be hosted.

Figure 2. Screenshots of ML Repo web interface.

(A) Available classification and regression tasks are listed by high level phenotype categories for browsing.

(B) Individual task webpages contain links to files for classifying a specific task, as well as relevant task-specific metadata.

(C) Individual dataset webpages contain relevant metadata pertaining to the entire dataset, as well as links to raw metadata files and sequencing data.

Figure 3. ROCs comparing random forest and SVM with different kernels.

Sweeping across all binary classification tasks available in ML Repo (28), we compare ROCs of random forest, SVM with a radial kernel, and SVM with a linear kernel. AUCs are listed within plots and are colored respective to each model.

Figure 4. Summary statistics of framework and database comparisons.

(A) AUCs random forest (rf) to SVM-Linear (left) and random forest to SVM-Radial (right). Paired t-tests reveal that random forest results in significantly higher AUC than both SVM-Linear (P=0.0014) and SVM-Radial (P=0.00032).

(B) Accuracies of random forest to SVM-Linear (left) and random forest to SVM-Radial (right). Paired t-tests reveal that random forest results in significantly better accuracy than SVM-Radial (P=0.03), but not SVM-Linear (P=0.083).

(C) AUCs (left) and accuracies (right) of random forest classifications of 24 tasks using OTUs picked with NCBI RefSeq database or Greengenes database as predictors. Student’s t-test reveals that reference database choice has limited impact on classification AUC or accuracy.

Lines are colored by the top model for each classification task.

Figure 5. ROCs comparing NCBI RefSeq and Greengenes 97 databases.

Sweeping across 16s-based binary classification tasks available in ML Repo (24), we compare ROCs of random forest with genus-level taxonomic summaries as predictors from OTU-picking strategies with the NCBI RefSeq prokaryote reference database and the Greengenes 97 reference database. AUCs are listed within plots and are colored respective to each database.

Figure 1 Click here to access/download;Figure;MLRepo Figure 1.pdf

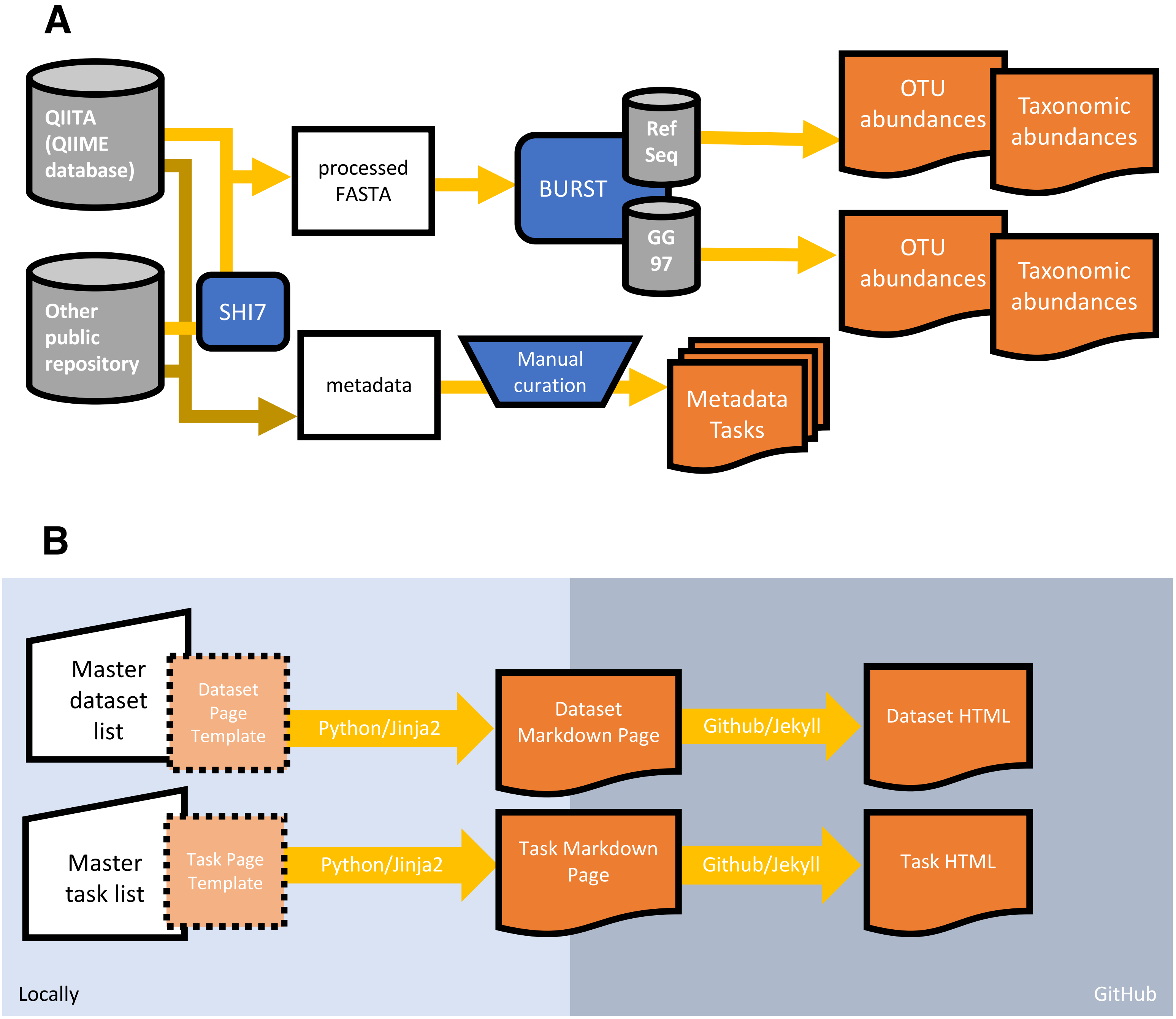

Figure 2

Click here to access/download;Figure;MLRepo Figure 2.pdf

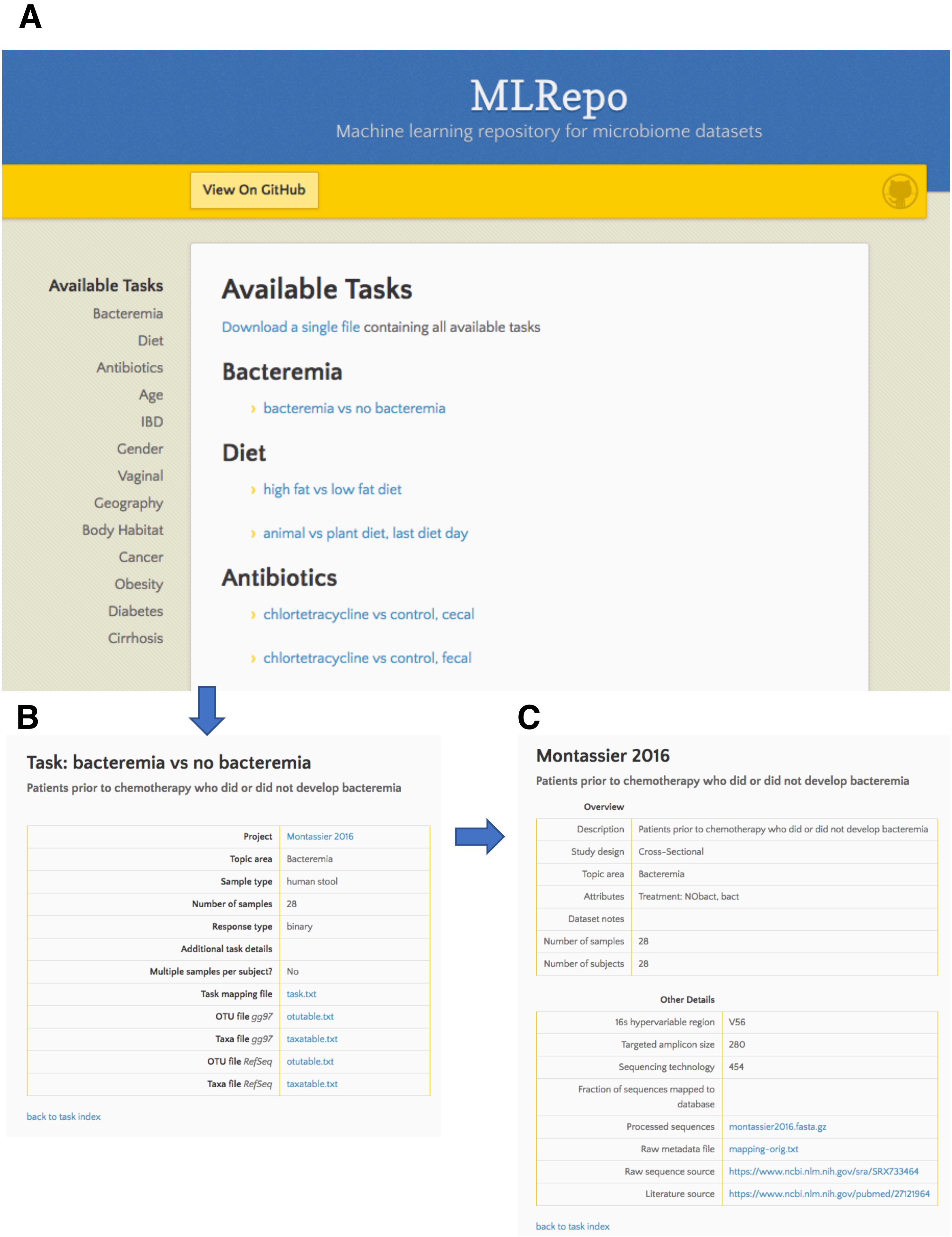

Figure 3

Click here to access/download;Figure;MLRepo Figure 3.pdf

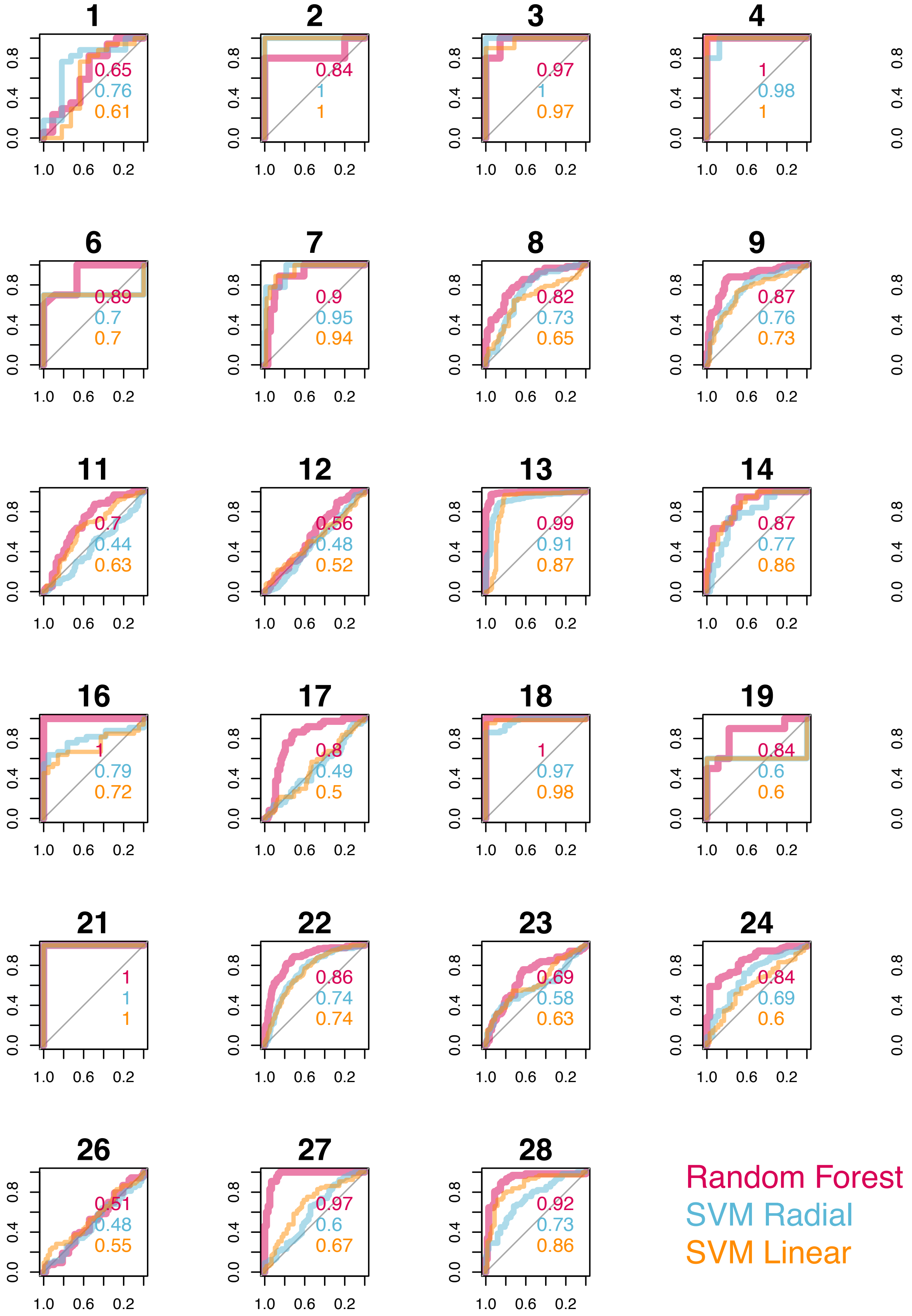

1 bacteremia vs no bacteremia
2 high fat vs low fat diet
3 chlortetracycline vs control, cecal
4 chlortetracycline vs control, fecal
5 penicillin vs vancomycin, cecal
6 penicillin vs vancomycin, fecal
7 elderly vs young
8 control vs cd, ileum
9 control vs cd, rectum
10 male vs female, stool
11 white vs black, vaginal
12 black vs hispanic, vaginal
13 low vs high nugent category
14 healthy vs cd, stool
15 healthy vs uc, stool
16 malawi vs venezuela, adults only
17 male vs female, usa
18 us vs malawi, adults only
19 animal vs plant diet, last diet day
20 gastrointestinal vs oral
21 stool vs tongue
22 subgingival vs supragingival plaque
23 healthy vs tumor biopsy, paired
24 lean vs obese, mz/dz/mom
25 normal vs diabetes glucose tolerance
26 impaired vs diabetes glucose tolerance
27 healthy vs type 2 diabetes
28 healthy vs cirrhosis

Figure 4

Click here to access/download;Figure;MLRepo Figure 4.pdf

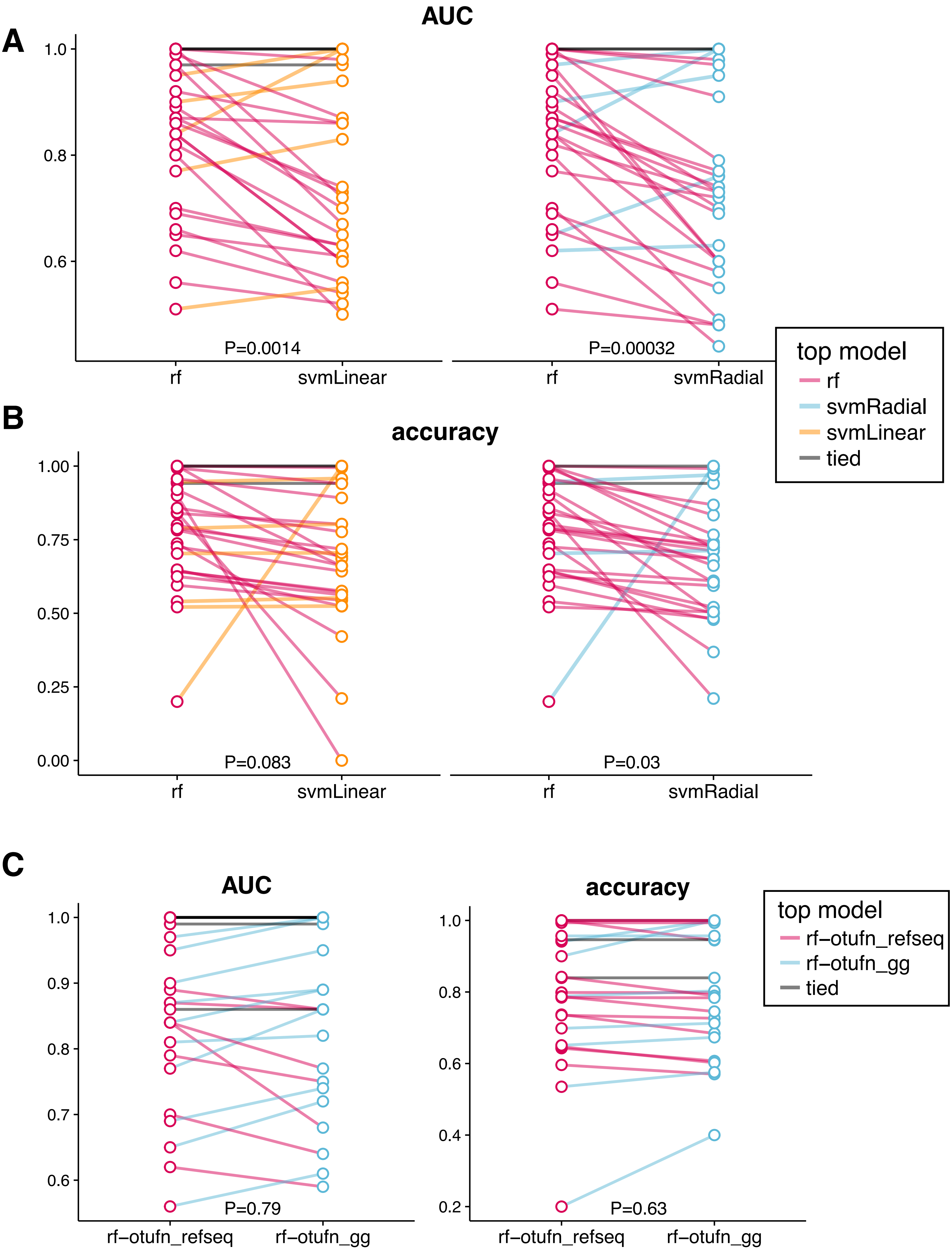

Figure 5 Click here to access/download;Figure;MLRepo Figure 5.pdf

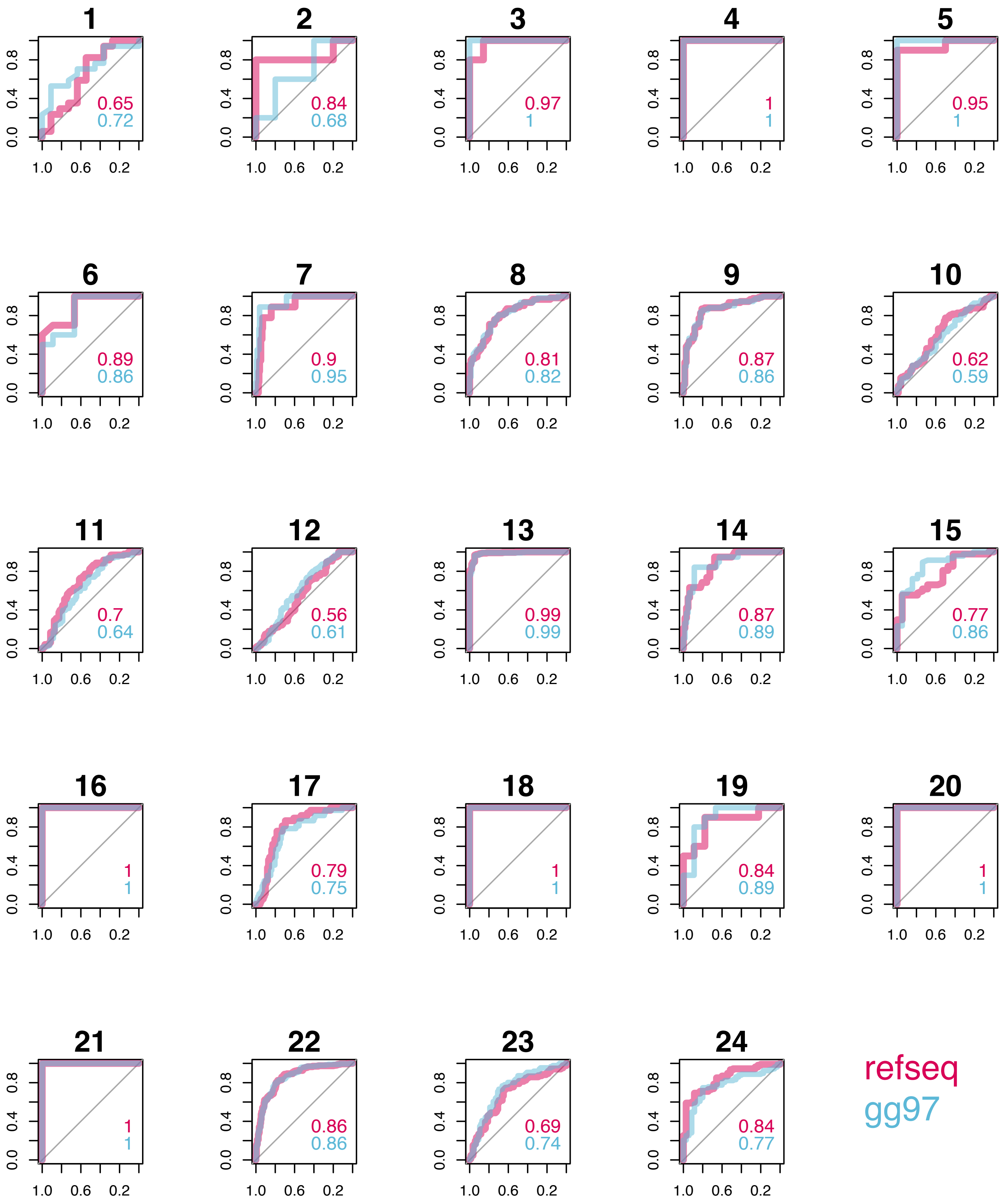

1 bacteremia vs no bacteremia
2 high fat vs low fat diet
3 chlortetracycline vs control, cecal
4 chlortetracycline vs control, fecal
5 penicillin vs vancomycin, cecal
6 penicillin vs vancomycin, fecal
7 elderly vs young
8 control vs cd, ileum
9 control vs cd, rectum
10 male vs female, stool
11 white vs black, vaginal
12 black vs hispanic, vaginal
13 low vs high nugent category
14 healthy vs cd, stool
15 healthy vs uc, stool
16 malawi vs venezuela, adults only
17 male vs female, usa
18 us vs malawi, adults only
19 animal vs plant diet, last diet day
20 gastrointestinal vs oral
21 stool vs tongue
22 subgingival vs supragingival plaque
23 healthy vs tumor biopsy, paired
24 lean vs obese, mz/dz/mom

Cover Letter Click here to access/download;Personal Cover;Cover_letter.pdf

UNIVERSITY OF MINNESOTA

Twin Cities Campus ***Biotechnology Institute*** *College of Biological Sciences* *420 Washington Ave SE* *Minneapolis, MN 55455*

February 24, 2019

Laurie Goodman, PhD
Editor in Chief, GigaScience

Dear Dr. Goodman and GigaScience Editorial Board,

We are pleased to submit our revised manuscript entitled "MLrepo: A public repository of microbiome regression and classification tasks" for your consideration for publication in *GigaScience*.

We are extremely grateful to the reviewers for their detailed commentary and suggestions, and for their very positive response to the manuscript overall. We have revised the manuscript extensively in accordance with reviewer suggestions, and we believe it substantially improved and is ready for publication.

We hope you will find this a valuable publication and resource to be shared with the GigaScience readership. Thank you in advance for your consideration. We look forward to your response. Please do not hesitate to contact us with any questions about the manuscript.

Yours Sincerely,

Dan Knights
Associate Professor
University of Minnesota

Click here to access/download;Personal Cover;MLRepo - Gigascience - Response to Reviewers.pdf

Response to Reviewers

Reviewer #1:
This paper describes MLRepo, a database of standardized microbiome datasets to develop and evaluate machine learning algorithms. The strength of this resource is to provide machine learning researchers a panel of diverse datasets (e.g., regression and classification tasks of various levels of complexity and with a various number of samples), already curated and formatted, to evaluate in an objective way novel algorithms dedicated to the analysis of microbiome samples. It can also be useful for teaching purposes (e.g., for practical lab sessions or to set up "data challenges") and will obviously be valuable to the community of microbiome researchers to set-up meta-analyses.

Overall I am very enthusiastic about this repository. As a machine-learning method developer interested in microbiome / metagenomics applications, I am indeed well aware that building curated databases and setting up baselines to evaluate novel algorithms is very time consuming, and that results reported in published papers are sometimes hard to reproduce. I am therefore convinced that this repository will simplify the whole process and facilitate evaluating algorithms in an objective way. I am therefore very favorable in having this work published in Giga Science.

We thank Reviewer 1 for the enthusiasm, and are pleased that the reviewer thinks the manuscript will be important to the field.

My main comment comes as a suggestion. While the paper is relatively easy to follow for someone already aware of microbiome / metagenomics studies, some additional information may be useful to users of the database not familiar (at all) with metagenomics data. In particular (i) a glossary of technical terms specific to (meta)genomics data and analysis (e.g., OTU, 16s, fasta/fastq), and

We thank Reviewer 1 for this suggestion. We have added the following glossary to our manuscript:

OTU	*Operational Taxonomic Unit, group of closely related organisms based on DNA sequence similarity.*
16S	*16S ribosomal RNA gene, component of the prokaryotic ribosome, used to reconstruct phylogenies.*
FASTA	*Text-based format for representing nucleotide sequences with single-letter codes.*
FASTQ	*Text-based format for representing nucleotide sequences and corresponding quality scores, with single-letter codes for*

nucleotides and quality.

Taxa	*Groups of one or more populations of organisms. Usually summarized at phylum, class, order, family, genus, or species levels.*
Metadata	*Descriptive data pertaining to samples within a study*
Shotgun	*Shotgun metagenomics sequencing breaks up all available DNA into random small segments and uses chain termination to sequence reads. Reads can be aligned directly to a reference database, or overlapping reads can be assembled into contiguous sequences.*

(ii) some additional details regarding the format of the data provided, especially the taxonomic information provided in the "taxatable" files. In the same spirit, it could be interesting to highlight an important specificity of microbiome data, namely that the input variables (taxa/OTUS) are ordered in a hierarchy. Dedicated machine learning methods exist (or could be developed) to take into account this type of data. Altogether, this could further motivate machine learning researchers interested in the analysis of structured data to use this repository.

We thank the reviewer for this suggestion and agree that the count table formats deserves more detail. We have added the following lines to the manuscript:

These counts are presented in tables that are organized as follows: OTUs or taxa as rows, and samples as columns. OTUs are represented as either NCBI genome identifiers or Greengenes identifiers. Taxa are represented as “kingdom; phylum; class; order; family; genus; species; strain”, with highest taxonomic specificity where possible.

Besides this general comment, I have a few questions that may deserve some clarifications in the main text :

* It is mentioned in page 3 that "full details regarding the data processing are provided for each dataset in the repository", but I am not sure to find them.

We apologize for the lack of detail here. We have updated this line to include details for where to find these preprocessing steps:

Full details regarding the data preprocessing are provided for each data set in the mlrepo-source branch of the GitHub repository, under preprocessing/make.mappings.r.

* I am not sure to understand what is meant by "samples with depths lower than 1000 sequences per samples were dropped". Do these 1000 sequences correspond to reads ? or to contigs ?

We apologize for the lack of clarity here, and would like to clarify that "1000 sequences" corresponds to sequencing reads. We dropped samples that contain less than a total of 1000 sequencing reads for 10 datasets, and less than a total of 100 sequencing reads for 5 datasets. The different thresholds were applied based on the expected bacterial load of the sample types (e.g. colon biopsies are expected to have lower biomass than stool). We have updated the text as follows:

Samples with depths lower than 1000 sequencing reads per sample were dropped for n=10 datasets, while we applied a lower threshold of 100 sequencing reads per sample for n=5 datasets which had lower expected bacterial load.

* This may be obvious but I am not sure to understand how are defined OTUs from shotgun sequencing data. Are they based on the 16s gene only or is the entire genome used somehow?

We apologize for the lack of clarity. Shotgun sequencing data uses all of the available sequencing reads within a sample to identify the genomes that are present. We did not construct contigs, but instead mapped the sequencing reads directly to the reference database, which is composed of full genomes from the NCBI RefSeq prokaryote database. We have added the following text to the glossary:

Shotgun metagenomics sequencing breaks up all available DNA into random small segments and uses chain termination to sequence reads. Reads can be aligned directly to a reference database, or overlapping reads can be assembled into contigs.

* It is mentioned in page 3 that (i) "confounders were removed by dropping samples or stratification and (ii) "well-known confounders […] were accounted for when constructing prediction tasks". Could the authors be more specific about these (important) steps ?

We thank the reviewer for this excellent question. We have updated the text to better explain how we subset samples to address confounders. We have also provided the location of the R script that shows how we processed each original metadata file.

Well-known confounders were accounted for when constructing prediction tasks for other human-associated conditions; for example, predicting age using the Yatsunenko 2012 dataset is restricted to samples from the U.S. due to the known variation in gut microbiomes across different geographical locations. Details of how samples were subsetted for each prediction task can be found in the mlrepo-source branch of the GitHub repository, under preprocessing/make.mappings.r.

* In the same spirit, it is mentioned just after (top of page 4) that "confounders variables to control for" are reported in the tasks' metadata. This is indeed very valuable for the analysis and important to take into account. This is well explained in the Methods section, but I think it could be stressed in the main text (maybe simply by explicitly referring to the Methods section at this point).

We thank the reviewer for this suggestion and agree that more detail should be provided. We have updated the text as follows:

Hence, each prediction task is made available as an individual, compartmentalized metadata file that contains sample identifiers, responses to predict, and optionally, confounder variables that are inherent to the research study design such as paired healthy and diseased samples from the same subject (see Methods for more details).

* In the case study, it could be interesting to comment why RFs tend to do better than SVMs according to AUC but not accuracy.

We thank the reviewer for this suggestion. We have added an explanation to the text as follows:

We found that random forest accuracy improvements were moderate when compared with SVM-Linear (P=0.083) and SVM-Radial (P=0.03) [Fig 4B], which may be explained by the fact that, unlike AUC, accuracy ignores class prediction probability estimates.

* Since I assume that the number of OTUs will vary according to the nature of the samples (e.g., fewer in vaginal samples than stool samples), it could be interesting to mention in Table 1 the number of features involved in the various tasks. It could also be interesting to mention in this table whether the task involves classification or regression.

We thank the reviewer for this excellent suggestion and have added an additional table (Table 2) that describes the prediction tasks, which includes the number of samples, number of features, and the response type, as seen below. A more detailed version of this table with additional columns of metadata is also available on GitHub under web/data/tasks.txt. Note that the number of features are provided on a per-dataset basis, and not on a per-task or per-attribute basis. Microbiome abundance tables inherently contain a superset of OTUs/Taxa found across all samples within a study, and we chose to leave these tables largely intact so that the end-user can have maximum flexibility in generating new prediction tasks from the original mapping file.

* Page 6 , lines 150-153. Could we simply say that this suggests that the OTU definitions made from GreenGenes and NCBI are consistent or is it more subtle? A comment on the respective merits (if any) of the two approaches (e.g., on the number of OTUs involved or in their level of taxonomic resolution) would be useful.

We thank the reviewer for this observation, and have added text to address the noted differences between these two references databases.

Note that OTU-picking against the Greengenes database resulted in more OTU features in every dataset [Table 2], hence, these findings may also highlight how the smaller,

higher-quality NCBI RefSeq database can recover the same signal from the larger Greengenes database.

* I assume that the operation consisting in "collapsing OTUs at a complete-linkage correlation of 95%" mentioned in the Methods section (page 8, line 194) has something to do with "cutting" a dendrogram built from the OTU correlation matrix. Could the authors be a bit more specific ?

We apologize for the lack of clarity and have added additional text to this sentence to detail the steps taken to collapse correlated OTUs:

...collapsed at a complete-linkage correlation of 95% (which is done by calculating the Pearson's correlation between each pair of OTUs using all complete pairs of observations, hierarchically clustering the results, and cutting the resulting dendrogram at a height of 0.05).

* For the sake of completeness, I think it would be worth detailing a bit more in the "case study benchmarking" section how were optimized the hyper-parameters of the machine learning algorithms considered (namely the regularization parameter for the SVMs, the bandwidth of the kernel for the radial-SVM and the number of trees for the RF). For instance : which grids of parameter values were considered, whether there was some kind of "nested" cross-validation to optimize the parameters before predicting the data for the held-out data, and on which criterion (e.g., accuracy or AUC) was/were chosen the optimal parameter(s).

We thank the reviewer for pointing this out. We did not perform hyper-parameter optimization, nor grid-searching. We have updated the manuscript text to better describe the model parameter settings as follows:

Control parameters were set using the function trainControl with parameter method = 'none' and default parameters. Default settings for all models are as follows: SVM radial basis sigma is set to .1, all SVMs C is set to 1, and randomForest number of trees is set to 500 and number of variables to split is sqrt(p), where p is the number of features.

Minor comments and typos :

* Page 1, line 16 : exist

Thank you for noting this. We have made this change:

Unfortunately, challenges still exist for machine learning algorithm developers who often lack domain expertise required for interpretation and curation of the heterogeneous microbiome datasets.

* Page 2 , line 38 : the term "parse" is vague.

We have changed "parse" to "interpret", as follows:

In addition, microbiome research data can be challenging to access and analyze for expert machine learning algorithm developers, who often do not have the domain expertise required to interpret the data and metadata in complex microbiome studies.

* Page 2, line 43 : "specifically for" may be replaced by "specific to" or "dedicated to"

We have changed "specifically for" to "dedicated to", as follows:

Currently, we are unaware of any machine learning repository dedicated to microbiome classification tasks.

* Page 2, line 50 : "using" --> "involving" ; "curated" -->"derived"

We have made the suggested changes, and the updated text is now:

We present the Microbiome Learning Repo (ML Repo), a repository of 33 curated classification and regression tasks involving human microbiome data. Our 33 tasks are derived from 15 publicly available human microbiome datasets, which include 12 amplicon-based and 3 shotgun sequencing datasets [Table 1].

* Page 3, line 53 : "developer" --> "developers"

We have made the suggested change:

These datasets vary across sequencing technology platforms, 16s hypervariable regions, and study design, in order to help developers ensure robustness of algorithms across data types.

* Page 4, line 86 : "methods develope rs" --> "method developers" (for consistency with "machine learning algorithm developers" used several times before)

We have made the suggested change:

Generally, we expect that method developers will be most interested in sweeping through the full set of prediction tasks for benchmarking, and hence would prefer to download a single compressed file containing all tasks and data.

* Page 4, line 96 : "Sample Size and Response Type" --> "sample size and response type"

We have made the suggested change:

Task pages contain descriptive details such as sample size and response type that are specific to the selected prediction task, as well as links for downloading OTU tables, taxa tables, and sample metadata [Fig 2B].

* Page 5, line 115 : the term "nuances" is vague

We apologize for the lack of clarity here, and have updated the text as follows:

The subsetted samples found in each prediction task metadata file replaces the work of rigorously deciphering metadata and understanding the subtle differences of individual research studies.

Reviewer #2:
The paper by Vangay et al. presents "ML Repo", a public repository of microbiome datasets for conducting regression and classification analysis based on machine learning approaches. The repository is a web-based service and includes currently 33 curated classification and regression tasks from 15 published human microbiome datasets. The authors presents several use cases to demonstrate its wide application. The topic involved in the paper is suitable for publication in the GigaScience journal. The manuscript is well written and well structured. In general, the paper is a nice contribution for the microbiome community.

We thank the reviewer for these comments and are pleased that the reviewer finds that our manuscript will be a contribution to the community.

However, I have some comments before a possible publication:
1. The novelty of the proposed repository needs to be better described. In particular, they authors missed to cite two recent and in some way similar repositories:
i. The "MicrobiomeHD" database, mainly for 16S studies: "Duvallet, Claire, Sean M. Gibbons, Thomas Gurry, Rafael A. Irizarry, and Eric J. Alm. "Meta-analysis of gut microbiome studies identifies disease-specific and shared responses." Nature communications 8, no. 1 (2017): 1784."
ii. The "curatedMetagenomicData" database, mainly for shotgun studies: "Pasolli, Edoardo, Lucas Schiffer, Paolo Manghi, Audrey Renson, Valerie Obenchain, Duy Tin Truong, Francesco Beghini et al. "Accessible, curated metagenomic data through ExperimentHub." Nature methods 14, no. 11 (2017): 1023."

I think these two contributions should be added in the section "Comparison to similar databases" and novelties of the proposed repository with respect to them properly discussed.

We thank the reviewer for excellent suggestion of adding these two papers, and apologize for not including them previously. We have added the following text in the section “Comparison to similar databases”:

Microbiome-based repositories that do provide manually curated metadata include curatedMetagenomicData and MicrobiomeHD. Although curatedMetagenomicData offers a collection of shotgun-metagenomics datasets with varying human sample types with gene, pathway, and taxonomic abundance tables, its data are accessible only via Bioconductor and are stored as ExpressionSet objects, which integrates metadata and abundance data. Although curatedMetagenomicData is an impressive repository with many features, it is most suitable for advanced bioinformaticians as its interface may hinder use by beginner data analysts and in teaching environments. MicrobiomeHD

offers easily accessible taxonomic abundance tables with curated metadata, but is limited only to amplicon-based sequencing data, human stool samples, and case-control responses. And although both curatedMetagenomicData and MicrobiomeHD provide manually curated metadata, biological interpretation is still required as other sample metadata, for example antibiotic usage, may have biological relevance in predicting responses. This poses a potential problem for machine learning developers with limited biological and microbiome domain expertise. MLRepo resolves this issue by explicitly defining classification and regressions tasks for predicting responses that have been manually curated to either remove confounders or have been specifically annotated with biological confounders that must be controlled for. Metadata files in MLRepo are task-specific, and hence, are simplified to contain only: (1) sample identifiers indicating samples that should be used for the prediction task, (2) corresponding high-level phenotypes or responses, and optionally, (3) a confounder that should be accounted for due to its biological relevance. In addition, datasets in MLRepo include both amplicon-based and shotgun-metagenomics datasets covering a variety of human sample types, and are easily accessible via a web-interface.

2. The repository aims at providing metadata for both classification and regression tasks, as explicitly written also in the title. However, from my understanding use cases were reported on classification tasks only. Could you add some example on regression tasks?

We thank the reviewer for this suggestion. For space limitation reasons we have not added a whole section demonstrating a parameter sweep on the regression tasks. However, our work demonstrates that sweeping across parameters can inform future machine learning development efforts in regression, and we have emphasized the inclusion of regression tasks throughtout the mansucript.

3. Do you expect to add new datasets in the future? Can users contribute to them? Please describe better this aspect in the paper and potentially on the website.

We do expect to add new datasets in the future, and also allow users to contribute to our repository. We apologize for not making the instructions more explicit. We have updated this section of our manuscript to point to the instructions for adding new datasets. We have provided the contents of https://github.com/knights-lab/MLRepo/blob/master/add-datasets-readme.md below (note that when viewed on GitHub, words referring to tools, databases, or GitHub tasks are hyperlinked to respective websites with instructions):

Steps for submitting a new dataset and/or task

1. *If you have either the raw FASTQ or processed FASTA file, please deposit it into a public repository. We list large files via publicly accessible URLs and do not support uploading of any large files. If you need assistance, please contact us.*

2. *If starting with FASTQ, we recommend processing with SHI7 and OTU-picking with BURST, with NCBI RefSeq Prokaryote files and Green genes 97*
3. *Fork our repository.*
4. *Add new tasks and datasets directly into tasks and datasets. Make sure to fill out all sections.*
5. *We expect you to apply rigorous standards in filtering, subsetting, and selecting samples for your classification and regression tasks.*
6. *When ready, submit a pull request for our review.*

We provide instructions on our GitHub repository (https://github.com/knights-lab/MLRepo/blob/master/add-datasets-readme.md) to guide users to create a fork from our repository, add the appropriate data and files, and update the master task and dataset lists.

4. Line 75: "Well-known confounders, such as geography, were accounted for when constructing prediction tasks for other human-associated conditions". I did not understand how this was really implemented in your analysis.

We thank the reviewer for pointing this out. We have updated the text below to better explain how we subset samples to address confounders. We have also provided the location of the R script that shows how we processed each original metadata file.

Well-known confounders were accounted for when constructing prediction tasks for other human-associated conditions; for example, predicting age using the Yatsunenko 2012 dataset is restricted to samples from the U.S. due to the known variation in gut microbiomes across different geographical locations. Details of how samples were subsetted for each prediction task can be found in the mlrepo-source branch of the GitHub repository, under preprocessing/make.mappings.r.

Reviewer #3:
In this contribution the authors present a repository of machine learning tasks, or 'challenges', concerning the prediction of a range of (human) host phenotypes from the composition of (one of) its microbiome(s). Other, non-phenotypical responses are concerned with host geographic location, body habitat, diet or antibiotic treatment. In general, this effort is highly appreciated, since the collection of suitable benchmark datasets and their standardisation is - at least - 50% of the work when developing machine learning (and other, baseline) methods.

The manuscript as a whole is in good shape and I specifically like the figures. My only real concern - and that's where the minor corrections come in - would be the general understandability of the manuscript to a non-microbiome audience, specifically to the envisaged (as one user type) CS-type user. The comparison to the UCI machine learning repository illustrates this concern best: what if a machine learning expert lacking _any_ biological

background (i.e. not a microbiome-bioinformatics nor a bioinformatics but 'merely' an informatics person) was interested in your datasets and would read the paper as an introduction to using the data? Even with a bioinformatics background, while one will generally have heard about most of the basic concepts, it couldn't hurt to be reminded with some short additional explanations in the right places. I try to list those places in the following:

p3,l56: 'We preprocessed raw sequences using...' - what do these tools do i.e. what does 'preprocessing' refer to exactly in this case?

> *We apologize for the lack of detail here and have updated the manuscript text to the following:*
>
> *Raw sequences were trimmed and quality filtered using SHI7 [12] or QIIME [13].*

p3,l59: 'We picked Operational Taxonomic Units (OTUs)...' - maybe briefly explain what this is, mainly the difference to the taxa counts

> *We thank the reviewer and agree that a definition is warranted. We have created a Glossary where we define OTU as follows:*
>
> *OTU* — *Operational Taxonomic Unit, group of closely related organisms based on DNA sequence similarity.*

p3,l72: 'When available, published study exclusion criteria was [were!] applied accordingly...' - an example of such exclusion criteria would explain what you mean in an instant, just like you already do for the confounders

> *We thank the reviewer for the suggestion, and have updated the text as follows:*
>
> *When available, published study exclusion criteria, such as reported use of antibiotics, were applied accordingly and confounders were removed by dropping samples or stratification.*

p3,l77: '...to minimize the effect of high intra-individual similarities.' - it's not immediately obvious what you mean here, maybe rephrase? how does longitudinal data come in at all for your type of tasks? from reading it once i had the impression you'd have to select only one time point as the dataset for any given task anyway? i may well be missing a point here, so just make sure you spell it out as simple as you can.

> *We apologize for the lack of clarity and have replaced this text. We hope that the following text does a better job of explaining how we reduced the number of samples per subject.*

Studies that were cross-sectional by design but contained several samples per subject were filtered to contain one sample per subject. In study designs with paired diseased-healthy or pre- and post-intervention samples, samples were reduced to two samples per subject with subject identifiers provided as confounder variables.

p8,l193: '...collapsed at a complete-linkage correlation of 95%.' - i totally didn't get this, it's definitely st you can save lots of (non-microbiome) people the time to think about by adding a short explanation

We apologize for the lack of clarification here and have updated the text as follows:

...collapsed at a complete-linkage correlation of 95% (which is done by calculating the Pearson's correlation between each pair of OTUs using all complete pairs of observations, hierarchically clustering the results, and cutting the resulting dendrogram at a height of 0.05).

This may or may not be exhaustive but I hope you do get the general point. I think the necessary additions are rather small, st rephrasing may be sufficient, st just one more sentence. I suppose the 'luxury' solution would be having those amendmends done to the manuscript _and_ providing a glossary page on your website, just for the 5-10 relevant terms - but whether or not that additional effort makes sense for you and your target audience is st only you can decide. I would definitely hope that, with the suggest additional explanations, the manuscript and therefore resource may be helpful for a slightly wider audience than without them.

We greatly appreciate the suggestions that the reviewer has made, and have also added the glossary below to assist with some of these concerns.

OTU	*Operational Taxonomic Unit, group of closely related organisms based on DNA sequence similarity.*
16S	*16S ribosomal RNA gene, component of the prokaryotic ribosome, used to reconstruct phylogenies.*
FASTA	*Text-based format for representing nucleotide sequences with single-letter codes.*
FASTQ	*Text-based format for representing nucleotide sequences and corresponding quality scores, with single-letter codes for nucleotides and quality.*
Taxa	*Groups of one or more populations of organisms. Usually summarized at phylum, class, order, family, genus, or species levels.*

Metadata	*Descriptive data pertaining to samples within a study*
Shotgun	*Shotgun metagenomics sequencing breaks up all available DNA into random small segments and uses chain termination to sequence reads. Reads can be aligned directly to a reference database, or overlapping reads can be assembled into contiguous sequences.*

Let me finish with some further, random points, in order of appearance:

general 1: Should already the abstract contain a link to your website? It's often done like that.

We have added the website URL to the abstract:
We present Microbiome Learning Repo (ML Repo, available at https://knights-lab.github.io/MLRepo/), a public, web-based repository of 33 curated classification and regression tasks from 15 published human microbiome datasets.

general 2: I was wondering whether or not the term 'task' could generally be replaced by 'challenge' to make it more clear?

We have decided not to change the term 'task' because this term is commonly used in the machine-learning community, especially when referring to prediction tasks, and we want to make the manuscript as accessible as possible to the machine learning community.

p3,l53: developer -> developers

This text has been updated as suggested.

p3,l56: I don't think the 'n=number' style of writing is necessary or in any way beneficial to the reader, so just put the number. This is true for all its occurrences throughout the manuscript.

We have removed 'n=' throughout the manuscript.

p3,l62: I may get it wrong but isn't the 'per sample' redundant here? Or do the two 'sample'-s in the first part of the sentence refer to two different things? And what does 'depths' mean? If it's just the number of sequences then 'Samples with less than...' would do the job and make reading easier. This issue recurs at least once below. So fix both in case.

We have updated the text as follows:

Samples with less than 1000 sequencing reads were dropped for 10 datasets, while we applied a lower threshold of 100 sequencing reads per sample for 5 datasets which had lower expected bacterial load.

p4,l96: You use uppercase very sparingly, can't see why to use it in 'Sample Size and Response Type' here.

This text has been updated as suggested.

p5,l105: Use it here, in 'Machine Learning Repository' instead, esp. given you do use it when mentioning this resource first.

This text has been updated as suggested.

p5,l114: Can't you just scrap the 'prediction tasks.' here?

Yes, ‘prediction tasks’ have been removed from this text.

p5,l115: replaces -> replace

This text has been updated as suggested.

p5,l116: I don't quite understand how the 'Hence...' logically connects this sentence with the preceding one. Like, what have both to do with each other?

We agree with this observation. We have removed ‘Hence’ from this text.

p5,l125: Is it, logically speaking, not already 'for assigning' i.e. when you come up with high-level binary classes that later make your responses for prediction?

We have added extensive detail to this section describing what features discriminate ML Repo from, and make it more amenable to use by machine learning experts than, other efforts to collate microbiome data.

p6,l140: Here you comment on AUC being generally accepted etc, however, you already use it (without such a comment) earlier in the same para. Maybe revise slightly.

We thank the reviewer for pointing this out. We have moved this comment to the first mention of AUC, as follows:
Sweeping through available tasks with binary responses, we compare our models by examining receiver operating curves (ROCs) and areas under the curve (AUC), considered the standard method for machine learning model evaluation [23,24] [Fig 3].

p7,l160: This needs rephrasing! Right now you 'accept and merge' the actual researchers into your repo - they may not like this :)

> *We apologize for this incorrect phrasing. We have updated the text as follows: Researchers can then submit a pull request for our review, and requests that are properly formatted will be accepted and merged into the repository*

p8,l195: 'with smaller sample sizes' -> 'with fewer samples'?

> *This text has been updated as suggested.*

One last important bit I just noticed: the mapping-orig.txt type links do not work for me as of 21/09/18 and must be fixed, e.g. https://knights-lab.github.io/MLRepo/docs/datasets/claesson/mapping-orig.txt.

> *We thank the reviewer for catching this important broken link. We have fixed our code and updated all of the original mapping file links. They should all be working now.*
