# Supplementary material for: Microbiome Learning Repo (ML Repo): A public repository of microbiome regression and classification tasks
Source: Gigascience. 2019 Apr 26;8(5):giz042. doi: 10.1093/gigascience/giz042 (PMC6493971; doi:10.1093/gigascience/giz042)
Supplement: Response_to_Reviewer_Comments_Original_Submission.pdf [file giz042_response_to_reviewer_comments_original_submission.pdf]

Author's Response To Reviewer Comments

Close

Response to Reviewers

Reviewer #1:
This paper describes MLRepo, a database of standardized microbiome datasets to develop and evaluate machine learning algorithms. The strength of this resource is to provide machine learning researchers a panel of diverse datasets (e.g., regression and classification tasks of various levels of complexity and with a various number of samples), already curated and formatted, to evaluate in an objective way novel algorithms dedicated to the analysis of microbiome samples. It can also be useful for teaching purposes (e.g., for practical lab sessions or to set up "data challenges") and will obviously be valuable to the community of microbiome researchers to set-up meta-analyses.

Overall I am very enthusiastic about this repository. As a machine-learning method developer interested in microbiome / metagenomics applications, I am indeed well aware that building curated databases and setting up baselines to evaluate novel algorithms is very time consuming, and that results reported in published papers are sometimes hard to reproduce. I am therefore convinced that this repository will simplify the whole process and facilitate evaluating algorithms in an objective way. I am therefore very favorable in having this work published in Giga Science.

We thank Reviewer 1 for the enthusiasm, and are pleased that the reviewer thinks the manuscript will be important to the field.

My main comment comes as a suggestion. While the paper is relatively easy to follow for someone already aware of microbiome / metagenomics studies, some additional information may be useful to users of the database not familiar (at all) with metagenomics data. In particular (i) a glossary of technical terms specific to (meta)genomics data and analysis (e.g., OTU, 16s, fasta/fastq), and

We thank Reviewer 1 for this suggestion. We have added the following glossary to our manuscript:
OTU Operational Taxonomic Unit, group of closely related organisms based on DNA sequence similarity.
16S 16S ribosomal RNA gene, component of the prokaryotic ribosome, used to reconstruct phylogenies.
FASTA Text-based format for representing nucleotide sequences with single-letter codes.
FASTQ Text-based format for representing nucleotide sequences and corresponding quality scores, with single-letter codes for nucleotides and quality.
Taxa Groups of one or more populations of organisms. Usually summarized at phylum, class, order, family, genus, or species levels.
Metadata Descriptive data pertaining to samples within a study
Shotgun Shotgun metagenomics sequencing breaks up all available DNA into random small segments and uses chain termination to sequence reads. Reads can be aligned directly to a reference database, or overlapping reads can be assembled into contiguous sequences.
(ii) some additional details regarding the format of the data provided, especially the taxonomic information provided in the "taxatable" files. In the same spirit, it could be interesting to highlight an important specificity of microbiome data, namely that the input variables (taxa/OTUS) are ordered in a hierarchy. Dedicated machine learning methods exist (or could be developed) to take into account this type of data. Altogether, this could further motivate machine learning researchers interested in the analysis of structured data to use this repository.

We thank the reviewer for this suggestion and agree that the count table formats deserves more detail. We have added the following lines to the manuscript:

These counts are presented in tables that are organized as follows: OTUs or taxa as rows, and samples as columns. OTUs are represented as either NCBI genome identifiers or Greengenes identifiers. Taxa are represented as “kingdom; phylum; class; order; family; genus; species; strain”, with highest taxonomic specificity where possible.

Besides this general comment, I have a few questions that may deserve some clarifications in the main text :
* It is mentioned in page 3 that "full details regarding the data processing are provided for each dataset in the repository", but I am not sure to find them.

We apologize for the lack of detail here. We have updated this line to include details for where to find these preprocessing steps:

Full details regarding the data preprocessing are provided for each data set in the mlrepo-source branch of the GitHub repository, under preprocessing/make.mappings.r.

* I am not sure to understand what is meant by "samples with depths lower than 1000 sequences per samples were dropped". Do these 1000 sequences correspond to reads ? or to contigs ?
We apologize for the lack of clarity here, and would like to clarify that "1000 sequences" corresponds to sequencing reads. We dropped samples that contain less than a total of 1000 sequencing reads for 10 datasets, and less than a total of 100 sequencing reads for 5 datasets. The different thresholds were applied based on the expected bacterial load of the sample types (e.g. colon biopsies are expected to have lower biomass than stool). We have updated the text as follows:

Samples with depths lower than 1000 sequencing reads per sample were dropped for n=10 datasets, while we applied a lower threshold of 100 sequencing reads per sample for n=5 datasets which had lower expected bacterial load.

* This may be obvious but I am not sure to understand how are defined OTUs from shotgun sequencing data. Are they based on the 16s gene only or is the entire genome used somehow?

We apologize for the lack of clarity. Shotgun sequencing data uses all of the available sequencing reads within a sample to identify the genomes that are present. We did not construct contigs, but instead mapped the sequencing reads directly to the reference database, which is composed of full genomes from the NCBI RefSeq prokaryote database. We have added the following text to the glossary:

Shotgun metagenomics sequencing breaks up all available DNA into random small segments and uses chain termination to sequence reads. Reads can be aligned directly to a reference database, or overlapping reads can be assembled into contigs.

* It is mentioned in page 3 that (i) "confounders were removed by dropping samples or stratification and (ii) "well-known confounders […] were accounted for when constructing prediction tasks". Could the authors be more specific about these (important) steps ?

We thank the reviewer for this excellent question. We have updated the text to better explain how we subset samples to address confounders. We have also provided the location of the R script that shows how we processed each original metadata file.

Well-known confounders were accounted for when constructing prediction tasks for other human-associated conditions; for example, predicting age using the Yatsunenko 2012 dataset is restricted to samples from the U.S. due to the known variation in gut microbiomes across different geographical locations. Details of how samples were subsetted for each prediction task can be found in the mlrepo-source branch of the GitHub repository, under preprocessing/make.mappings.r.

* In the same spirit, it is mentioned just after (top of page 4) that "confounders variables to control for" are reported in the tasks' metadata. This is indeed very valuable for the analysis and important to take into account. This is well explained in the Methods section, but I think it could be stressed in the main text (maybe simply by explicitly referring to the Methods section at this point).
We thank the reviewer for this suggestion and agree that more detail should be provided. We have updated the text as follows:

Hence, each prediction task is made available as an individual, compartmentalized metadata file that contains sample identifiers, responses to predict, and optionally, confounder variables that are inherent to the research study design such as paired healthy and diseased samples from the same subject (see Methods for more details).

* In the case study, it could be interesting to comment why RFs tend to do better than SVMs according to AUC but not accuracy.
We thank the reviewer for this suggestion. We have added an explanation to the text as follows:

We found that random forest accuracy improvements were moderate when compared with SVM-Linear (P=0.083) and SVM-Radial (P=0.03) [Fig 4B], which may be explained by the fact that, unlike AUC, accuracy ignores class prediction probability estimates.

* Since I assume that the number of OTUs will vary according to the nature of the samples (e.g., fewer in vaginal samples than stool samples), it could be interesting to mention in Table 1 the number of features involved in the various tasks. It could also be interesting to mention in this table whether the task involves classification or regression.

We thank the reviewer for this excellent suggestion and have added an additional table (Table 2) that describes the prediction tasks, which includes the number of samples, number of features, and the response type, as seen below. A more detailed version of this table with additional columns of metadata is also available on GitHub under web/data/tasks.txt. Note that the number of features are provided on a per-dataset basis, and not on a per-task or per-attribute basis. Microbiome abundance tables inherently contain a superset of OTUs/Taxa found across all samples within a study, and we chose to leave these tables largely intact so that the end-user can have maximum flexibility in generating new prediction tasks from the original mapping file.

* Page 6 , lines 150-153. Could we simply say that this suggests that the OTU definitions made from GreenGenes and NCBI are consistent or is it more subtle? A comment on the respective merits (if any) of the two approaches (e.g., on the number of OTUs involved or in their level of taxonomic resolution) would be useful.

We thank the reviewer for this observation, and have added text to address the noted differences between these two references databases.

Note that OTU-picking against the Greengenes database resulted in more OTU features in every dataset [Table 2], hence, these findings may also highlight how the smaller, higher-quality NCBI RefSeq database can recover the same signal from the larger Greengenes database.

* I assume that the operation consisting in "collapsing OTUs at a complete-linkage correlation of 95%" mentioned in the Methods section (page 8, line 194) has something to do with "cutting" a dendrogram built from the OTU correlation matrix. Could the authors be a bit more specific ?

We apologize for the lack of clarity and have added additional text to this sentence to detail the steps taken to collapse correlated OTUs:

...collapsed at a complete-linkage correlation of 95% (which is done by calculating the Pearson’s correlation between each pair of OTUs using all complete pairs of observations, hierarchically clustering the results, and cutting the resulting dendrogram at a height of 0.05).

* For the sake of completeness, I think it would be worth detailing a bit more in the "case study benchmarking" section how were optimized the hyper-parameters of the machine learning algorithms considered (namely the regularization parameter for the SVMs, the bandwidth of the kernel for the radial-SVM and the number of trees for the RF). For instance : which grids of parameter values were considered, whether there was some kind of "nested" cross-validation to optimize the parameters before predicting the data for the held-out data, and on which criterion (e.g., accuracy or AUC) was/were chosen the optimal parameter(s).

We thank the reviewer for pointing this out. We did not perform hyper-parameter optimization, nor grid-searching. We have updated the manuscript text to better describe the model parameter settings as follows:

Control parameters were set using the function trainControl with parameter method = ‘none’ and default parameters. Default settings for all models are as follows: SVM radial basis sigma is set to .1, all SVMs C is set to 1, and randomForest number of trees is set to 500 and number of variables to split is sqrt(p),

where p is the number of features.

Minor comments and typos :
* Page 1, line 16 : exist
Thank you for noting this. We have made this change:

Unfortunately, challenges still exist for machine learning algorithm developers who often lack domain expertise required for interpretation and curation of the heterogeneous microbiome datasets.

* Page 2 , line 38 : the term "parse" is vague.
We have changed “parse” to “interpret”, as follows:

In addition, microbiome research data can be challenging to access and analyze for expert machine learning algorithm developers, who often do not have the domain expertise required to interpret the data and metadata in complex microbiome studies.

* Page 2, line 43 : "specifically for" may be replaced by "specific to" or "dedicated to"
We have changed “specifically for” to “dedicated to”, as follows:

Currently, we are unaware of any machine learning repository dedicated to microbiome classification tasks.

* Page 2, line 50 : "using" --> "involving" ; "curated" -->"derived"
We have made the suggested changes, and the updated text is now:

We present the Microbiome Learning Repo (ML Repo), a repository of 33 curated classification and regression tasks involving human microbiome data. Our 33 tasks are derived from 15 publicly available human microbiome datasets, which include 12 amplicon-based and 3 shotgun sequencing datasets [Table 1].

* Page 3, line 53 : "developer" --> "developers"
We have made the suggested change:

These datasets vary across sequencing technology platforms, 16s hypervariable regions, and study design, in order to help developers ensure robustness of algorithms across data types.

* Page 4, line 86 : "methods develope rs" --> "method developers" (for consistency with "machine learning algorithm developers" used several times before)
We have made the suggested change:

Generally, we expect that method developers will be most interested in sweeping through the full set of prediction tasks for benchmarking, and hence would prefer to download a single compressed file containing all tasks and data.

* Page 4, line 96 : "Sample Size and Response Type" --> "sample size and response type"
We have made the suggested change:

Task pages contain descriptive details such as sample size and response type that are specific to the selected prediction task, as well as links for downloading OTU tables, taxa tables, and sample metadata [Fig 2B].

* Page 5, line 115 : the term "nuances" is vague
We apologize for the lack of clarity here, and have updated the text as follows:

The subsetted samples found in each prediction task metadata file replaces the work of rigorously deciphering metadata and understanding the subtle differences of individual research studies.

Reviewer #2:
The paper by Vangay et al. presents "ML Repo", a public repository of microbiome datasets for conducting regression and classification analysis based on machine learning approaches. The repository is a web-based service and includes currently 33 curated classification and regression tasks from 15

published human microbiome datasets. The authors presents several use cases to demonstrate its wide application. The topic involved in the paper is suitable for publication in the GigaScience journal. The manuscript is well written and well structured. In general, the paper is a nice contribution for the microbiome community.

We thank the reviewer for these comments and are pleased that the reviewer finds that our manuscript will be a contribution to the community.

However, I have some comments before a possible publication:
1. The novelty of the proposed repository needs to be better described. In particular, they authors missed to cite two recent and in some way similar repositories:
i. The "MicrobiomeHD" database, mainly for 16S studies: "Duvallet, Claire, Sean M. Gibbons, Thomas Gurry, Rafael A. Irizarry, and Eric J. Alm. "Meta-analysis of gut microbiome studies identifies disease-specific and shared responses." Nature communications 8, no. 1 (2017): 1784."
ii. The "curatedMetagenomicData" database, mainly for shotgun studies: "Pasolli, Edoardo, Lucas Schiffer, Paolo Manghi, Audrey Renson, Valerie Obenchain, Duy Tin Truong, Francesco Beghini et al. "Accessible, curated metagenomic data through ExperimentHub." Nature methods 14, no. 11 (2017): 1023."

I think these two contributions should be added in the section "Comparison to similar databases" and novelties of the proposed repository with respect to them properly discussed.

We thank the reviewer for excellent suggestion of adding these two papers, and apologize for not including them previously. We have added the following text in the section “Comparison to similar databases”:

Microbiome-based repositories that do provide manually curated metadata include curatedMetagenomicData and MicrobiomeHD. Although curatedMetagenomicData offers a collection of shotgun-metagenomics datasets with varying human sample types with gene, pathway, and taxonomic abundance tables, its data are accessible only via Bioconductor and are stored as ExpressionSet objects, which integrates metadata and abundance data. Although curatedMetagenomicData is an impressive repository with many features, it is most suitable for advanced bioinformaticians as its interface may hinder use by beginner data analysts and in teaching environments. MicrobiomeHD offers easily accessible taxonomic abundance tables with curated metadata, but is limited only to amplicon-based sequencing data, human stool samples, and case-control responses. And although both curatedMetagenomicData and MicrobiomeHD provide manually curated metadata, biological interpretation is still required as other sample metadata, for example antibiotic usage, may have biological relevance in predicting responses. This poses a potential problem for machine learning developers with limited biological and microbiome domain expertise. MLRepo resolves this issue by explicitly defining classification and regressions tasks for predicting responses that have been manually curated to either remove confounders or have been specifically annotated with biological confounders that must be controlled for. Metadata files in MLRepo are task-specific, and hence, are simplified to contain only: (1) sample identifiers indicating samples that should be used for the prediction task, (2) corresponding high-level phenotypes or responses, and optionally, (3) a confounder that should be accounted for due to its biological relevance. In addition, datasets in MLRepo include both amplicon-based and shotgun-metagenomics datasets covering a variety of human sample types, and are easily accessible via a web-interface.

2. The repository aims at providing metadata for both classification and regression tasks, as explicitly written also in the title. However, from my understanding use cases were reported on classification tasks only. Could you add some example on regression tasks?

We thank the reviewer for this suggestion. For space limitation reasons we have not added a whole section demonstrating a parameter sweep on the regression tasks. However, our work demonstrates that sweeping across parameters can inform future machine learning development efforts in regression, and we have emphasized the inclusion of regression tasks throughtout the mansucript.

3. Do you expect to add new datasets in the future? Can users contribute to them? Please describe better this aspect in the paper and potentially on the website.

We do expect to add new datasets in the future, and also allow users to contribute to our repository. We

apologize for not making the instructions more explicit. We have updated this section of our manuscript to point to the instructions for adding new datasets. We have provided the contents of https://github.com/knights-lab/MLRepo/blob/master/add-datasets-readme.md below (note that when viewed on GitHub, words referring to tools, databases, or GitHub tasks are hyperlinked to respective websites with instructions):
Steps for submitting a new dataset and/or task
1. If you have either the raw FASTQ or processed FASTA file, please deposit it into a public repository. We list large files via publicly accessible URLs and do not support uploading of any large files. If you need assistance, please contact us.
2. If starting with FASTQ, we recommend processing with SHI7 and OTU-picking with BURST, with NCBI RefSeq Prokaryote files and Green genes 97
3. Fork our repository.
4. Add new tasks and datasets directly into tasks and datasets. Make sure to fill out all sections.
5. We expect you to apply rigorous standards in filtering, subsetting, and selecting samples for your classification and regression tasks.
6. When ready, submit a pull request for our review.

We provide instructions on our GitHub repository (https://github.com/knights-lab/MLRepo/blob/master/add-datasets-readme.md) to guide users to create a fork from our repository, add the appropriate data and files, and update the master task and dataset lists.

4. Line 75: "Well-known confounders, such as geography, were accounted for when constructing prediction tasks for other human-associated conditions". I did not understand how this was really implemented in your analysis.

We thank the reviewer for pointing this out. We have updated the text below to better explain how we subset samples to address confounders. We have also provided the location of the R script that shows how we processed each original metadata file.

Well-known confounders were accounted for when constructing prediction tasks for other human-associated conditions; for example, predicting age using the Yatsunenko 2012 dataset is restricted to samples from the U.S. due to the known variation in gut microbiomes across different geographical locations. Details of how samples were subsetted for each prediction task can be found in the mlrepo-source branch of the GitHub repository, under preprocessing/make.mappings.r.

Reviewer #3:
In this contribution the authors present a repository of machine learning tasks, or 'challenges', concerning the prediction of a range of (human) host phenotypes from the composition of (one of) its microbiome(s). Other, non-phenotypical responses are concerned with host geographic location, body habitat, diet or antibiotic treatment. In general, this effort is highly appreciated, since the collection of suitable benchmark datasets and their standardisation is - at least - 50% of the work when developing machine learning (and other, baseline) methods.

The manuscript as a whole is in good shape and I specifically like the figures. My only real concern - and that's where the minor corrections come in - would be the general understandability of the manuscript to a non-microbiome audience, specifically to the envisaged (as one user type) CS-type user. The comparison to the UCI machine learning repository illustrates this concern best: what if a machine learning expert lacking _any_ biological background (i.e. not a microbiome-bioinformatics nor a bioinformatics but 'merely' an informatics person) was interested in your datasets and would read the paper as an introduction to using the data? Even with a bioinformatics background, while one will generally have heard about most of the basic concepts, it couldn't hurt to be reminded with some short additional explanations in the right places. I try to list those places in the following:

p3,l56: 'We preprocessed raw sequences using...' - what do these tools do i.e. what does 'preprocessing' refer to exactly in this case?

We apologize for the lack of detail here and have updated the manuscript text to the following:

Raw sequences were trimmed and quality filtered using SHI7 [12] or QIIME [13].

p3,l59: 'We picked Operational Taxonomic Units (OTUs)...' - maybe briefly explain what this is, mainly

the difference to the taxa counts

We thank the reviewer and agree that a definition is warranted. We have created a Glossary where we define OTU as follows:

OTU Operational Taxonomic Unit, group of closely related organisms based on DNA sequence similarity.

p3,l72: 'When available, published study exclusion criteria was [were!] applied accordingly...' - an example of such exclusion criteria would explain what you mean in an instant, just like you already do for the confounders

We thank the reviewer for the suggestion, and have updated the text as follows:

When available, published study exclusion criteria, such as reported use of antibiotics, were applied accordingly and confounders were removed by dropping samples or stratification.

p3,l77: '...to minimize the effect of high intra-individual similarities.' - it's not immediately obvious what you mean here, maybe rephrase? how does longitudinal data come in at all for your type of tasks? from reading it once i had the impression you'd have to select only one time point as the dataset for any given task anyway? i may well be missing a point here, so just make sure you spell it out as simple as you can.

We apologize for the lack of clarity and have replaced this text. We hope that the following text does a better job of explaining how we reduced the number of samples per subject.

Studies that were cross-sectional by design but contained several samples per subject were filtered to contain one sample per subject. In study designs with paired diseased-healthy or pre- and post-intervention samples, samples were reduced to two samples per subject with subject identifiers provided as confounder variables.

p8,l193: '...collapsed at a complete-linkage correlation of 95%.' - i totally didn't get this, it's definitely st you can save lots of (non-microbiome) people the time to think about by adding a short explanation

We apologize for the lack of clarification here and have updated the text as follows:

...collapsed at a complete-linkage correlation of 95% (which is done by calculating the Pearson’s correlation between each pair of OTUs using all complete pairs of observations, hierarchically clustering the results, and cutting the resulting dendrogram at a height of 0.05).

This may or may not be exhaustive but I hope you do get the general point. I think the necessary additions are rather small, st rephrasing may be sufficient, st just one more sentence. I suppose the 'luxury' solution would be having those amendmends done to the manuscript _and_ providing a glossary page on your website, just for the 5-10 relevant terms - but whether or not that additional effort makes sense for you and your target audience is st only you can decide. I would definitely hope that, with the suggest additional explanations, the manuscript and therefore resource may be helpful for a slightly wider audience than without them.

We greatly appreciate the suggestions that the reviewer has made, and have also added the glossary below to assist with some of these concerns.

OTU Operational Taxonomic Unit, group of closely related organisms based on DNA sequence similarity.
16S 16S ribosomal RNA gene, component of the prokaryotic ribosome, used to reconstruct phylogenies.
FASTA Text-based format for representing nucleotide sequences with single-letter codes.
FASTQ Text-based format for representing nucleotide sequences and corresponding quality scores, with single-letter codes for nucleotides and quality.
Taxa Groups of one or more populations of organisms. Usually summarized at phylum, class, order, family, genus, or species levels.
Metadata Descriptive data pertaining to samples within a study
Shotgun Shotgun metagenomics sequencing breaks up all available DNA into random small segments and uses chain termination to sequence reads. Reads can be aligned directly to a reference database, or overlapping reads can be assembled into contiguous sequences.

Let me finish with some further, random points, in order of appearance:

general 1: Should already the abstract contain a link to your website? It's often done like that.

We have added the website URL to the abstract:
We present Microbiome Learning Repo (ML Repo, available at https://knights-lab.github.io/MLRepo/), a public, web-based repository of 33 curated classification and regression tasks from 15 published human microbiome datasets.

general 2: I was wondering whether or not the term 'task' could generally be replaced by 'challenge' to make it more clear?

We have decided not to change the term 'task' because this term is commonly used in the machine-learning community, especially when referring to prediction tasks, and we want to make the manuscript as accessible as possible to the machine learning community.

p3,l53: developer -> developers

This text has been updated as suggested.

p3,l56: I don't think the 'n=number' style of writing is necessary or in any way beneficial to the reader, so just put the number. This is true for all its occurrences throughout the manuscript.

We have removed 'n=' throughout the manuscript.

p3,l62: I may get it wrong but isn't the 'per sample' redundant here? Or do the two 'sample'-s in the first part of the sentence refer to two different things? And what does 'depths' mean? If it's just the number of sequences then 'Samples with less than...' would do the job and make reading easier. This issue recurs at least once below. So fix both in case.

We have updated the text as follows:

Samples with less than 1000 sequencing reads were dropped for 10 datasets, while we applied a lower threshold of 100 sequencing reads per sample for 5 datasets which had lower expected bacterial load.

p4,l96: You use uppercase very sparingly, can't see why to use it in 'Sample Size and Response Type' here.

This text has been updated as suggested.

p5,l105: Use it here, in 'Machine Learning Repository' instead, esp. given you do use it when mentioning this resource first.

This text has been updated as suggested.

p5,l114: Can't you just scrap the 'prediction tasks.' here?

Yes, 'prediction tasks' have been removed from this text.

p5,l115: replaces -> replace

This text has been updated as suggested.

p5,l116: I don't quite understand how the 'Hence...' logically connects this sentence with the preceding one. Like, what have both to do with each other?

We agree with this observation. We have removed 'Hence' from this text.

p5,l125: Is it, logically speaking, not already 'for assigning' i.e. when you come up with high-level binary classes that later make your responses for prediction?

We have added extensive detail to this section describing what features discriminate ML Repo from, and

make it more amenable to use by machine learning experts than, other efforts to collate microbiome data.

p6,l140: Here you comment on AUC being generally accepted etc, however, you already use it (without such a comment) earlier in the same para. Maybe revise slightly.

We thank the reviewer for pointing this out. We have moved this comment to the first mention of AUC, as follows:
Sweeping through available tasks with binary responses, we compare our models by examining receiver operating curves (ROCs) and areas under the curve (AUC), considered the standard method for machine learning model evaluation [23,24] [Fig 3].

p7,l160: This needs rephrasing! Right now you 'accept and merge' the actual researchers into your repo - they may not like this :)

We apologize for this incorrect phrasing. We have updated the text as follows:
Researchers can then submit a pull request for our review, and requests that are properly formatted will be accepted and merged into the repository

p8,l195: 'with smaller sample sizes' -> 'with fewer samples'?

This text has been updated as suggested.

One last important bit I just noticed: the mapping-orig.txt type links do not work for me as of 21/09/18 and must be fixed, e.g. https://knights-lab.github.io/MLRepo/docs/datasets/claesson/mapping-orig.txt.

We thank the reviewer for catching this important broken link. We have fixed our code and updated all of the original mapping file links. They should all be working now.

Close
