# Supplementary material for: Microbiome Learning Repo (ML Repo): A public repository of microbiome regression and classification tasks
Source: Gigascience. 2019 Apr 26;8(5):giz042. doi: 10.1093/gigascience/giz042 (PMC6493971; doi:10.1093/gigascience/giz042)
Supplement: Reviewer_1_Report_Original_Submission -- Pierre Mahe — 9/20/2018 Reviewed [file giz042_reviewer_1_report_original_submission.pdf]

Reviewer Report

Title: Microbiome Learning Repo (ML Repo): A public repository of microbiome regression and classification tasks

Version: Original Submission **Date:** 9/20/2018

Reviewer name: Pierre Mahe

Reviewer Comments to Author:

This paper describes MLRepo, a database of standardized microbiome datasets to develop and evaluate machine learning algorithms. The strength of this resource is to provide machine learning researchers a panel of diverse datasets (e.g., regression and classification tasks of various levels of complexity and with a various number of samples), already curated and formatted, to evaluate in an objective way novel algorithms dedicated to the analysis of microbiome samples. It can also be useful for teaching purposes (e.g., for practical lab sessions or to set up "data challenges") and will obviously be valuable to the community of microbiome researchers to set-up meta-analyses.

Overall I am very enthusiastic about this repository. As a machine-learning method developer interested in microbiome / metagenomics applications, I am indeed well aware that building curated databases and setting up baselines to evaluate novel algorithms is very time consuming, and that results reported in published papers are sometimes hard to reproduce. I am therefore convinced that this repository will simplify the whole process and facilitate evaluating algorithms in an objective way. I am therefore very favorable in having this work published in Giga Science.

My main comment comes as a suggestion. While the paper is relatively easy to follow for someone already aware of microbiome / metagenomics studies, some additional information may be useful to users of the database not familiar (at all) with metagenomics data. In particular (i) a glossary of technical terms specific to (meta)genomics data and analysis (e.g., OTU, 16s, fasta/fastq), and (ii) some additional details regarding the format of the data provided, especially the taxonomic information provided in the "taxatable" files. In the same spirit, it could be interesting to highlight an important specificity of microbiome data, namely that the input variables (taxa/OTUS) are ordered in a hierarchy. Dedicated machine learning methods exist (or could be developed) to take into account this type of data. Altogether, this could further motivate machine learning researchers interested in the analysis of structured data to use this repository.

Besides this general comment, I have a few questions that may deserve some clarifications in the main text :

* It is mentioned in page 3 that "full details regarding the data processing are provided for each dataset in the repository", but I am not sure to find them.
* I am not sure to understand what is meant by "samples with depths lower than 1000 sequences per samples were dropped". Do these 1000 sequences correspond to reads ? or to contigs ?
* This may be obvious but I am not sure to understand how are defined OTUs from shotgun sequencing data. Are they based on the 16s gene only or is the entire genome used somehow?

* It is mentioned in page 3 that (i) "confounders were removed by dropping samples or stratification and (ii) "well-known confounders […] were accounted for when constructing prediction tasks". Could the authors be more specific about these (important) steps ?
* In the same spirit, it is mentioned just after (top of page 4) that "confounders variables to control for" are reported in the tasks' metadata. This is indeed very valuable for the analysis and important to take into account. This is well explained in the Methods section, but I think it could be stressed in the main text (maybe simply by explicitly referring to the Methods section at this point).
* In the case study, it could be interesting to comment why RFs tend to do better than SVMs according to AUC but not accuracy.
* Since I assume that the number of OTUs will vary according to the nature of the samples (e.g., fewer in vaginal samples than stool samples), it could be interesting to mention in Table 1 the number of features involved in the various tasks. It could also be interesting to mention in this table whether the task involves classification or regression.
* Page 6 , lines 150-153. Could we simply say that this suggests that the OTU definitions made from GreenGenes and NCBI are consistent or is it more subtle? A comment on the respective merits (if any) of the two approaches (e.g., on the number of OTUs involved or in their level of taxonomic resolution) would be useful.
* I assume that the operation consisting in "collapsing OTUs at a complete-linkage correlation of 95%" mentioned in the Methods section (page 8, line 194) has something to do with "cutting" a dendrogram built from the OTU correlation matrix. Could the authors be a bit more specific ?
* For the sake of completeness, I think it would be worth detailing a bit more in the "case study benchmarking" section how were optimized the hyper-parameters of the machine learning algorithms considered (namely the regularization parameter for the SVMs, the bandwidth of the kernel for the radial-SVM and the number of trees for the RF). For instance : which grids of parameter values were considered, whether there was some kind of "nested" cross-validation to optimize the parameters before predicting the data for the held-out data, and on which criterion (e.g., accuracy or AUC) was/were chosen the optimal parameter(s).
Minor comments and typos :
* Page 1, line 16 : exist
* Page 2 , line 38 : the term "parse" is vague.
* Page 2, line 43 : "specifically for" may be replaced by "specific to" or "dedicated to"
* Page 2, line 50 : "using" --> "involving" ; "curated" -->"derived"
* Page 3, line 53 : "developer" --> "developers"
* Page 4, line 86 : "methods developers" --> "method developers" (for consistency with "machine learning algorithm developers" used several times before)
* Page 4, line 96 : "Sample Size and Response Type" --> "sample size and response type"
* Page 5, line 115 : the term "nuances" is vague

Level of Interest

Please indicate how interesting you found the manuscript: Choose an item.

Quality of Written English

Please indicate the quality of language in the manuscript: Choose an item.

Declaration of Competing Interests

Please complete a declaration of competing interests, considering the following questions:

- Have you in the past five years received reimbursements, fees, funding, or salary from an organisation that may in any way gain or lose financially from the publication of this manuscript, either now or in the future?
- Do you hold any stocks or shares in an organisation that may in any way gain or lose financially from the publication of this manuscript, either now or in the future?
- Do you hold or are you currently applying for any patents relating to the content of the manuscript?
- Have you received reimbursements, fees, funding, or salary from an organization that holds or has applied for patents relating to the content of the manuscript?
- Do you have any other financial competing interests?
- Do you have any non-financial competing interests in relation to this paper?

If you can answer no to all of the above, write 'I declare that I have no competing interests' below. If your reply is yes to any, please give details below.

I declare that I have no competing interests.

I agree to the open peer review policy of the journal. I understand that my name will be included on my report to the authors and, if the manuscript is accepted for publication, my named report including any attachments I upload will be posted on the website along with the authors' responses. I agree for my report to be made available under an Open Access Creative Commons CC-BY license (http://creativecommons.org/licenses/by/4.0/). I understand that any comments which I do not wish to be included in my named report can be included as confidential comments to the editors, which will not be published.

I agree to the open peer review policy of the journal

To further support our reviewers, we have joined with Publons, where you can gain additional credit to further highlight your hard work (see: https://publons.com/journal/530/gigascience). On publication of this paper, your review will be automatically added to Publons, you can then choose whether or not to claim your Publons credit. I understand this statement.

Yes Choose an item.
