# Supplementary material for: Microbiome Learning Repo (ML Repo): A public repository of microbiome regression and classification tasks
Source: Gigascience. 2019 Apr 26;8(5):giz042. doi: 10.1093/gigascience/giz042 (PMC6493971; doi:10.1093/gigascience/giz042)
Supplement: Reviewer_2_Report_Original_Submission -- Edoardo Pasolli — 9/21/2018 Reviewed [file giz042_reviewer_2_report_original_submission.pdf]

Reviewer Report

Title: Microbiome Learning Repo (ML Repo): A public repository of microbiome regression and classification tasks

Version: Original Submission **Date:** 9/21/2018

Reviewer name: Edoardo Pasolli

Reviewer Comments to Author:

The paper by Vangay et al. presents "ML Repo", a public repository of microbiome datasets for conducting regression and classification analysis based on machine learning approaches.
The repository is a web-based service and includes currently 33 curated classification and regression tasks from 15 published human microbiome datasets.
The authors presents several use cases to demonstrate its wide application.
The topic involved in the paper is suitable for publication in the GigaScience journal.
The manuscript is well written and well structured.
In general, the paper is a nice contribution for the microbiome community. However, I have some comments before a possible publication:
1. The novelty of the proposed repository needs to be better described. In particular, they authors missed to cite two recent and in some way similar repositories:
i. The "MicrobiomeHD" database, mainly for 16S studies: "Duvallet, Claire, Sean M. Gibbons, Thomas Gurry, Rafael A. Irizarry, and Eric J. Alm. "Meta-analysis of gut microbiome studies identifies disease-specific and shared responses." Nature communications 8, no. 1 (2017): 1784."
ii. The "curatedMetagenomicData" database, mainly for shotgun studies: "Pasolli, Edoardo, Lucas Schiffer, Paolo Manghi, Audrey Renson, Valerie Obenchain, Duy Tin Truong, Francesco Beghini et al. "Accessible, curated metagenomic data through ExperimentHub." Nature methods 14, no. 11 (2017): 1023."
I think these two contributions should be added in the section "Comparison to similar databases" and novelties of the proposed repository with respect to them properly discussed.
2. The repository aims at providing metadata for both classification and regression tasks, as explicitly written also in the title. However, from my understanding use cases were reported on classification tasks only. Could you add some example on regression tasks?
3. Do you expect to add new datasets in the future? Can users contribute to them? Please describe better this aspect in the paper and potentially on the website.
4. Line 75: "Well-known confounders, such as geography, were accounted for when constructing prediction tasks for other human-associated conditions". I did not understand how this was really implemented in your analysis.

Level of Interest

Please indicate how interesting you found the manuscript: Choose an item.

Quality of Written English

Please indicate the quality of language in the manuscript: Choose an item.

Declaration of Competing Interests

Please complete a declaration of competing interests, considering the following questions:

- Have you in the past five years received reimbursements, fees, funding, or salary from an organisation that may in any way gain or lose financially from the publication of this manuscript, either now or in the future?
- Do you hold any stocks or shares in an organisation that may in any way gain or lose financially from the publication of this manuscript, either now or in the future?
- Do you hold or are you currently applying for any patents relating to the content of the manuscript?
- Have you received reimbursements, fees, funding, or salary from an organization that holds or has applied for patents relating to the content of the manuscript?
- Do you have any other financial competing interests?
- Do you have any non-financial competing interests in relation to this paper?

If you can answer no to all of the above, write 'I declare that I have no competing interests' below. If your reply is yes to any, please give details below.

I declare that I have no competing interests

I agree to the open peer review policy of the journal. I understand that my name will be included on my report to the authors and, if the manuscript is accepted for publication, my named report including any attachments I upload will be posted on the website along with the authors' responses. I agree for my report to be made available under an Open Access Creative Commons CC-BY license (http://creativecommons.org/licenses/by/4.0/). I understand that any comments which I do not wish to be included in my named report can be included as confidential comments to the editors, which will not be published.

I agree to the open peer review policy of the journal

To further support our reviewers, we have joined with Publons, where you can gain additional credit to further highlight your hard work (see: https://publons.com/journal/530/gigascience). On publication of this paper, your review will be automatically added to Publons, you can then choose whether or not to claim your Publons credit. I understand this statement.

Yes Choose an item.
