# Supplementary material for: Microbiome Learning Repo (ML Repo): A public repository of microbiome regression and classification tasks
Source: Gigascience. 2019 Apr 26;8(5):giz042. doi: 10.1093/gigascience/giz042 (PMC6493971; doi:10.1093/gigascience/giz042)
Supplement: Reviewer_3_Report_Original_Submission -- Robert Rentzsch, Ph.D. — 9/21/2018 Reviewed [file giz042_reviewer_3_report_original_submission.pdf]

**Reviewer Report**

**Title: Microbiome Learning Repo (ML Repo): A public repository of microbiome regression and classification tasks**

**Version: Original Submission** **Date:** 9/21/2018

**Reviewer name: Robert Rentzsch, Ph.D.**

**Reviewer Comments to Author:**

In this contribution the authors present a repository of machine learning tasks, or 'challenges', concerning the prediction of a range of (human) host phenotypes from the composition of (one of) its microbiome(s). Other, non-phenotypical responses are concerned with host geographic location, body habitat, diet or antibiotic treatment. In general, this effort is highly appreciated, since the collection of suitable benchmark datasets and their standardisation is - at least - 50% of the work when developing machine learning (and other, baseline) methods.
The manuscript as a whole is in good shape and I specifically like the figures. My only real concern - and that's where the minor corrections come in - would be the general understandability of the manuscript to a non-microbiome audience, specifically to the envisaged (as one user type) CS-type user. The comparison to the UCI machine learning repository illustrates this concern best: what if a machine learning expert lacking _any_ biological background (i.e. not a microbiome-bioinformatics nor a bioinformatics but 'merely' an informatics person) was interested in your datasets and would read the paper as an introduction to using the data? Even with a bioinformatics background, while one will generally have heard about most of the basic concepts, it couldn't hurt to be reminded with some short additional explanations in the right places. I try to list those places in the following:
p3,l56: 'We preprocessed raw sequences using...' - what do these tools do i.e. what does 'preprocessing' refer to exactly in this case?
p3,l59: 'We picked Operational Taxonomic Units (OTUs)...' - maybe briefly explain what this is, mainly the difference to the taxa counts
p3,l72: 'When available, published study exclusion criteria was [were!] applied accordingly...' - an example of such exclusion criteria would explain what you mean in an instant, just like you already do for the confounders
p3,l77: '...to minimize the effect of high intra-individual similarities.' - it's not immediately obvious what you mean here, maybe rephrase? how does longitudinal data come in at all for your type of tasks? from reading it once i had the impression you'd have to select only one time point as the dataset for any given task anyway? i may well be missing a point here, so just make sure you spell it out as simple as you can.
p8,l193: '...collapsed at a complete-linkage correlation of 95%.' - i totally didn't get this, it's definitely st you can save lots of (non-microbiome) people the time to think about by adding a short explanation
This may or may not be exhaustive but I hope you do get the general point. I think the necessary additions are rather small, st rephrasing may be sufficient, st just one more sentence. I suppose the 'luxury' solution would be having those amendmends done to the manuscript _and_ providing a glossary page on your website, just for the 5-10 relevant terms - but whether or not that additional effort makes

sense for you and your target audience is st only you can decide. I would definitely hope that, with the suggest additional explanations, the manuscript and therefore resource may be helpful for a slightly wider audience than without them.
Let me finish with some further, random points, in order of appearance:
general 1: Should already the abstract contain a link to your website? It's often done like that.
general 2: I was wondering whether or not the term 'task' could generally be replaced by 'challenge' to make it more clear?
p3,l53: developer -> developers
p3,l56: I don't think the 'n=number' style of writing is necessary or in any way beneficial to the reader, so just put the number. This is true for all its occurrences throughout the manuscript.
p3,l62: I may get it wrong but isn't the 'per sample' redundant here? Or do the two 'sample'-s in the first part of the sentence refer to two different things? And what does 'depths' mean? If it's just the number of sequences then 'Samples with less than...' would do the job and make reading easier. This issue recurs at least once below. So fix both in case.
p4,l96: You use uppercase very sparingly, can't see why to use it in 'Sample Size and Response Type' here.
p5,l105: Use it here, in 'Machine Learning Repository' instead, esp. given you do use it when mentioning this resource first.
p5,l114: Can't you just scrap the 'prediction tasks.' here?
p5,l115: replaces -> replace
p5,l116: I don't quite understand how the 'Hence...' logically connects this sentence with the preceding one. Like, what have both to do with each other?
p5,l125: Is it, logically speaking, not already 'for assigning' i.e. when you come up with high-level binary classes that later make your responses for prediction?
p6,l140: Here you comment on AUC being generally accepted etc, however, you already use it (without such a comment) earlier in the same para. Maybe revise slightly.
p7,l160: This needs rephrasing! Right now you 'accept and merge' the actual researchers into your repo - they may not like this :)
p8,l195: 'with smaller sample sizes' -> 'with fewer samples'?
One last important bit I just noticed: the mapping-orig.txt type links do not work for me as of 21/09/18 and must be fixed, e.g. https://knights-lab.github.io/MLRepo/docs/datasets/claesson/mapping-orig.txt.

**Level of Interest**

Please indicate how interesting you found the manuscript: Choose an item.

**Quality of Written English**

Please indicate the quality of language in the manuscript: Choose an item.

**Declaration of Competing Interests**

Please complete a declaration of competing interests, considering the following questions:

- Have you in the past five years received reimbursements, fees, funding, or salary from an organisation that may in any way gain or lose financially from the publication of this manuscript, either now or in the future?
- Do you hold any stocks or shares in an organisation that may in any way gain or lose financially from the publication of this manuscript, either now or in the future?
- Do you hold or are you currently applying for any patents relating to the content of the manuscript?
- Have you received reimbursements, fees, funding, or salary from an organization that holds or has applied for patents relating to the content of the manuscript?
- Do you have any other financial competing interests?
- Do you have any non-financial competing interests in relation to this paper?

If you can answer no to all of the above, write 'I declare that I have no competing interests' below. If your reply is yes to any, please give details below.

I declare that I have no competing interests

I agree to the open peer review policy of the journal. I understand that my name will be included on my report to the authors and, if the manuscript is accepted for publication, my named report including any attachments I upload will be posted on the website along with the authors' responses. I agree for my report to be made available under an Open Access Creative Commons CC-BY license (http://creativecommons.org/licenses/by/4.0/). I understand that any comments which I do not wish to be included in my named report can be included as confidential comments to the editors, which will not be published.

I agree to the open peer review policy of the journal

To further support our reviewers, we have joined with Publons, where you can gain additional credit to further highlight your hard work (see: https://publons.com/journal/530/gigascience). On publication of this paper, your review will be automatically added to Publons, you can then choose whether or not to claim your Publons credit. I understand this statement.

Yes Choose an item.
